# Supplementary material for: Hydrodenitrogenation of pyridines and quinolines at a multinuclear titanium hydride framework
Source: Nat Commun. 2017 Nov 30;8:1866. doi: 10.1038/s41467-017-01607-z (PMC5709410; doi:10.1038/s41467-017-01607-z)
Supplement: Supplementary file 1 — Supplementary Information [file 41467_2017_1607_MOESM1_ESM.pdf]

Supplementary Methods

General Considerations

All reactions were carried out under a dry and oxygen-free argon atmosphere by using Schlenk techniques or under an argon atmosphere in an Mbraun glovebox. The argon was purified by being passed through a Dryclean column (4A molecular sieves, Nikka Seiko Co.) and a Gasclean GC-XR column (Nikka Seiko Co.). The argon in the glovebox was constantly circulated through a copper/molecular sieves catalyst unit. The oxygen and moisture concentrations in the glovebox atmosphere were monitored by an O_2/H_2O Combi-Analyzer (Mbraun) to ensure both were always below 1 ppm. IR spectra were recorded on a Thermo Nicolet 380 spectrometer using nujol mulls between KBr disks. Samples for NMR spectroscopic measurements were prepared in the glovebox by use of J. Young valve NMR tubes. ^{1}H and ^{13}C NMR spectrum were recorded on a JEOL JNM-ECS400, or a Bruker Advance 500HD spectrometer. Anhydrous THF, hexane, benzene, and toluene were purified by use of a SPS-800 solvent purification system (Mbraun), and dried over fresh Na chips in the glovebox. $[(C_5Me_4SiMe_3)Ti]_3(\mu_3\text{-H})(\mu_2\text{-H})_6$ (**1**) was prepared according to literature procedures[1] and was stored in the glovebox. Ultrapure water was purchased from Wako and was degassed and stored under Ar atmosphere. Ammonia was detected by ^{1}H NMR spectroscopy in DMSO-d_6 after being transformed to NH_4Cl by reaction with HCl[2] as well as by phenol-hypochlorite titration[3].

Reaction of Complex 1 with Aromatic *N*-heterocycles

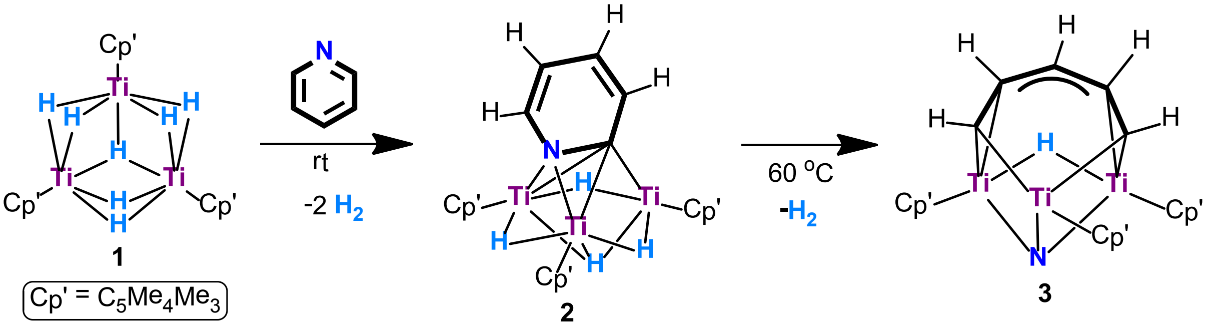

$[(C_5Me_4SiMe_3)Ti]_3(\mu\text{-}\eta^1\text{:}\eta^2\text{:}\eta^2\text{-}C_5H_4N)(\mu_2\text{-H})_3(\mu_3\text{-H})$ (2). In an Ar glovebox, pyridine (15 mg, 0.19 mmol) was slowly added to a hexane solution (5.0 mL) of $[(C_5Me_4SiMe_3)Ti]_3(\mu_3\text{-H})(\mu_2\text{-H})_6$ (**1**) (100 mg, 0.14 mmol). The mixture was stirred at room temperature for 10 min, and an immediate color change from dark green to dark purple was observed. The solution was concentrated and cooled at –33 °C to give **2** as dark-purple crystals (105 mg, 0.13 mmol, 93% yield). Single crystals suitable for X-ray diffraction studies were obtained by recrystallization from a hexane solution at –33 °C. The preparation of the ^{15}N-enriched compound $[(C_5Me_4SiMe_3)Ti]_3(\mu\text{-}\eta^1\text{:}\eta^2\text{:}\eta^2\text{-}C_5H_4{}^{15}N)(\mu_2\text{-H})_3(\mu_3\text{-H})$ (**2-^{15}N**) was carried out in the same manner.

^{1}H NMR (THF-d_8, –60 °C): 8.13 (d, $J_{H,H}$ = 4.0 Hz, 1H, C_5H_4N), 7.90 (d, $J_{H,H}$ = 16.0 Hz, 1H, μ_2-H), 6.53 (d, $J_{H,H}$ = 8.0 Hz, 1H, C_5H_4N), 5.84 (t, $J_{H,H}$ = 4.0 Hz, 1H, C_5H_4N), 5.18 (dd, $J_{H,H}$ = 4.0 Hz, 8.0 Hz, 1H , C_5H_4N), 1.35 (d, $J_{H,H}$ = 8.0 Hz, 2H, μ_2-H), 2.57, 2.50, 2.28, 2.08, 1.93, 1.66 (s, 6H×6, $C_5Me_4SiMe_3$), 0.21 (s, 18H, $C_5Me_4SiMe_3$), 0.06 (s, 9H, $C_5Me_4SiMe_3$), –6.10 (td, $J_{H,H}$ = 16.0 Hz, 8.0 Hz, 1H, μ_3-H).

^{13}C NMR (THF-d_8, rt): 186.4 (s, C_5H_4N), 150.9 (s, C_5H_4N), 145.6 (s, C_5H_4N), 127.9, 120.8 (s, $C_5Me_4SiMe_3$), 112.7 (s, *ipso*-$C_5Me_4SiMe_3$), 110.8 (s, C_5H_4N), 109.4 (s, C_5H_4N), 17.1, 12.4 (s, $C_5Me_4SiMe_3$), 3.0 (s, $C_5Me_4SiMe_3$).

^{13}C NMR (C_6D_6, rt): 185.8 (s, C_5H_4N), 150.1 (s, C_5H_4N), 145.3 (s, C_5H_4N), 127.7, 120.6 (s, $C_5Me_4SiMe_3$), 112.6 (s, *ipso*-$C_5Me_4SiMe_3$), 110.4 (s, C_5H_4N), 108.9 (s, C_5H_4N), 17.2, 12.5 (s, $C_5Me_4SiMe_3$), 3.2 (s, $C_5Me_4SiMe_3$).

Calcd for $C_{41}H_{71}NSi_3Ti_3$: C, 61.11; H, 8.88, N, 1.74. Found: C, 61.27; H, 8.72, N, 2.04.

^{15}N NMR of **2-^{15}N** (60.81 MHz, THF-d_8, $MeNO_2$, –60 °C): δ –122.7 (s)

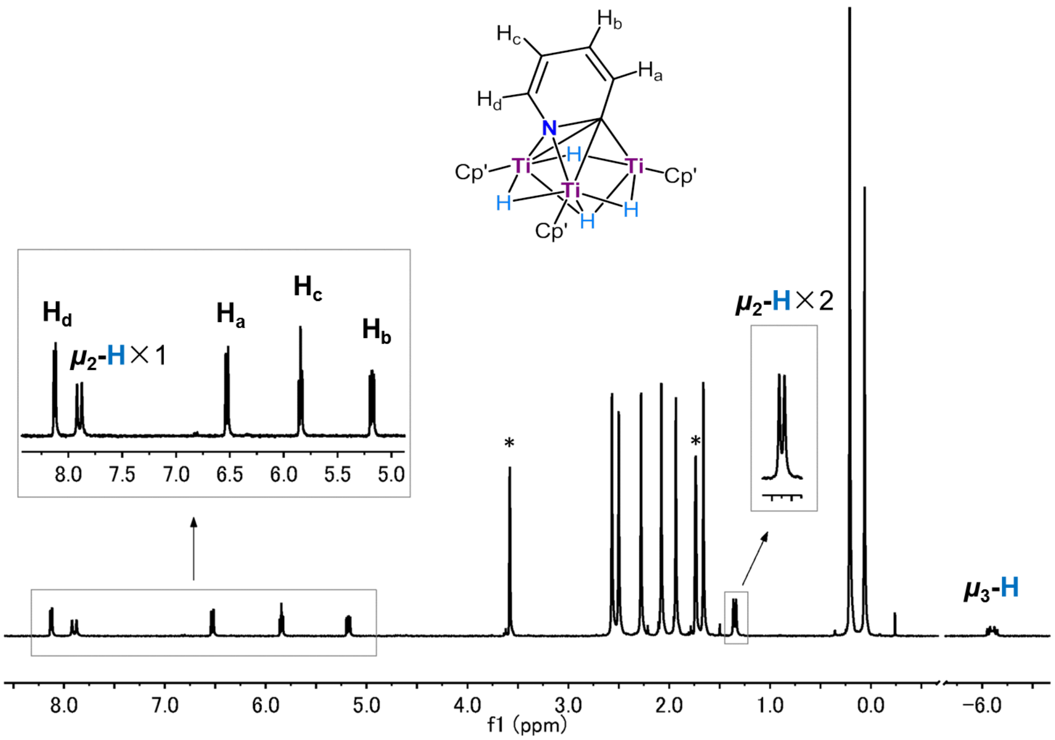

Supplementary Fig. 1 | ^{1}H NMR spectrum of 2 (THF-d_8, –60 ^{o}C, * residual signals of THF-d_8)

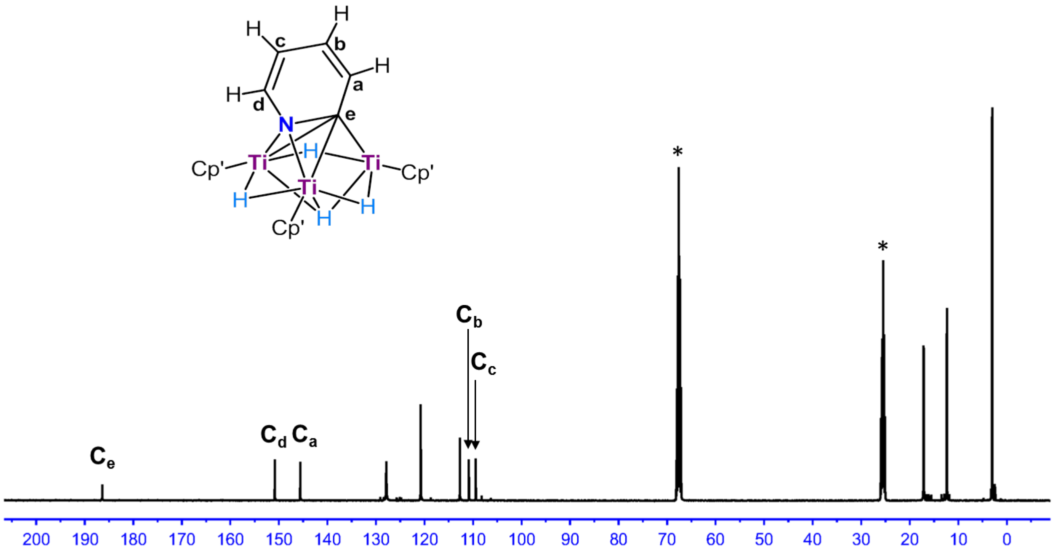

Supplementary Fig. 2 | ^{13}C NMR spectrum of 2 (THF-d_8, rt, * residual signals of THF-d_8)

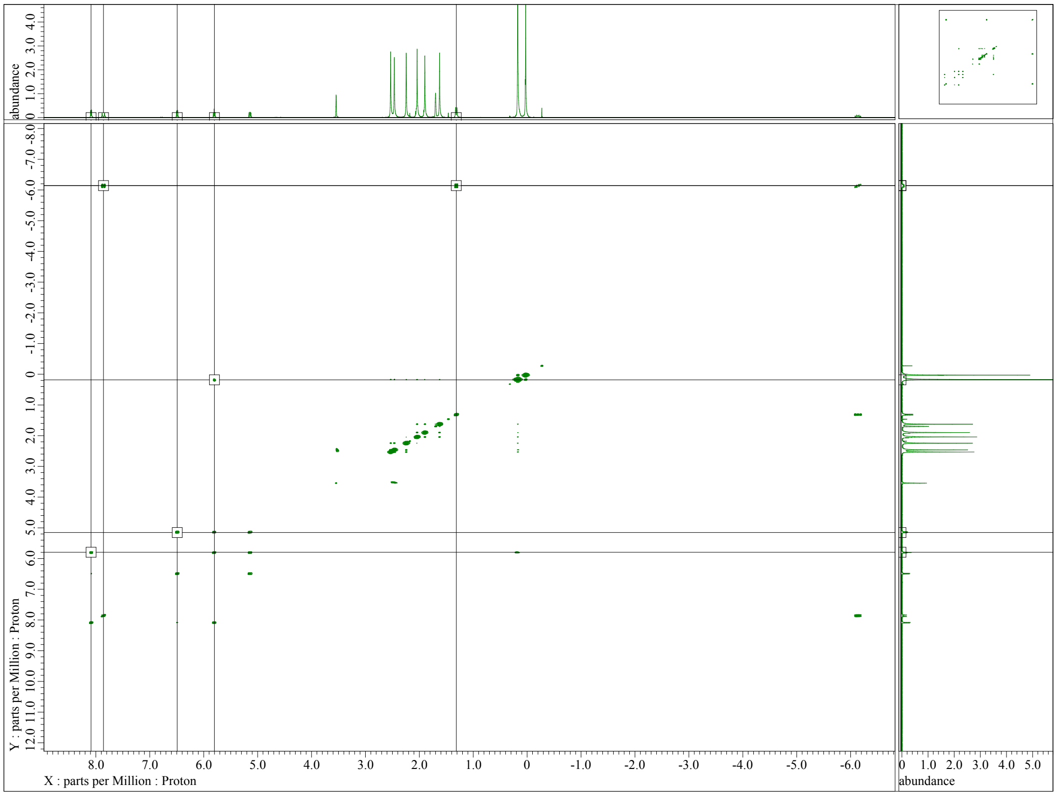

Supplementary Fig. 3 | H-H COSY NMR spectrum of 2 (THF-d_8, –60 ^{o}C)

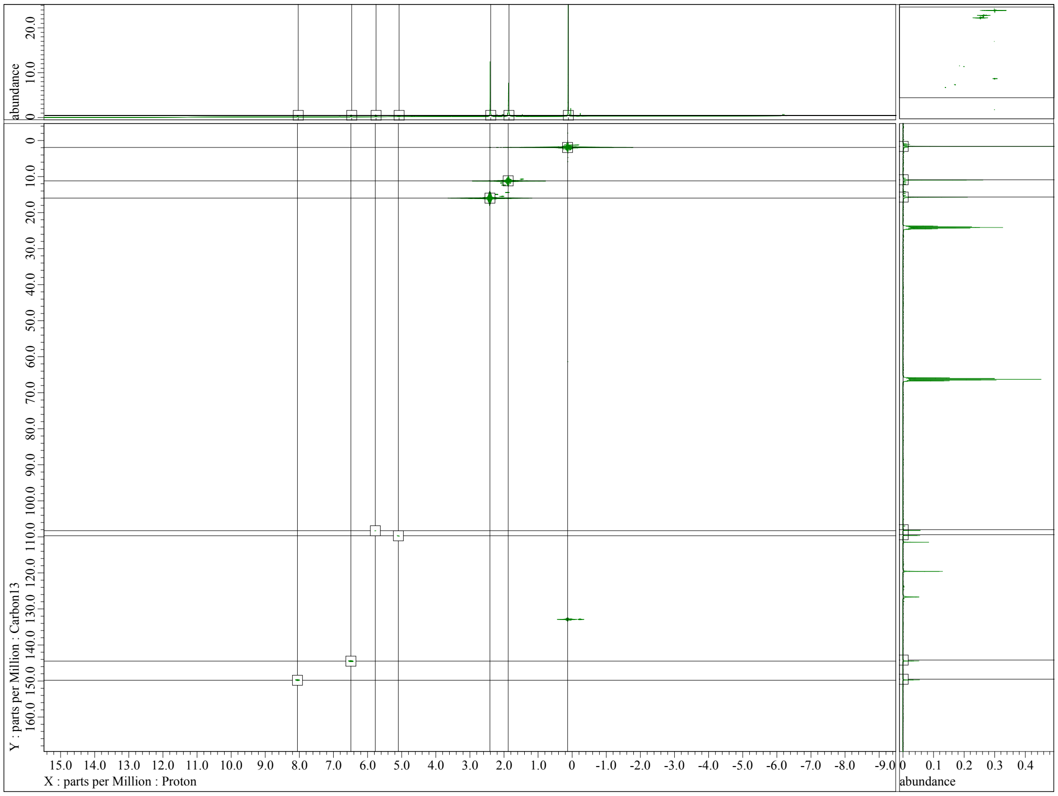

Supplementary Fig. 4 | HMQC NMR spectrum of 2 (THF-d_8, rt)

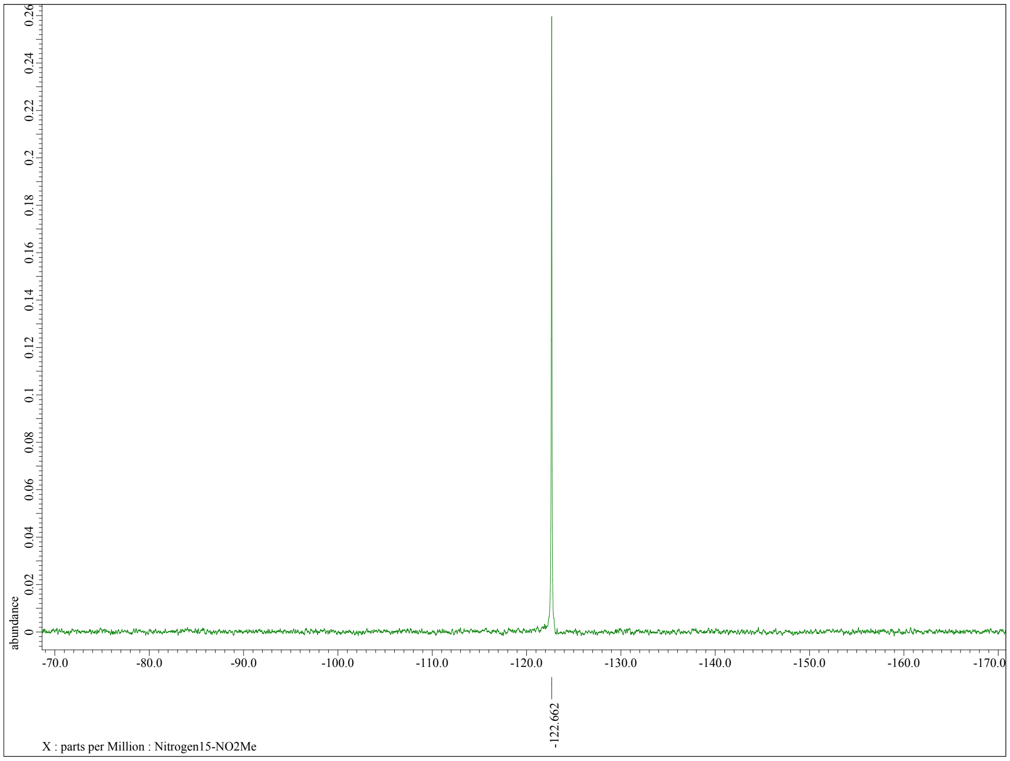

Supplementary Fig. 5 | ^{15}N NMR spectrum of 2-^{15}N (THF-d_8, –60 °C)

$[(C_5Me_4SiMe_3)Ti]_3[\mu\text{-}\eta^2\text{:}\eta^2\text{:}\eta^1\text{:}\eta^1\text{-}(CH)_5](\mu_3\text{-}N)(\mu_2\text{-}H)$ (3). A hexane solution (5.0 mL) of **2** (65 mg, 0.081 mmol) in a 20-mL Schlenk tube equipped with a J. Young valve was stirred at 60 °C for 12 h. After removal of the solvent under vacuum, the resulting residue was dissolved in DME (dimethoxyethane), concentrated and crystallized at –33 °C to give **3** as dark-green crystals (50 mg, 0.062 mmol, 77% yield). Single crystals suitable for X-ray diffraction studies were obtained by recrystallization from DME at –33 °C. The preparation of the ^{15}N-enriched compound $[(C_5Me_4SiMe_3)Ti]_3[\mu\text{-}\eta^2\text{:}\eta^2\text{:}\eta^1\text{:}\eta^1\text{-}(CH)_5](\mu_3\text{-}^{15}N)(\mu_2\text{-}H)$ (**3-^{15}N**) was carried out in the same manner.

^{1}H NMR (C_6D_6, rt): 7.04 (d, $J_{H,H}$ = 10.4 Hz, 2H, C_5H_5), 6.51 (t, , $J_{H,H}$ = 6.8Hz, 1H, C_5H_5), 4.91 (dd, 2H, $J_{H,H}$ = 10.4, 6.8Hz, C_5H_5), 4.76 (s, 1H, μ-H), 2.23, 2.10, 2.07, 2.05, 1.97, 1.55 (s, 6H×6, $C_5Me_4SiMe_3$), 0.16 (s, 18H, $C_5Me_4SiMe_3$), 0.08 (s, 9H, $C_5Me_4SiMe_3$).

^{13}C NMR (C_6D_6, rt): 222.5 (s, C_5H_5), 125.3, 125.2, 124.4, 124.2, 120.5, 118.1 (s, $C_5Me_4SiMe_3$), 112.2 (s, *ipso*-$C_5Me_4SiMe_3$), 107.8 (s, C_5H_5), 105.9 (s, C_5H_5), 16.3, 15.8, 15.3, 13.1, 12.6, 11.7 (s, $C_5Me_4SiMe_3$), 2.3, 2.2 (s, $C_5Me_4SiMe_3$).

^{15}N NMR of **3-^{15}N** (60.81 MHz, THF-d_8, $MeNO_2$, rt): δ 469.3 (s)

Calcd for $C_{41}H_{69}NSi_3Ti_3$: C, 61.26; H, 8.65, N, 1.74. Found: C, 61.55; H, 8.59, N, 1.78.

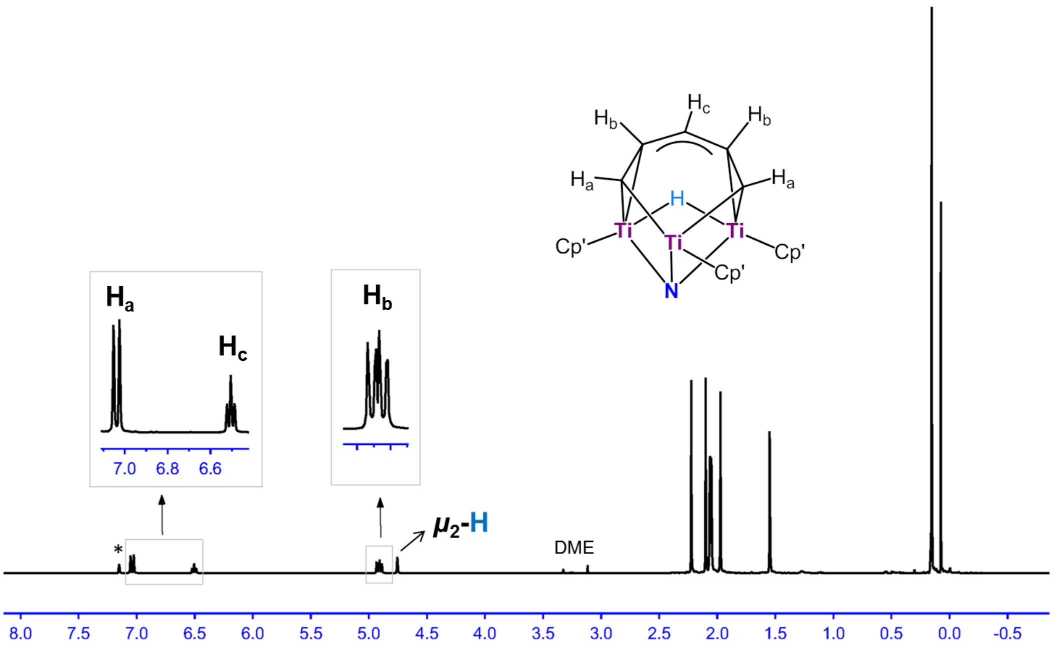

Supplementary Fig. 6 | ^{1}H NMR spectrum of 3 (C_6D_6, rt, * residual signals of C_6D_6)

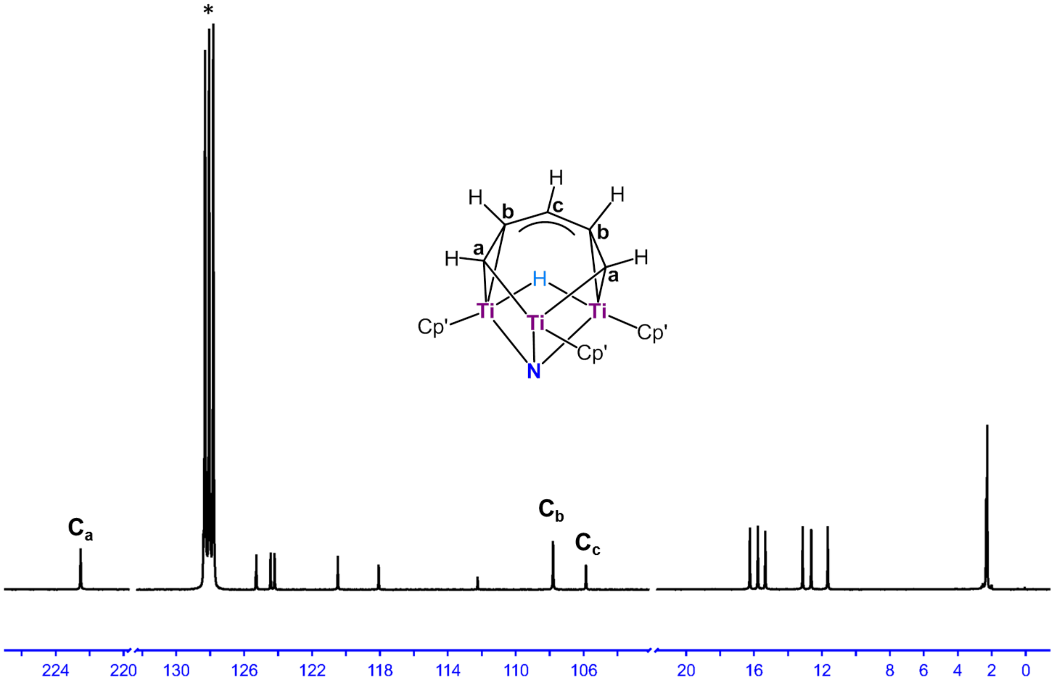

Supplementary Fig. 7 | ^{13}C NMR spectrum of 3 (C_6D_6, rt, * residual signals of C_6D_6)

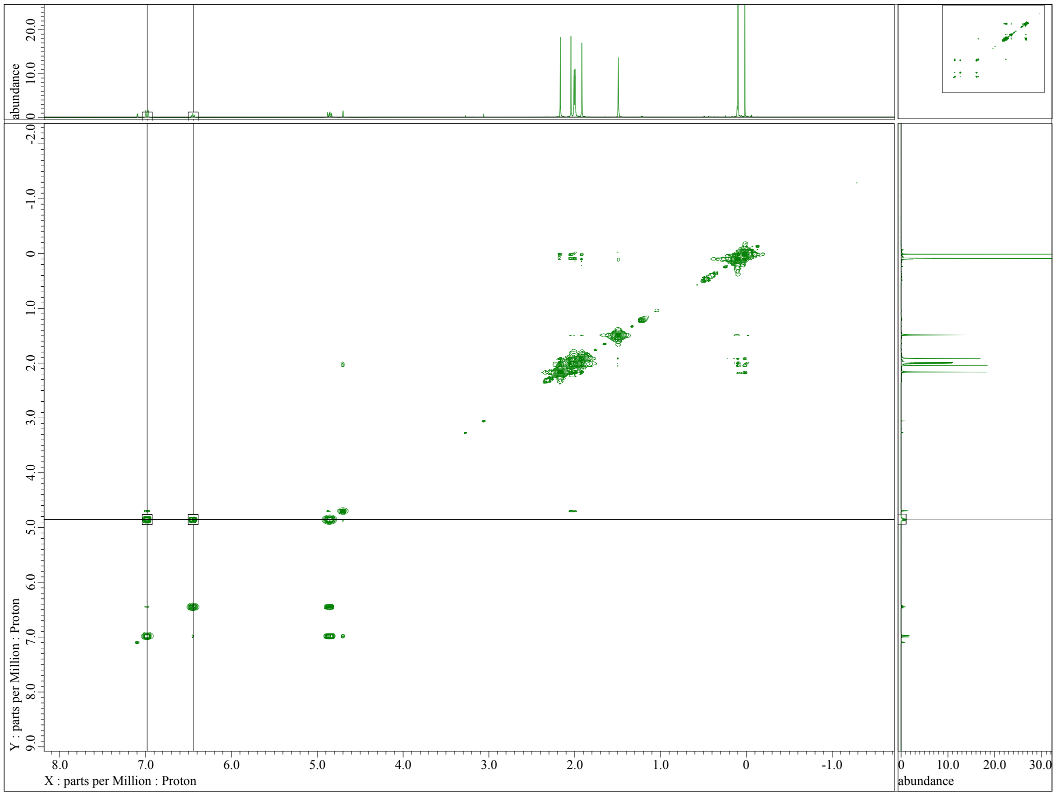

Supplementary Fig. 8 | H-H COSY NMR spectrum of 3 (C_6D_6, rt)

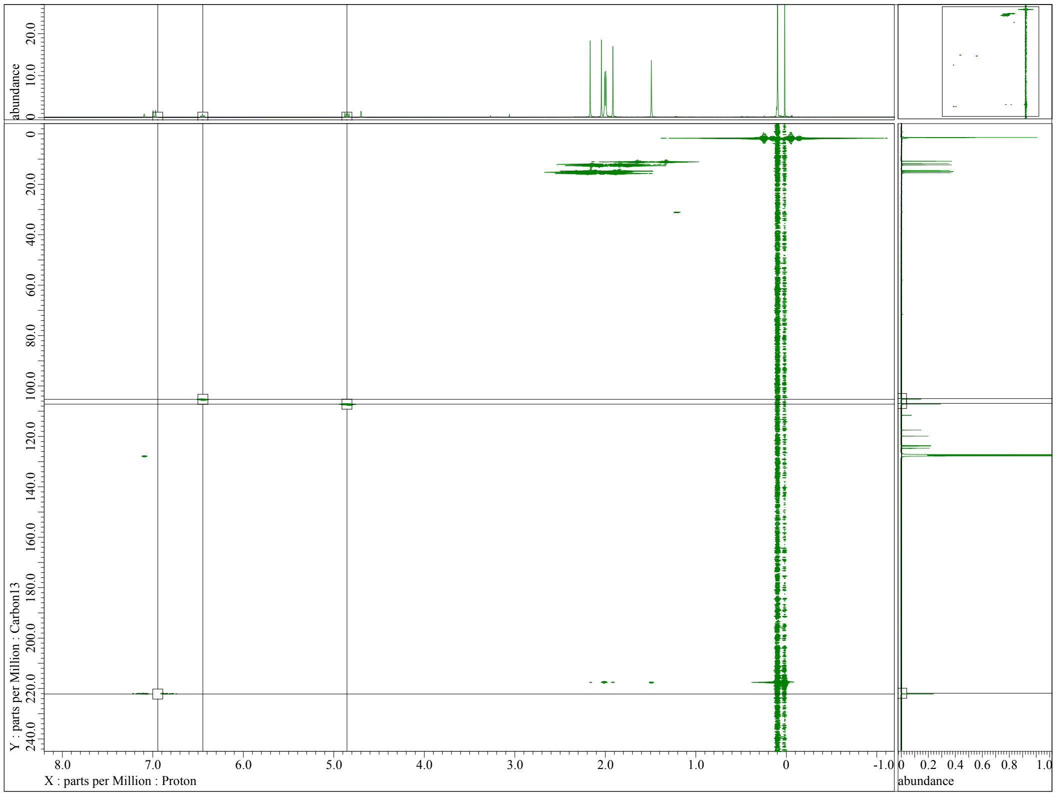

Supplementary Fig. 9 | HMQC NMR spectrum of 3 (C_6D_6, rt)

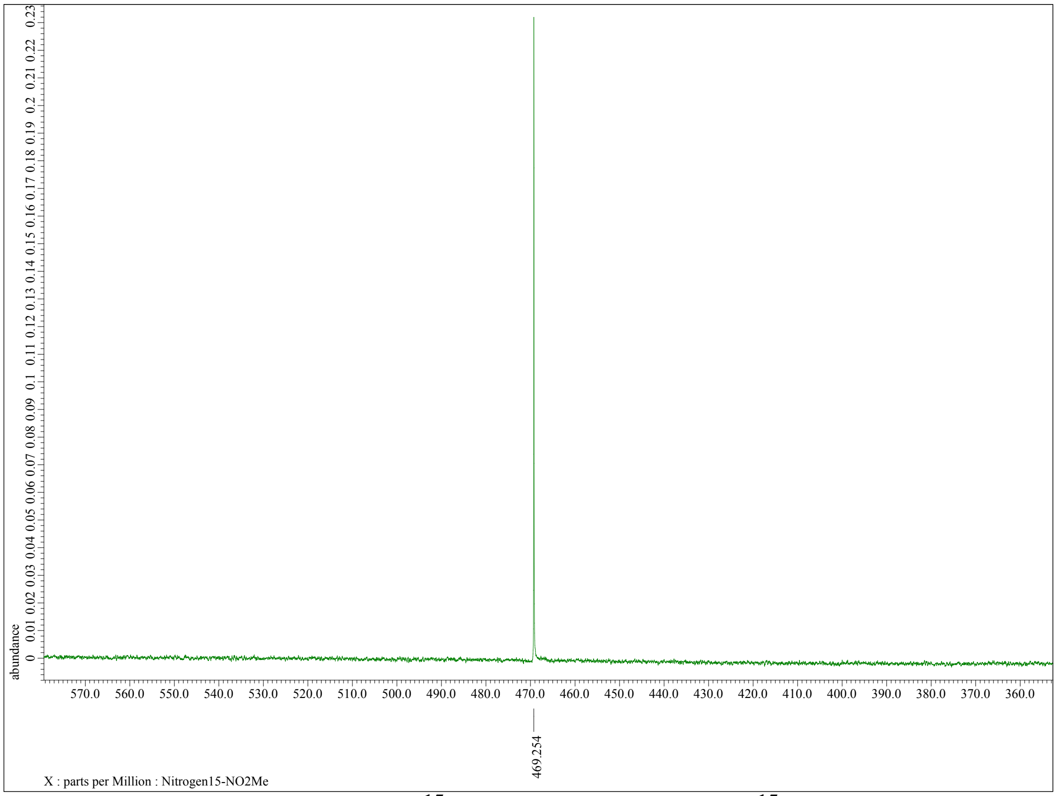

Supplementary Fig. 10 | ^{15}N NMR spectrum of 3-^{15}N (THF-d_8, rt)

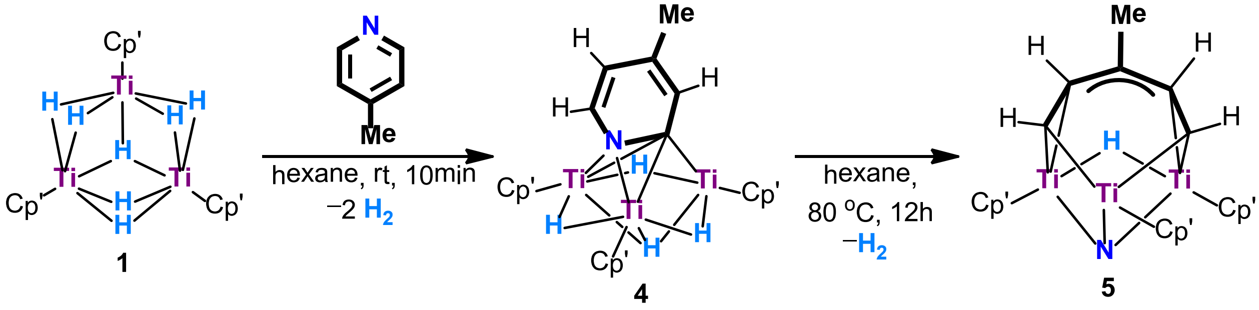

[($C_5Me_4SiMe_3$)Ti]$_3$(μ-η^1:η^2:η^2-C_5H_3MeN)(μ_2-H)$_3$(μ_3-H) (4). In an Ar glovebox, 4-methylpyridine (8 mg, 0.086 mmol) was slowly added to a hexane solution (4.0 mL) of **1** (60 mg, 0.082 mmol). The mixture was stirred at room temperature for 10 min, and an immediate color change from dark green to dark purple was observed. The solution was evaporated under

vacuum and recrystallized in hexane/THF (5:1) at –33 °C to give **4** as dark-purple crystals (60 mg, 0.073 mmol, 89% yield), which were suitable for X-ray diffraction studies.

^{1}H NMR (THF-d_8, −60 °C): 8.13 (d, $J_{H,H}$ = 4.0 Hz, 1H, MeC_5H_4N), 8.11 (d, $J_{H,H}$ = 12.0 Hz, 1H, μ_2-H), 6.28 (s, 1H, MeC_5H_4N), 5.75 (d, $J_{H,H}$ = 4.0 Hz, 1H, MeC_5H_4N), 2.57, 2.49, 2.28, 2.06, 1.95, 1.65 (s, 6H×6, $C_5Me_4SiMe_3$), 1.22 (d, $J_{H,H}$ = 10.0 Hz, 2H, μ_2-H), 1.79 (s, 3H, MeC_5H_4N), 0.20 (s, 18H, $C_5Me_4SiMe_3$), 0.05 (s, 9H, $C_5Me_4SiMe_3$), −6.06 (td, $J_{H,H}$ = 12.0 Hz, 10.0 Hz, 1H, μ_3-H).

^{13}C NMR (THF-d_8, rt): 187.1 (s, MeC_5H_4N), 150.0 (s, MeC_5H_4N), 142.1 (s, MeC_5H_4N), 127.3, 122.6 (s, $C_5Me_4SiMe_3$), 119.5 (s, MeC_5H_4N), 112.3 (s, *ipso*-$C_5Me_4SiMe_3$), 112.0 (s, MeC_5H_4N), 20.2 (s, MeC_5H_4N) 16.8, 12.1 (s, $C_5Me_4SiMe_3$), 2.6 (s, $C_5Me_4SiMe_3$).

Calcd for $C_{42}H_{73}NSi_3Ti_3$: C, 61.53; H, 8.97, N, 1.71. Found: C, 61.75; H, 8.78, N, 1.78.

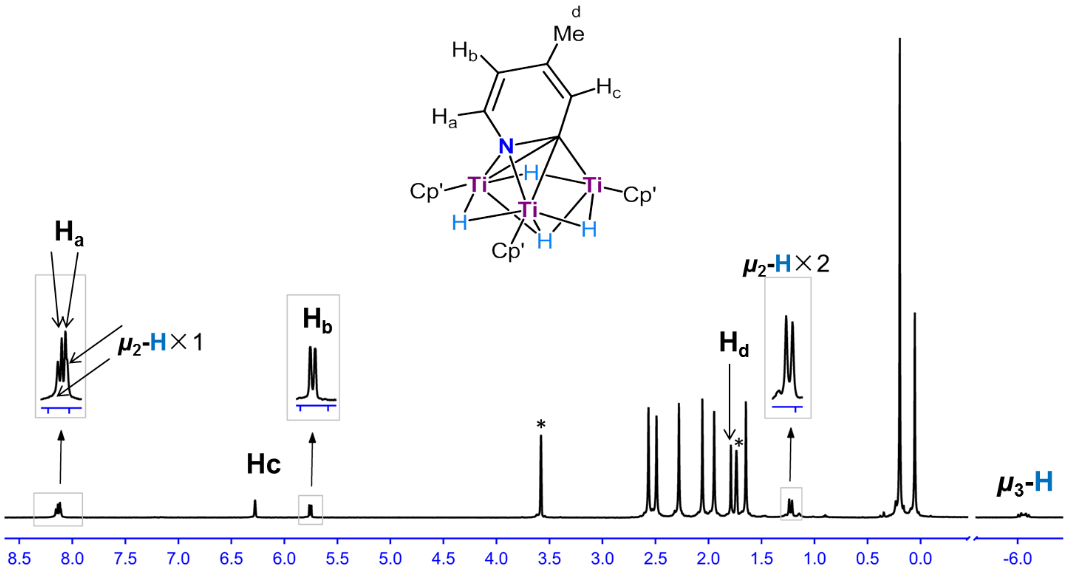

Supplementary Fig. 11 | ^{1}H NMR spectrum of 4 (THF-d_8, −60 °C, * residual signals of THF-d_8)

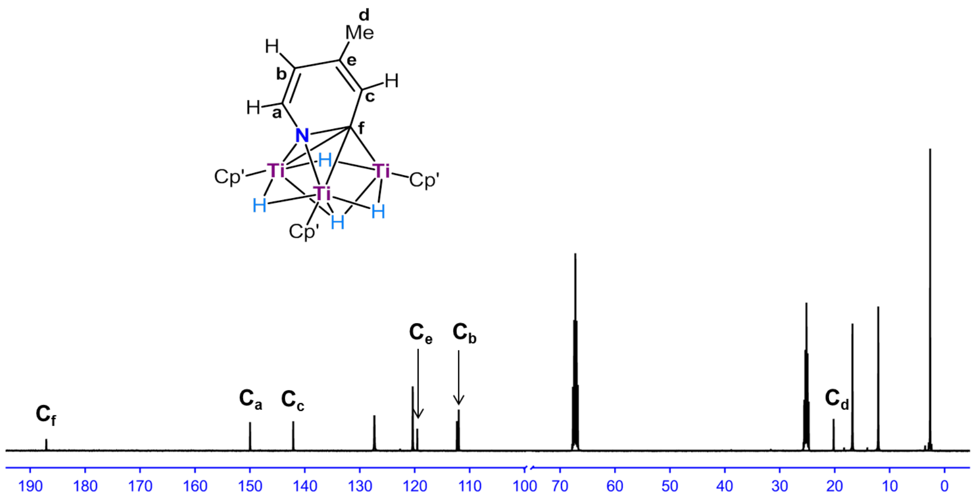

Supplementary Fig. 12 | ^{13}C NMR spectrum of 4 (THF-d_8, rt)

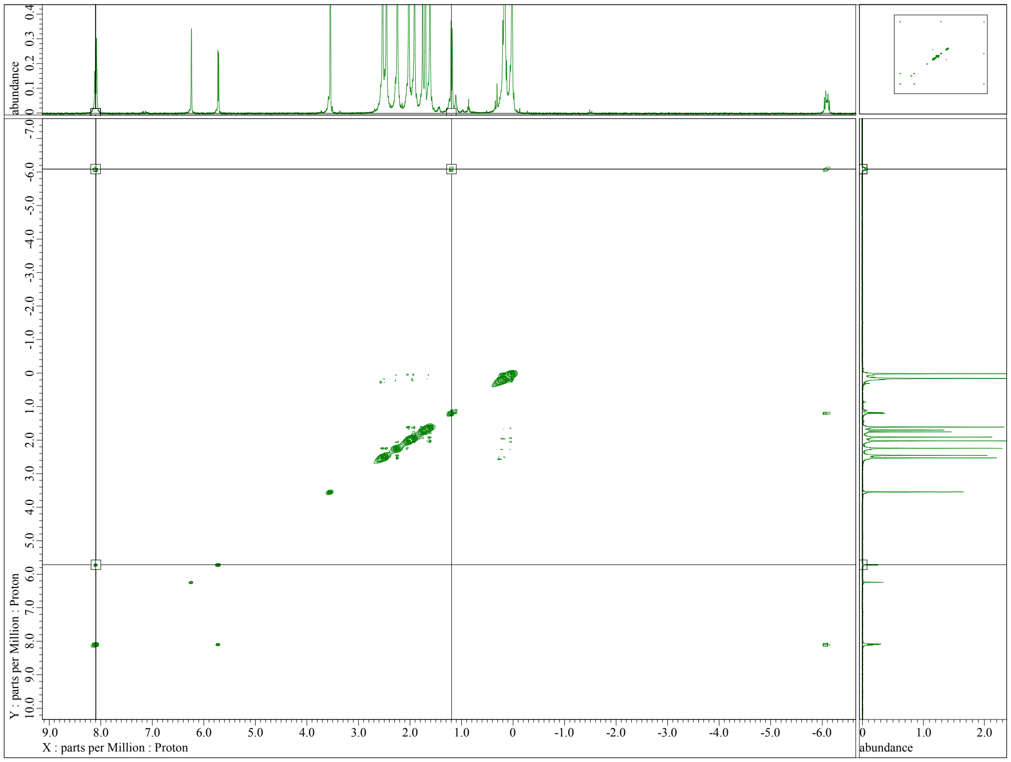

Supplementary Fig. 13 | H-H COSY NMR spectrum of 4 (C_6D_6, rt)

[($C_5Me_4SiMe_3$)Ti]$_3$[μ-η^2:η^2:η^1:η^1-(CH)$_2$C(Me)(CH)$_2$](μ_3-N)(μ_2-H) (5). A hexane solution (5.0 mL) of **4** (50 mg, 0.061 mmol) in a 20-mL Schlenk tube equipped with a J. Young valve was stirred at 80 °C for 12 h. After removal of the solvent under vacuum, the resulting residue was dissolved in DME (dimethoxyethane), concentrated and crystallized at –33 °C to give **5** as dark-green crystals (41 mg, 0.050 mmol, 82% yield). Single crystals suitable for X-ray diffraction studies were obtained by recrystallization from DME at –33 °C.

^{1}H NMR (C_6D_6, rt): 7.02 (d, $J_{H,H}$ = 10.0 Hz, 2H, C_5H_4Me), 5.25 (s, 1H, μ-H), 4.84 (d, $J_{H,H}$ = 10.0 Hz, 2H, C_5*H_4*Me), 2.23 (s, 3H, C_5H_4*Me*), 2.19, 2.10, 2.06, 1.83, 1.55 (s, 6H×4+12H, C_5*Me_4*$SiMe_3$), 0.25 (s, 18H, C_5Me_4Si*Me_3*), 0.08 (s, 9H, C_5Me_4Si*Me_3*).

^{13}C NMR (C_6D_6, rt): 224.1 (s, *C_5*H_4Me), 128.6, 127.4, 125.0, 124.5, 120.0, 118.0 (s, *C_5*Me_4SiMe_3), 125.9 (s, *C_5*H_4Me), 113.3 (s, *C_5*H_4Me) 112.0 (s, *ipso*-*C_5*Me_4SiMe_3), 26.8 (s, C_5H_4*Me*), 16.3, 15.8, 15.3, 12.8, 12.7, 11.6 (s, C_5*Me_4*$SiMe_3$), 2.7, 2.4 (s, C_5Me_4Si*Me_3*).

Calcd for $C_{42}H_{71}NSi_3Ti_3$: C, 61.68; H, 8.75, N, 1.71. Found: C, 61.97; H, 8.68, N, 1.84.

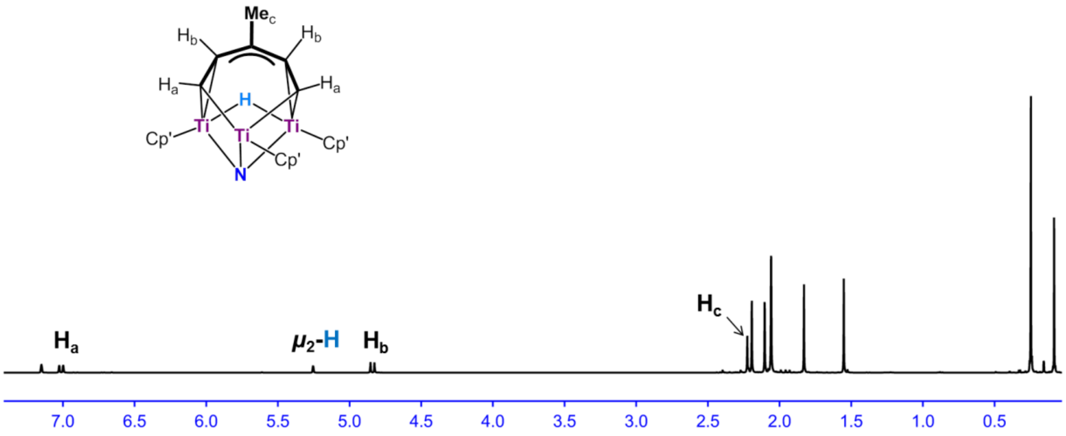

Supplementary Fig. 14 | ^{1}H NMR spectrum of 5 (C_6D_6, rt)

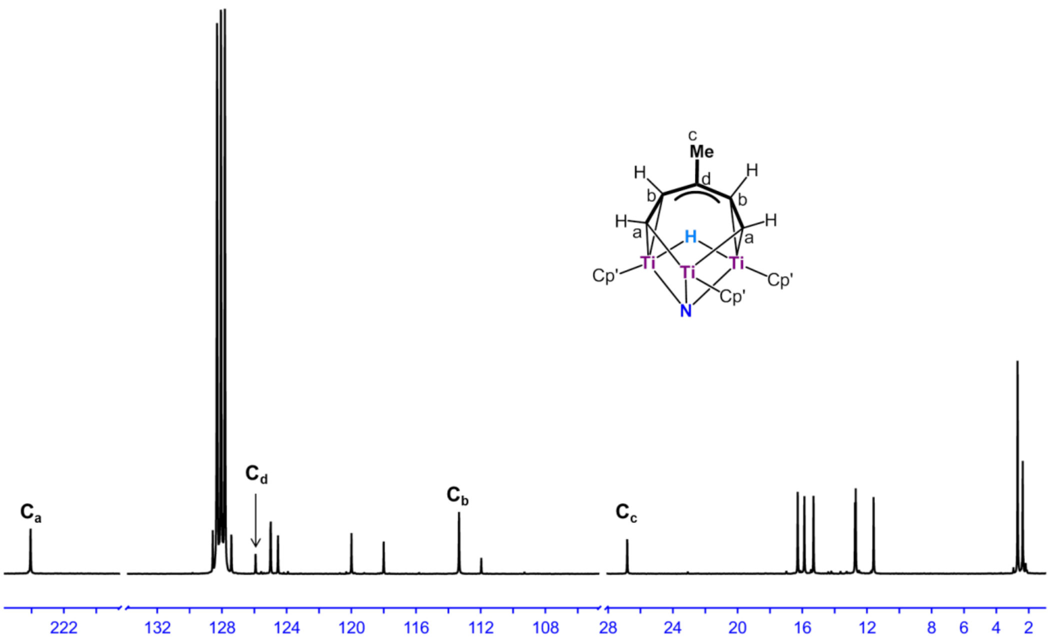

Supplementary Fig. 15 | ^{13}C NMR spectrum of 5 (C_6D_6, rt)

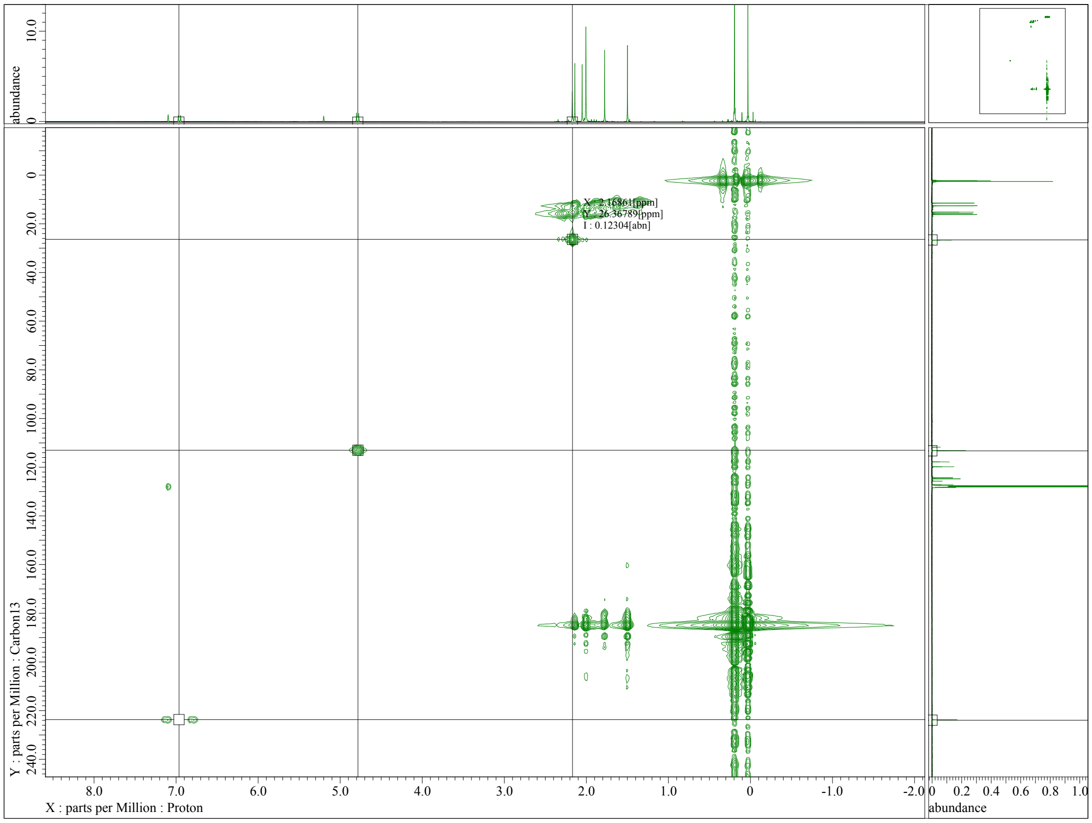

Supplementary Fig. 16 | HMQC NMR spectrum of 5 (C_6D_6, rt)

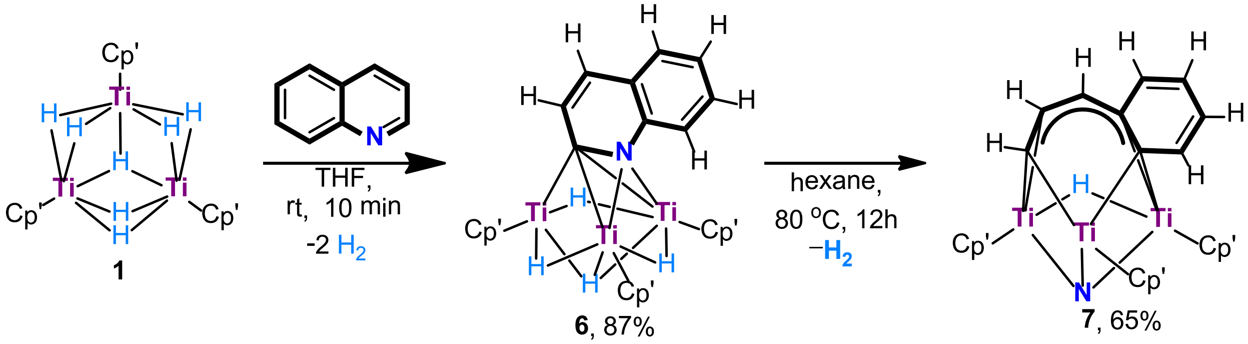

[($C_5Me_4SiMe_3$)Ti]$_3$(μ-η^1:η^2:η^2-C_9H_6N)(μ_2-H)$_3$(μ_3-H) (6). In an Ar glovebox, quinoline (18.6 mg, 0.143 mmol) was slowly added to a THF solution (5.0 mL) of complex **1** (70.0 mg, 0.096 mmol). The mixture was stirred at room temperature for 10 min, and an immediate color change from dark green to dark red was observed. After removal of the solvent under vacuum, the resulting residue was dissolved in hexane, concentrated and crystallized at –33 °C to give **6** as dark-red crystals (71 mg, 0.083 mmol, 87% yield).

^{1}H NMR (THF-d_8, rt): 8.61 (d, $J_{H,H}$ = 20.0 Hz, 1H, μ_2-H), 7.86 (d, $J_{H,H}$ = 8.0 Hz, 1H, C_9H_6N), 7.86 (m, $J_{H,H}$ = 8.0 Hz, 2.0 Hz, 1H, $C_9\mathit{H}_6N$), 6.95 (dd, $J_{H,H}$ = 2.0 Hz, 4.0 Hz, 1H, $C_9\mathit{H}_6N$), 6.94 (d, $J_{H,H}$ = 4.0 Hz, 1H, $C_9\mathit{H}_6N$), 6.18 (d, $J_{H,H}$ = 12 Hz, 1H, $C_9\mathit{H}_6N$), 5.34 (d, $J_{H,H}$ = 12 Hz, 1H, $C_9\mathit{H}_6N$), 2.57, 2.55, 2.33, 2.26, 1.95, 0.91 (s, 6H×6, $C_5\mathit{Me}_4SiMe_4$), 1.81 (d, $J_{H,H}$ = 8.0 Hz, 2H, μ_2-H), 0.21 (s, 18H, $C_5Me_4Si\mathit{Me}_3$), 0.08 (s, 9H, $C_5Me_4Si\mathit{Me}_3$). -6.35 (td, $J_{H,H}$ = 20.0 Hz, 8.0 Hz, 1H, μ_3-H).

^{13}C NMR (THF-d_8, rt): 186.7, 156.7, 139.8, 126.5, 126.4, 125.75, 124.3, 113.4, 110.7 (s, C_9H_6N), 131.0, 130.2, 128.2, 122.0, 121.8, 120.6, (s, $\mathit{C}_5Me_4SiMe_3$), 113.3 (s, *ipso*-$\mathit{C}_5Me_4SiMe_3$), 17.9, 17.8, 17.4, 13.7, 12.1, 10.0(s, $C_5\mathit{Me}_4SiMe_3$), 3.0, 2.9 (s, $C_5Me_4Si\mathit{Me}_3$).

Calcd for $C_{45}H_{73}NSi_3Ti_3$: C, 63.15; H, 8.60, N, 1.64. Found: C, 63.29; H, 8.60, N, 1.74.

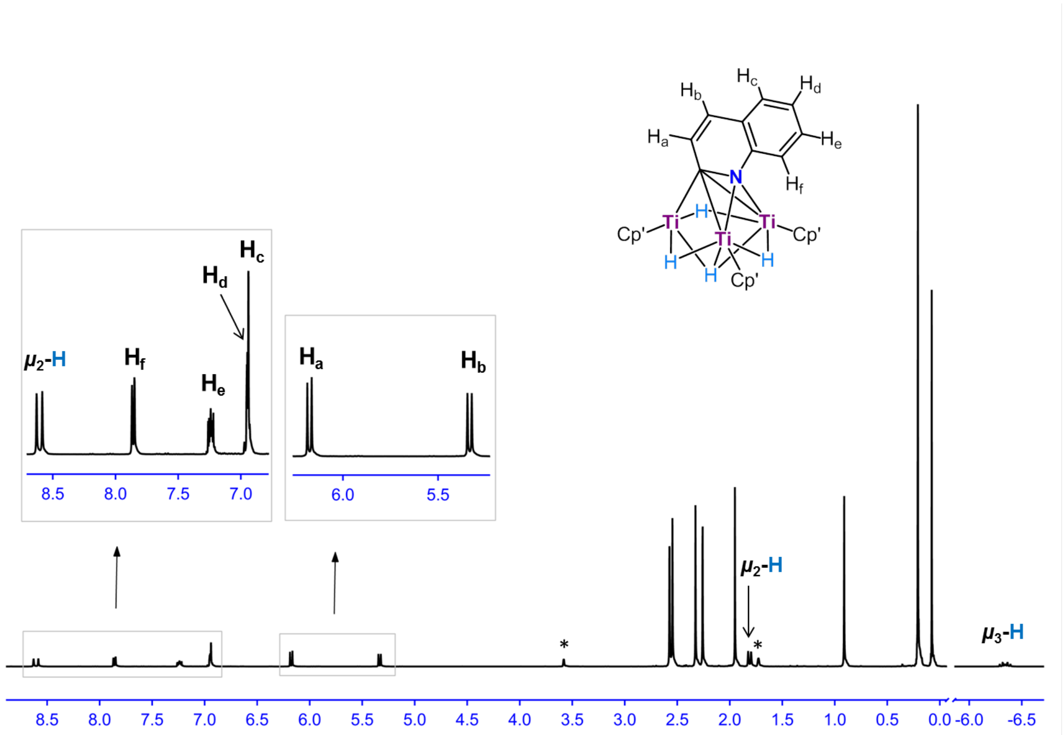

Supplementary Fig. 17 | ^{1}H NMR spectrum of 6 (THF-d_8, rt, * residual signals of THF-d_8)

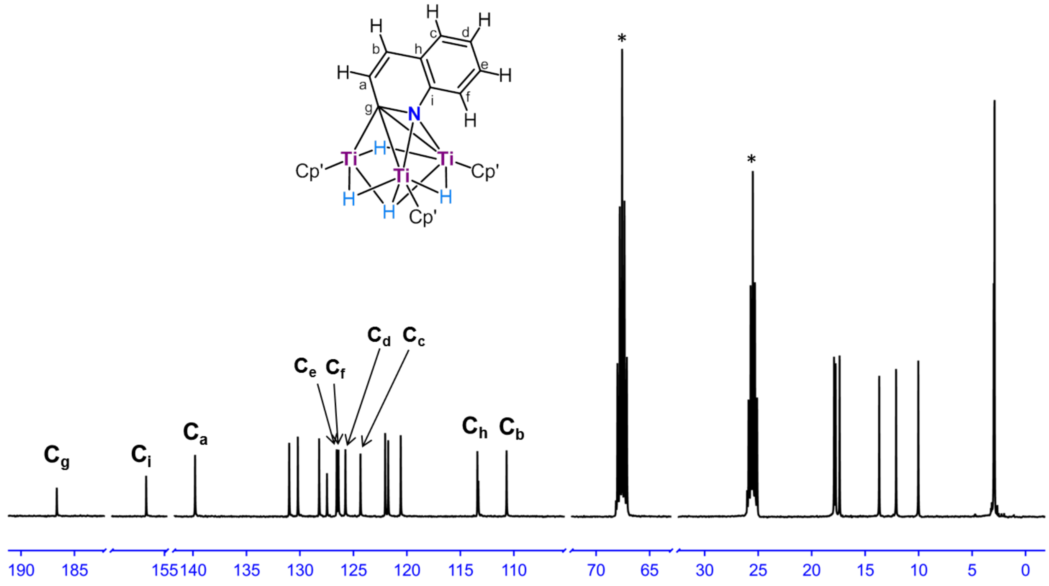

Supplementary Fig. 18 | ^{13}C NMR spectrum of 6 (THF-d_8, rt, * residual signals of THF-d_8)

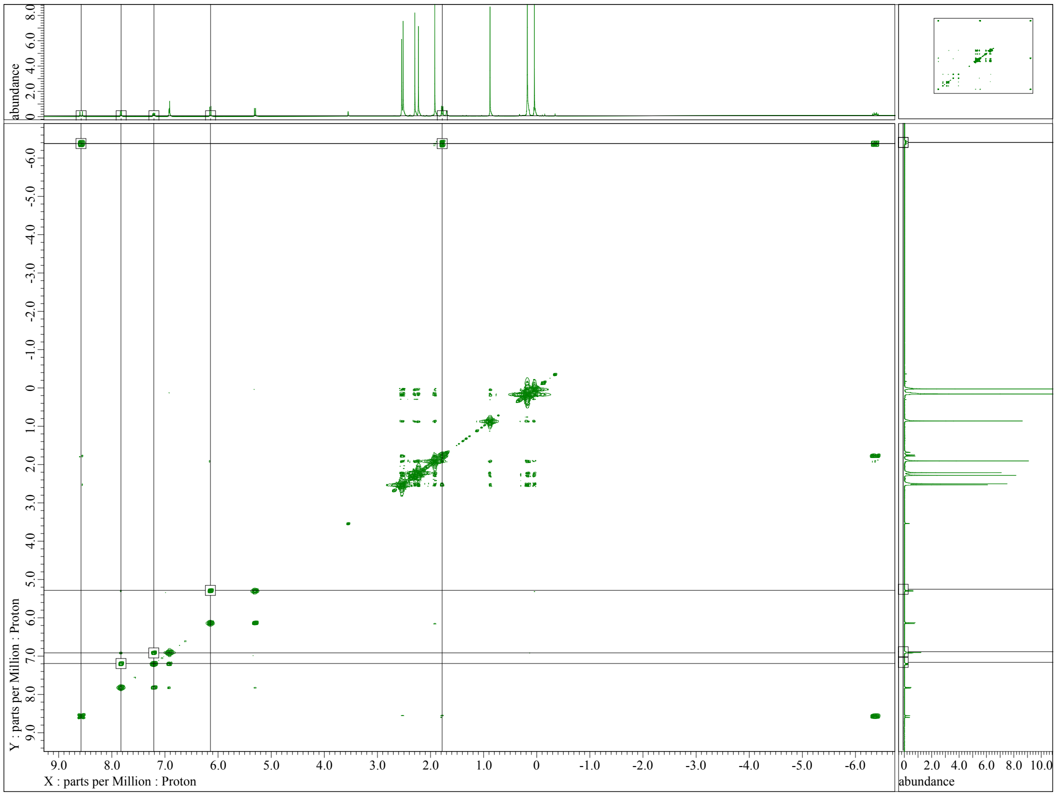

Supplementary Fig. 19 | H-H COSY NMR spectrum of 6 (THF-d_8, rt)

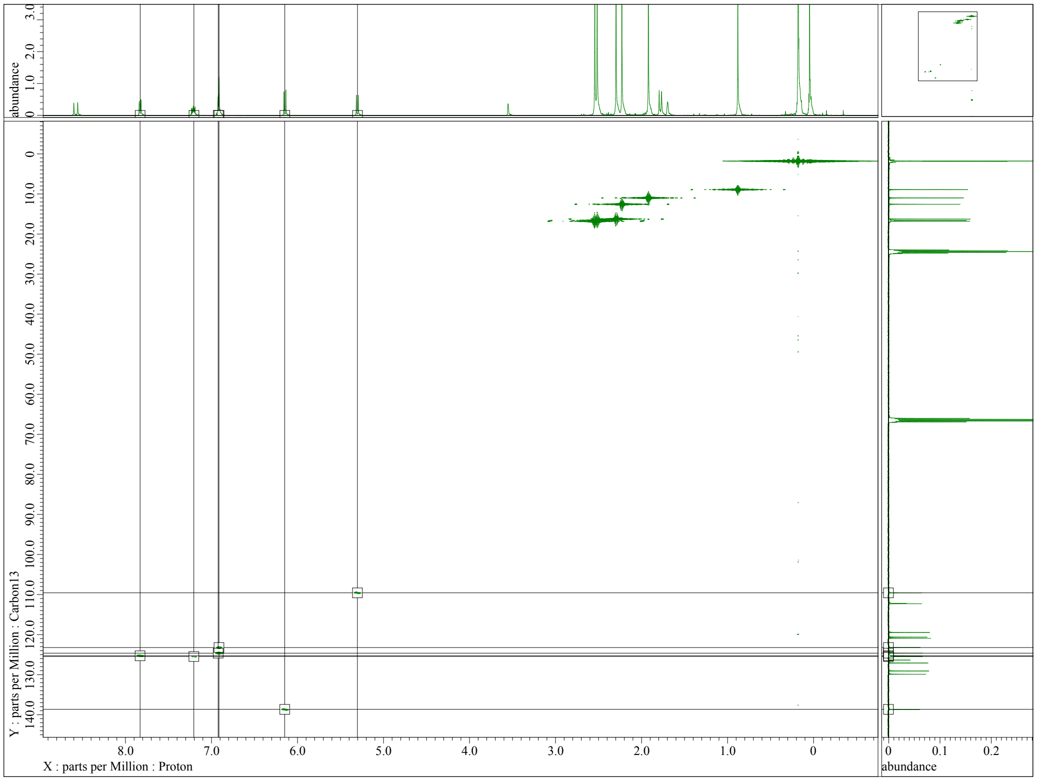

Supplementary Fig. 20 | HMQC NMR spectrum of 6 (THF-d_8, rt)

$[(C_5Me_4SiMe_3)Ti]_3(\mu\text{-}\eta^2{:}\eta^2{:}\eta^1{:}\eta^1\text{-}CHCHCHC_6H_4)(\mu_3\text{-}N)(\mu_2\text{-}H)$ (**7**) A hexane solution (5.0 mL) of **6** (40 mg, 0.047 mmol) in a 20-mL Schlenk tube equipped with a J. Young valve was stirred at 80 °C for 12 h. After removal of the solvent under vacuum, the resulting residue was dissolved in a diethyl ether/hexane mixed solvent (2/1), concentrated and crystallized at –33 °C to give **7** as dark-brown crystals (26 mg, 0.030 mmol, 65% yield), which were suitable for X-ray diffraction studies.

^{1}H NMR (THF-d_8, rt): 7.59 (d, $J_{H,H}$ = 8.0 Hz, 1H, C_9H_7), 7.08 (d, $J_{H,H}$ = 8.0 Hz, 1H, C_9H_7), 7.03 (d, $J_{H,H}$ = 10.8 Hz, 1H, C_9H_7), 6.77 (t, $J_{H,H}$ = 8.0 Hz, 1H, C_9H_7), 6.64 (d, $J_{H,H}$ = 8.0 Hz, 1H, C_9H_7), 5.92 (t, $J_{H,H}$ = 8.0 Hz, 1H, C_9H_7), 5.37 (dd, $J_{H,H}$ = 8.0, 10.8 Hz, 1H, C_9H_7), 2.68 (s, 1H, μ_2-H), 2.33, 2.26, 2.01, 1.99, 1.98, 1.95, 1.92, 1.82, 1.81, 1.80, 1.42, 1.35 (s, 3H×12, $C_5Me_4SiMe_4$), 0.17, -0.12, -0.32 (s, 9H×3, $C_5Me_4SiMe_3$).

^{13}C NMR (THF-d_8, rt): 231.3, 205.4, 139.4, 132.1, 128.3, 114.6, 113.9, 112.2, 82.1 (s, C_9H_7), 131.4, 129.4, 128.9, 127.5, 127.2, 126.8, 124.9, 123.2, 123.1, 122.0, 120.2, 118.0 (s, $C_5Me_4SiMe_3$), 114.3 (s, *ipso*-$C_5Me_4SiMe_3$), 18.1, 16.8, 16.7, 16.3, 15.4, 14.1, 14.0, 13.4, 13.3, 12.8, 12.4, 12.3 (s, $C_5Me_4SiMe_3$), 3.0, 2.9 (s, $C_5Me_4SiMe_3$).

Calcd for $C_{45}H_{71}NSi_3Ti_3$: C, 63.29; H, 8.38, N, 1.64. Found: C, 63.64; H, 8.34, N, 1.84.

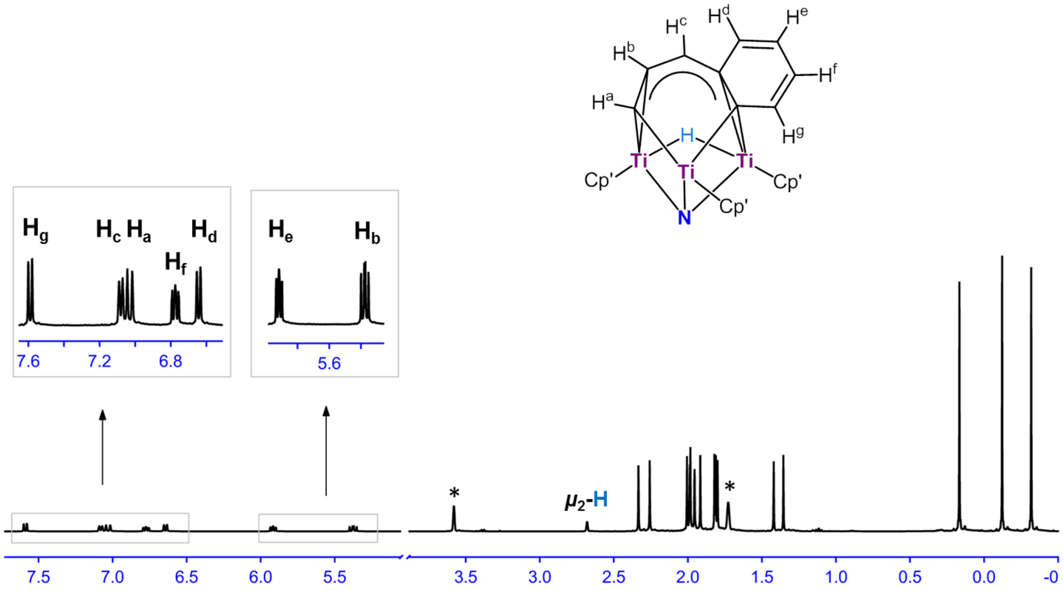

Supplementary Fig. 21 | ^{1}H NMR spectrum of 7 (THF-d_8, rt, * residual signals of THF-d_8)

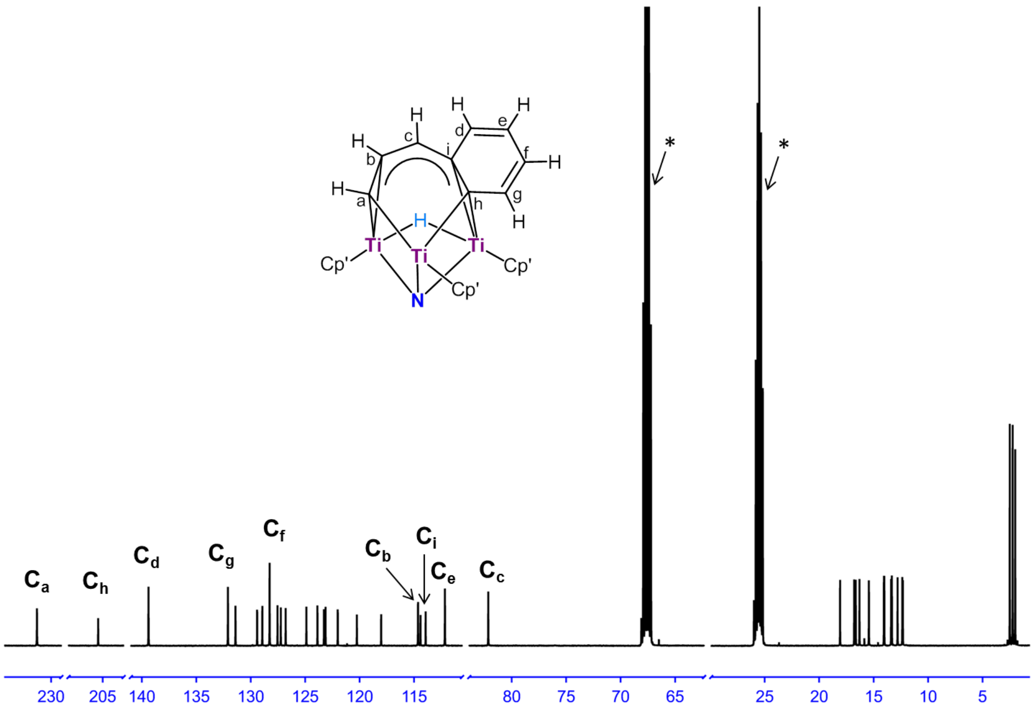

Supplementary Fig. 22 | ^{13}C NMR spectrum of 7 (THF-*d*$_8$, rt, * residual signals of THF-*d*$_8$)

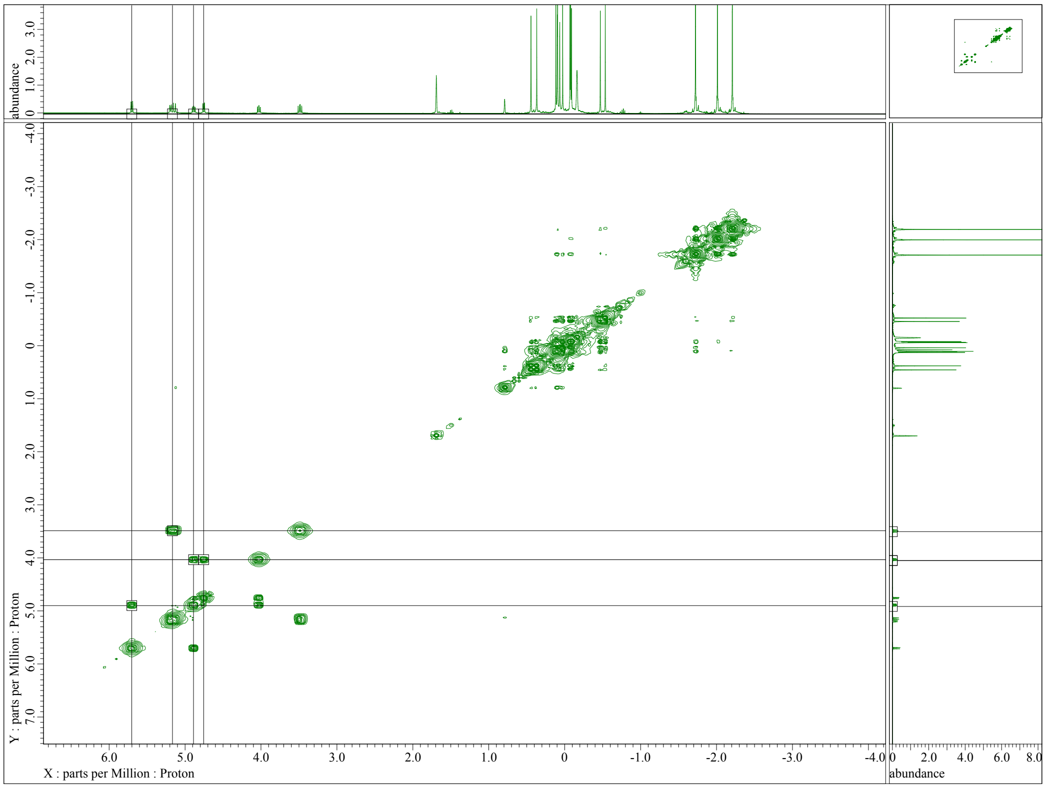

Supplementary Fig. 23 | H-H COSY NMR spectrum of 7 (THF-*d*$_8$, rt)

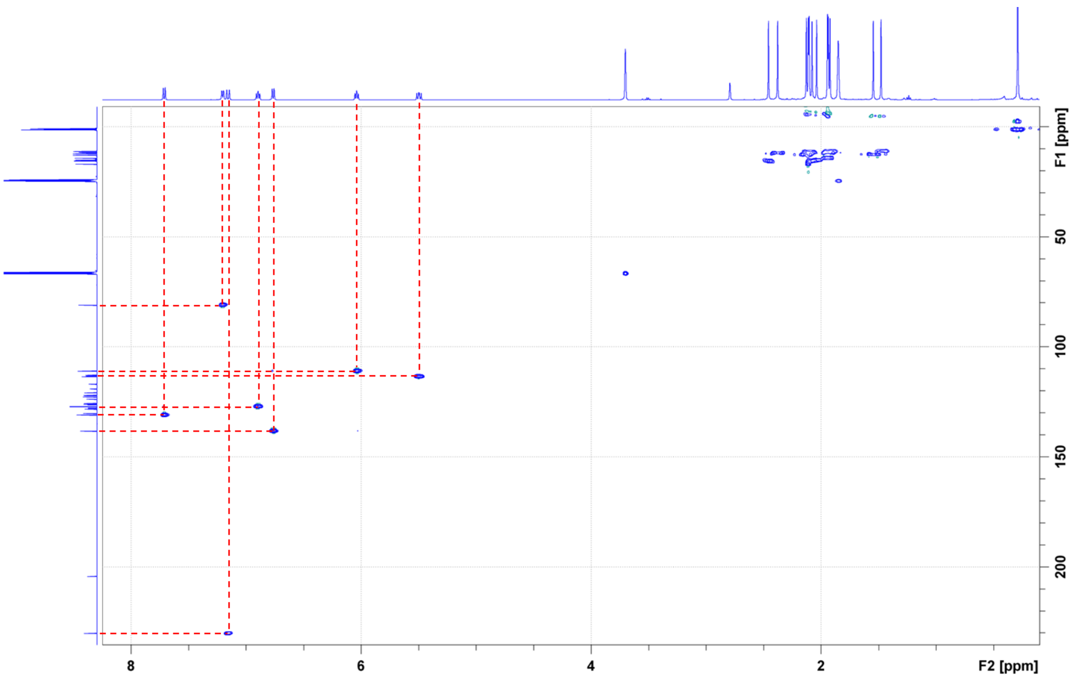

Supplementary Fig. 24 | HMQC NMR spectrum of 7 (THF-d_8, rt)

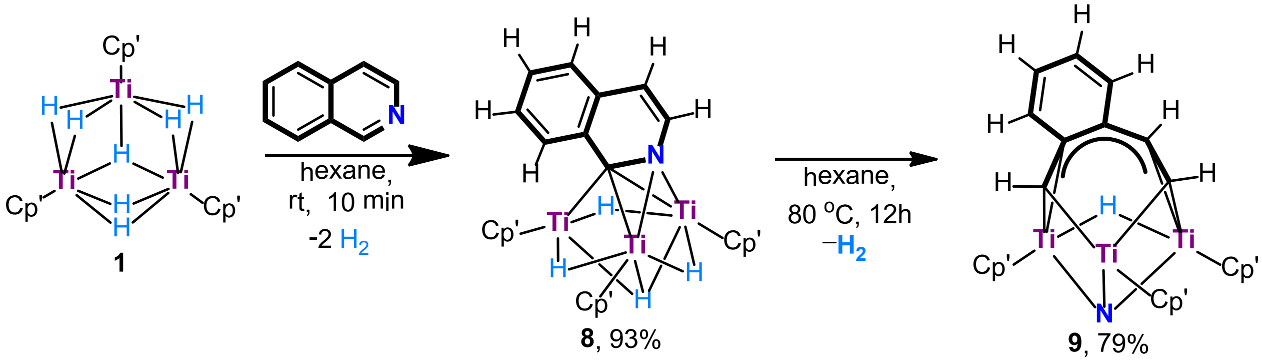

$[(C_5Me_4SiMe_3)Ti]_3(\mu\text{-}\eta^1\text{:}\eta^2\text{:}\eta^2\text{-}iso\text{-}C_9H_6N)(\mu_2\text{-H})_3(\mu_3\text{-H})$ (8). In an Ar glovebox, isoquinoline (13.0 mg, 0.100 mmol) was slowly added to a hexane solution (4.0 mL) of **1** (70.0 mg, 0.096 mmol), and the mixture was stirred at room temperature for 10 min. After removal of the solvent under vacuum, the resulting dark red residue was dissolved in hexane/THF (5:1), concentrated and crystallized at –33 °C to give **8** as dark-red crystals (76 mg, 0.089 mmol, 93% yield), which were suitable for X-ray studies.

^{1}H NMR (THF-d_8, rt): 7.84 (d, $J_{H,H}$ = 8.0 Hz, 1H, C_9H_6N), 6.94 (d, $J_{H,H}$ = 6.0 Hz, 1H, C_9H_6N), 6.87 (t, $J_{H,H}$ = 6.0 Hz, 1H, C_9H_6N), 6.73 (t, $J_{H,H}$ = 6.0 Hz, 1H, C_9H_6N), 6.20 (d, $J_{H,H}$ = 6.0 Hz, 1H, C_9H_6N), 6.06 (d, $J_{H,H}$ = 8.0 Hz, 1H, C_9H_6N), 3.46 (d, $J_{H,H}$ = 12.0 Hz, 3H, μ_2-H), 2.43, 1.97 (s, 18H×2, $C_5Me_4SiMe_3$), 0.09 (s, 27H, $C_5Me_4SiMe_3$), -7.36 (q, $J_{H,H}$ = 12.0 Hz, 1H, μ_3-H).

^{13}C NMR (THF-d_8, rt): 184.5, 146.3, 145.7, 134.4, 127.8, 126.2, 125.5, 124.7, 111.0 (s, C_9H_6N), 127.7, 121.7 (s, $C_5Me_4SiMe_3$), 112.7 (s, *ipso*-$C_5Me_4SiMe_3$), 16.8, 12.6 (s, $C_5Me_4SiMe_3$), 2.6 (s, $C_5Me_4SiMe_3$).

Calcd for $C_{45}H_{73}NSi_3Ti_3$: C, 63.15; H, 8.60, N, 1.64. Found: C, 62.32; H, 8.53, N, 1.99.

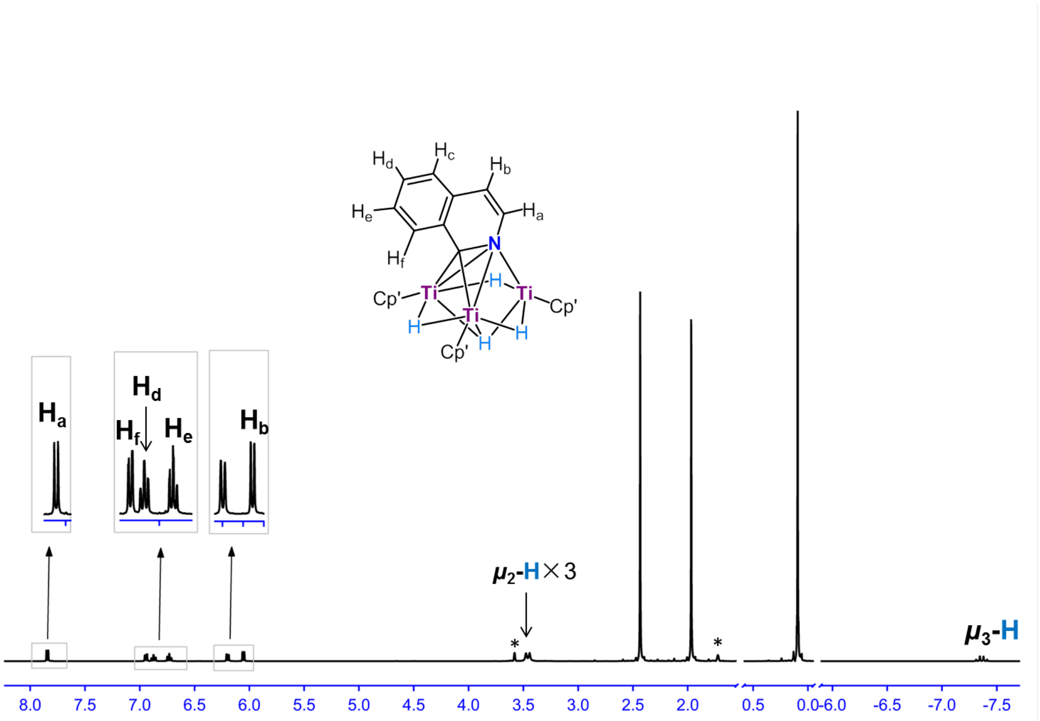

Supplementary Fig. 25 | ^{1}H NMR spectrum of 8 (THF-d_8, rt, * residual signals of THF-d_8)

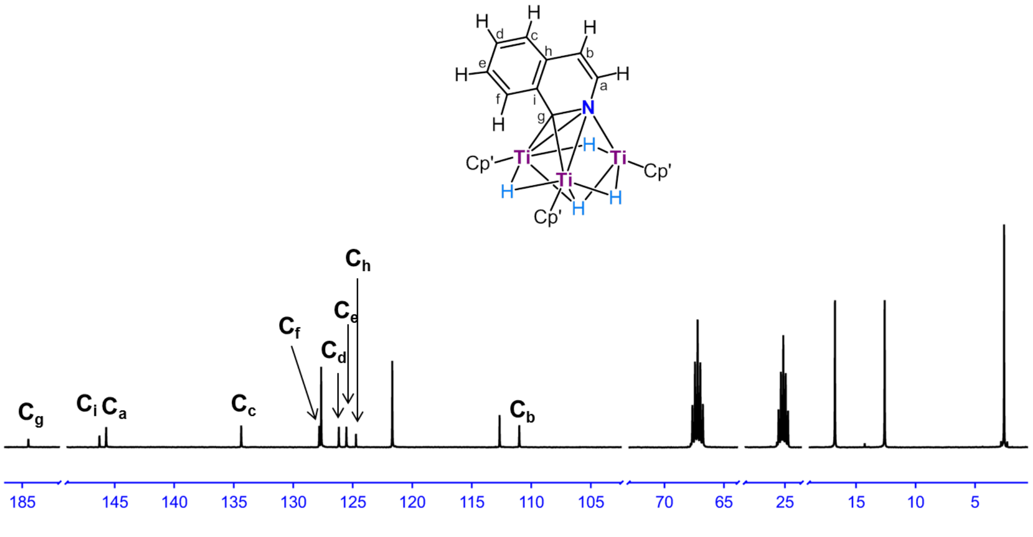

Supplementary Fig. 26 | ^{13}C NMR spectrum of 8 (THF-d_8, rt)

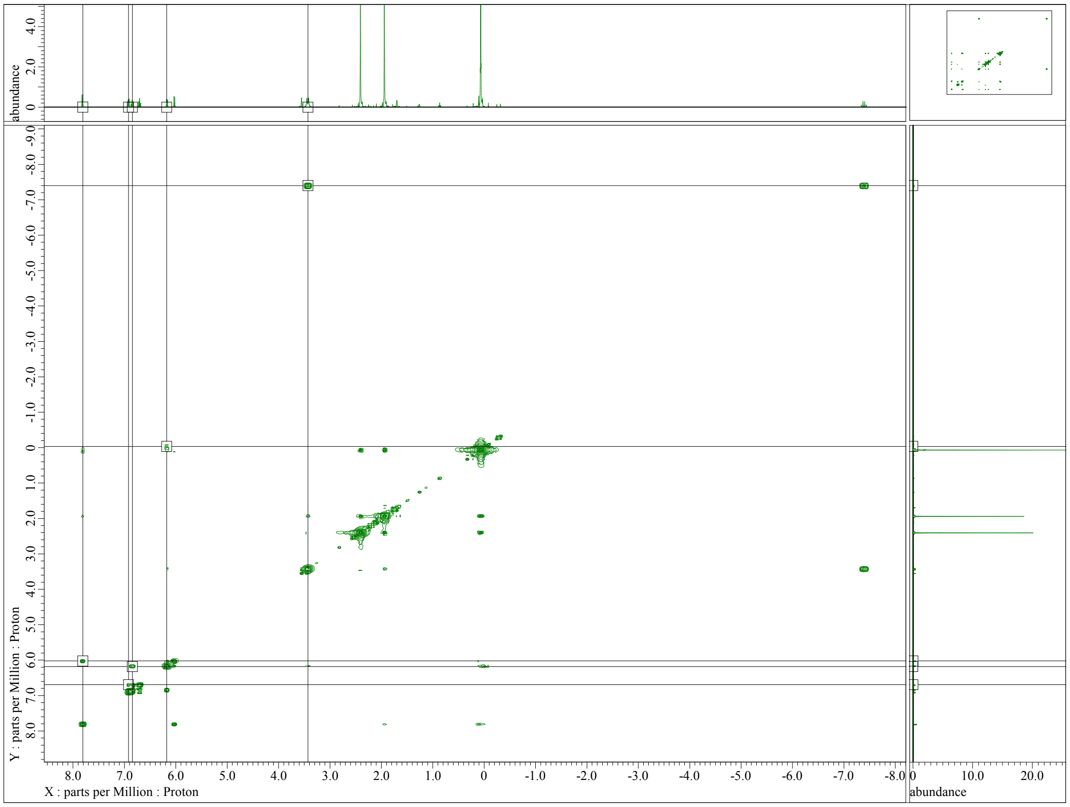

Supplementary Fig. 27 | H-H COSY NMR spectrum of 8 (THF-d_8, rt)

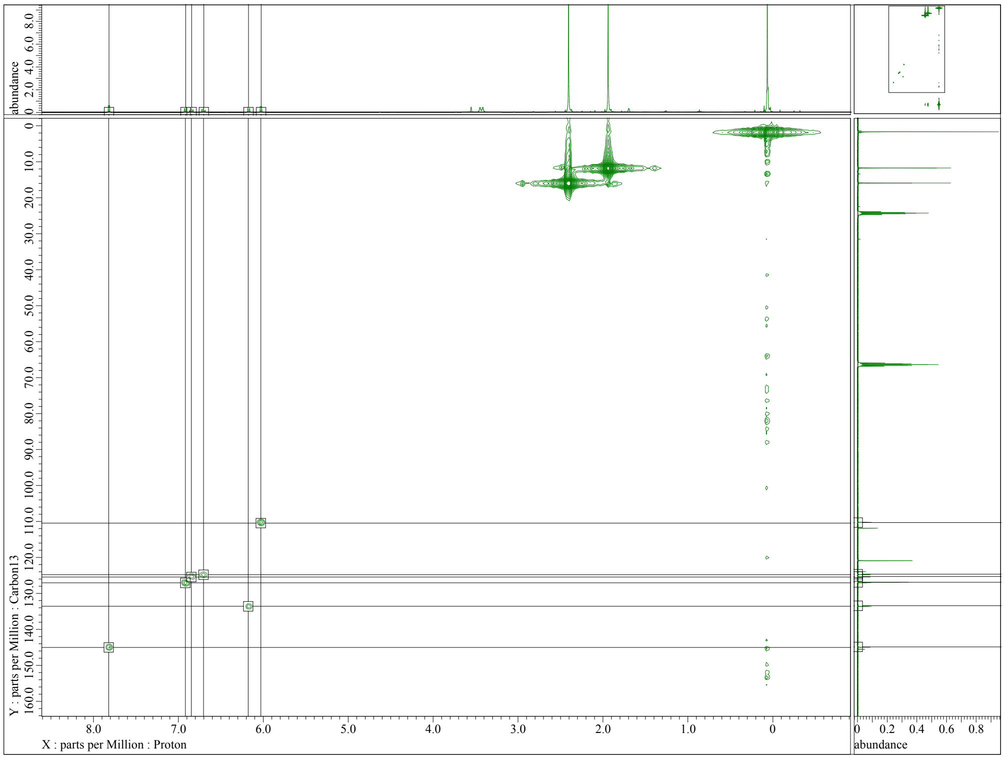

Supplementary Fig. 28 | HMQC NMR spectrum of 8 (THF-d_8, rt)

[($C_5Me_4SiMe_3$)Ti]$_3$(μ-η^2:η^2:η^1:η^1-CHCHC$_6$H$_4$CH)(μ_3-N)(μ_2-H) (9). A hexane solution (5.0 mL) of **8** (50 mg, 0.058 mmol) in a 20-mL Schlenk tube equipped with a J. Young valve was stirred at 80 °C for 12 h. After removal of the solvent under vacuum, the resulting residue was dissolved in DME, concentrated and crystallized at –33 °C to give **9** as dark-brown crystals (39 mg, 0.046 mmol, 79% yield), which were suitable for X-ray diffraction studies.

^{1}H NMR (C_6D_6, rt): 7.91 (s, 1H, C$_9$*H*$_7$), 7.08 (d, $J_{H,H}$ = 6.8 Hz, 1H, C$_9$*H*$_7$), 6.95 (d, $J_{H,H}$ = 12.0 Hz, 1H, C$_9$*H*$_7$), 6.89 (s, 1H, μ_2-H), 6.85 (t, $J_{H,H}$ = 6.8 Hz, 1H, C$_9$*H*$_7$), 6.72 (t, $J_{H,H}$ = 6.8 Hz, 1H, C$_9$*H*$_7$), 6.49 (d, $J_{H,H}$ = 6.8 Hz, 1H, C$_9$*H*$_7$), 4.65 (d, $J_{H,H}$ = 12.0 Hz, 1H, C$_9$*H*$_7$), 2.46, 2.36, 2.15, 2.14, 2.13, 2.06, 2.04, 1.91, 1.68, 1.66, 1.56 (s, 3H×10+6H, C$_5$*Me*$_4$SiMe$_3$), 0.23, 0.05, 0.10 (s, 9H×3, C$_5$Me$_4$Si*Me*$_3$).

^{13}C NMR (C_6D_6, rt): 232.5, 227.3, 140.3, 134.3, 128.3, 129.8, 126.5, 125.1, 118.57, 118.2 (s, *C*$_9$H$_7$), 131.0, 130.3, 128.7, 127.2, 126.4, 125.4, 125.4, 125.1, 120.8, 120.7, 119.4, 118.8 (s, *C*$_5$Me$_4$SiMe$_3$), 112.2 (s, *ipso*-*C*$_5$Me$_4$SiMe$_3$), 17.2, 16.8, 16.2, 16.1, 15.4, 15.3, 14.5, 14.0, 12.4, 12.1, 11.8, 11.6 (s, C$_5$*Me*$_4$SiMe$_3$), 2.8, 2.2, 2.1 (s, C$_5$Me$_4$Si*Me*$_3$).

Calcd for $C_{45}H_{71}NSi_3Ti_3$: C, 63.29; H, 8.38, N, 1.64. Found: C, 63.52; H, 8.27, N, 1.86.

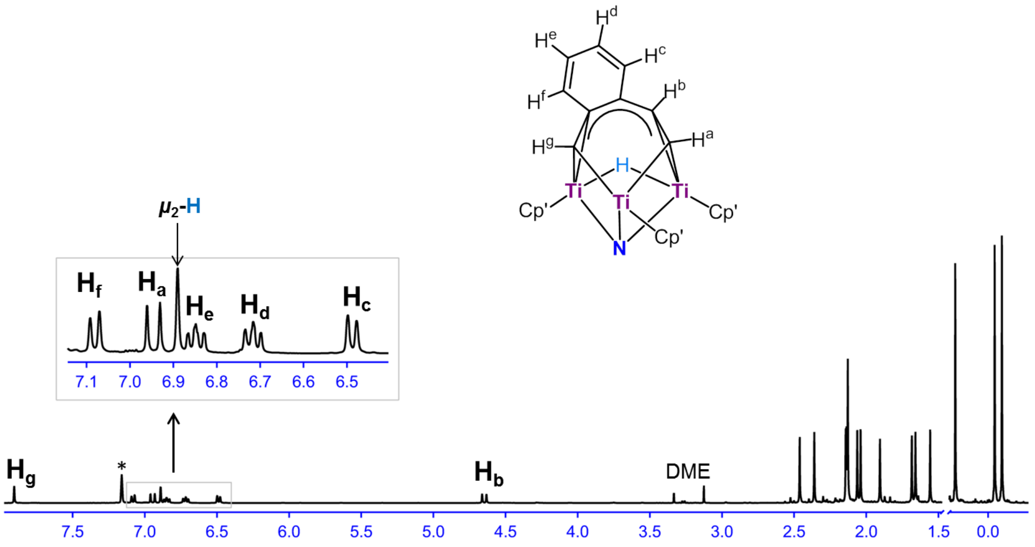

Supplementary Fig. 29 | ^{1}H NMR spectrum of 9 (C_6D_6, rt, * residual signals of C_6D_6)

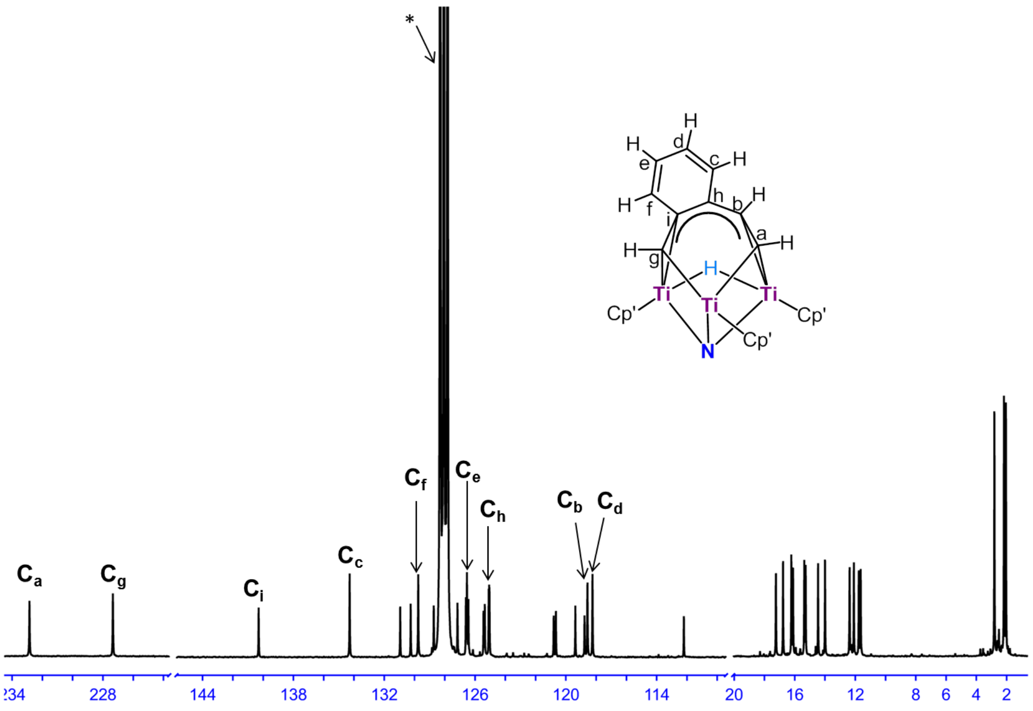

Supplementary Fig. 30 | ^{13}C NMR spectrum of 9 (C_6D_6, rt, * residual signals of C_6D_6)

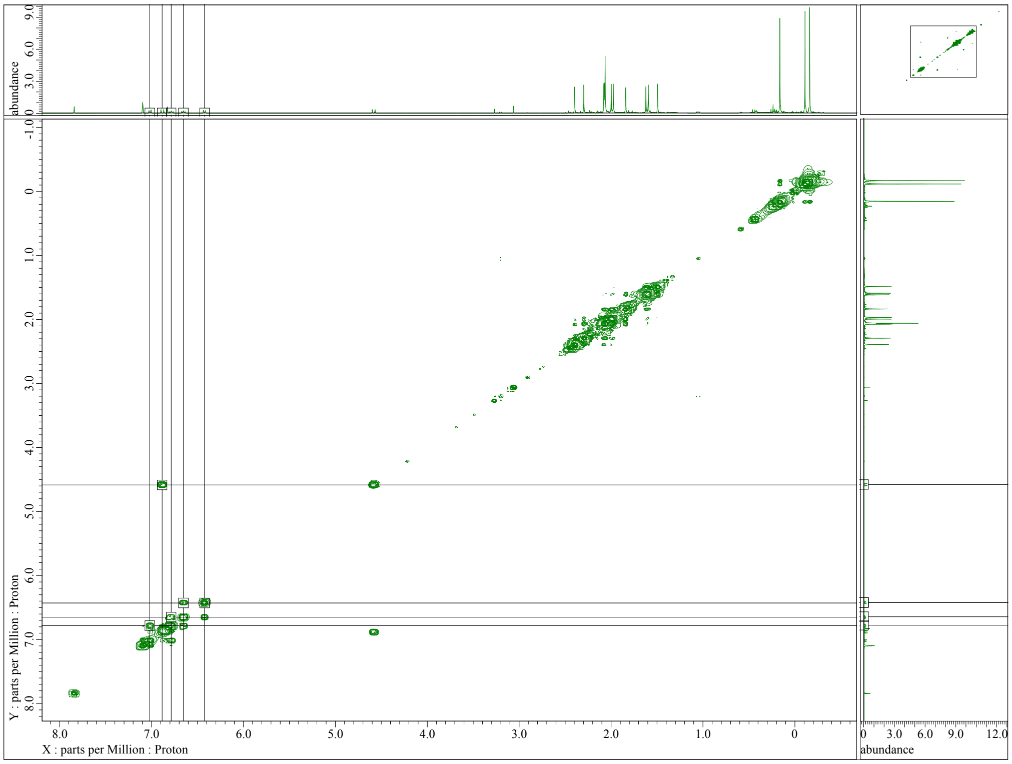

Supplementary Fig. 31 | H-H COSY NMR spectrum of 9 (C_6D_6, rt)

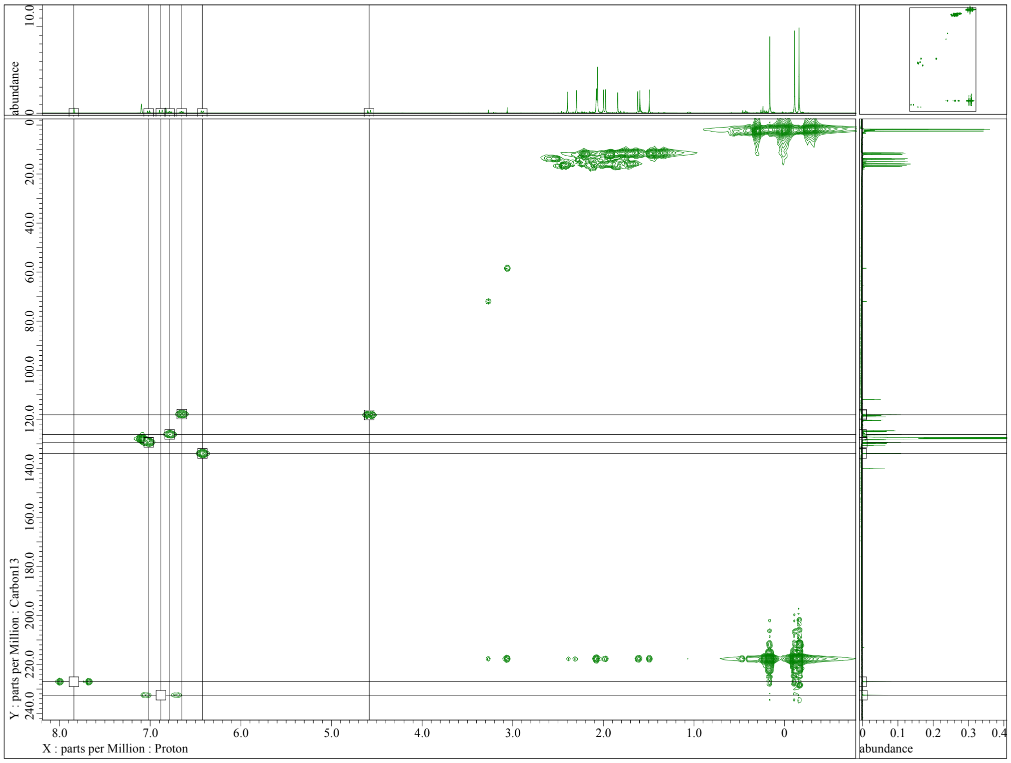

Supplementary Fig. 32 | HMQC NMR spectrum of 9 (C_6D_6, rt)

The reaction of 1 with pyridine-d_5

In an Ar glovebox, a J. Young-valve NMR tube was charged with 0.7 mL of THF-d_8 and complex **1** (15 mg, 0.0205 mmol). Pyridine-d_5 (16 mg, 0.202 mmol) was added into the NMR tube, which was then shaken and kept at room temperature for 5 min. A dark purple solution of **2-d_4** was obtained. When a C_6D_{12} solution of **2-d_4** was heated at 80 °C for 12h, the dark green **3-d_3** was formed almost quantitatively.

^{1}H NMR of **2-d_4** (THF-d_8, -60 °C): 7.90 (d, $J_{H,H}$ = 16.0 Hz, 1H, μ_2-H), 1.35 (d, $J_{H,H}$ = 8.0 Hz, 2H, μ_2-H), −6.10 (td, $J_{H,H}$ = 16.0 Hz, 8.0 Hz, 1H, μ_3-H).

^{2}H NMR of **2-d_4** (THF, -60 °C): 8.12 (brs, 1D, C_5D_4N) , 6.56 (brs, 1D, C_5D_4N), 5.83 (brs, 1D, C_5D_4N), 5.17 (brs, 1D , C_5D_4N).

^{1}H NMR of **3-d_4** (C_6D_{12}, rt): 6.74 (brs, 1H, C_5D_4H), 4.60 (brs, 1H, μ-H)

^{2}H NMR of **3-d_4** (C_6H_{12}, rt): 6.80 (brs, 1D, C_5D_4H), 6.28 (brs, 1D, C_5D_4H), 4.69 (brs, 2D, C_5D_4H)

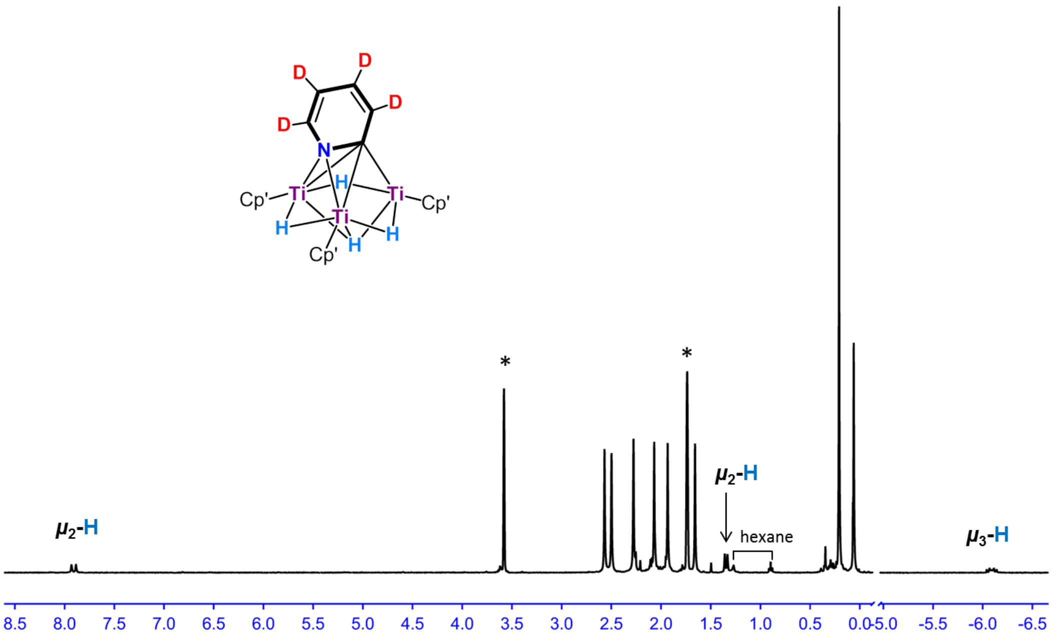

Supplementary Fig. 33 | ^{1}H NMR spectrum of 2-d_4 (THF-d_8, -60 ^{o}C, * residual signals of THF-d_8).

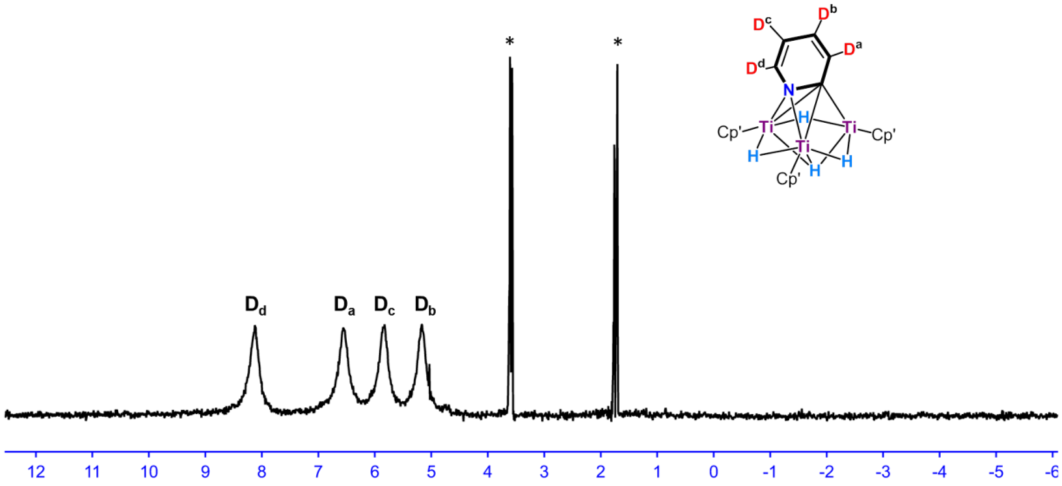

Supplementary Fig. 34 | ^{2}H NMR spectrum of 2-d_4 (THF, -60 ^{o}C, * residual signals of THF).

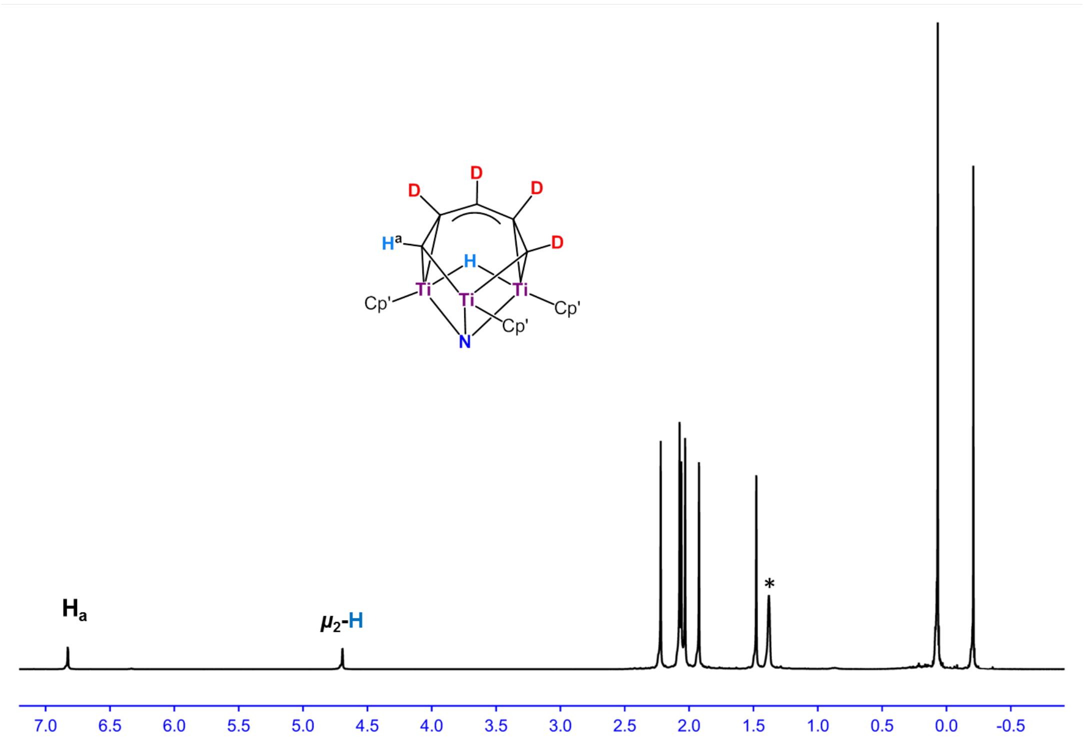

Supplementary Fig. 35 | ^{1}H NMR spectrum of 3-*d*$_4$ (C_6D_{12}, rt, * residual signals of C_6D_{12}).

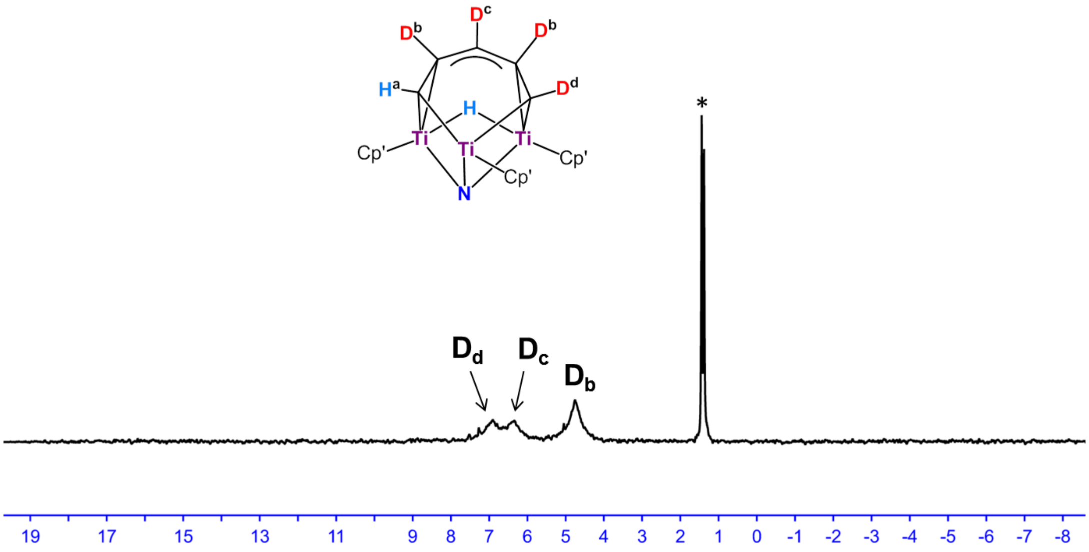

Supplementary Fig. 36 | ^{2}H NMR spectrum of 3-*d*$_4$ (C_6H_{12}, rt, * residual signals of C_6H_{12}).

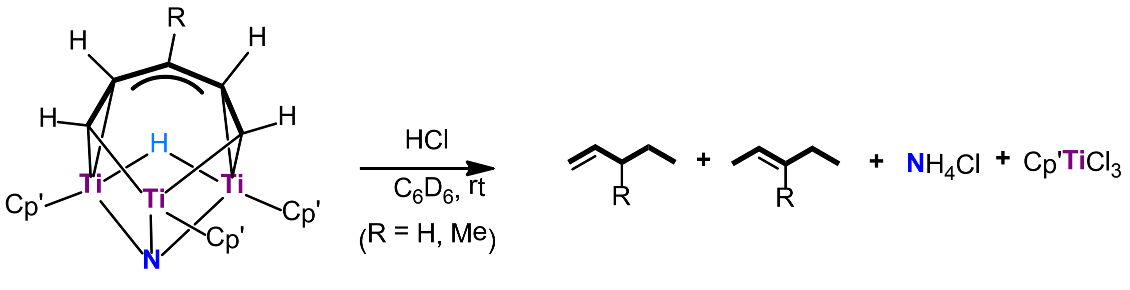

Reaction of 3 and 5 with Hydrochloric Acid

Hydrochloric acid (3.0 M HCl aqueous solution, 82 µL, 0.25 mmol) was slowly added to a C_6D_6 solution (0.8 mL) of **3** (20 mg, 0.025 mmol) via a syringe. An orange-red solution was formed immediately together with solid precipitates. After 10 min, the reaction mixture was separated by a trap-to-trap technique. The volatile part was dried with Na_2SO_4. The organic products were confirmed by GC-MS and NMR analyses through comparison with authentic samples. The ^{1}H NMR integration against the 1,2,4,5-tetramethylbenzene internal standard showed 1-pentene and 2-pentene in 48% and 14% yields, respectively. To the residue Et_2O was added to give a suspension, which was then centrifuged and filtered. The white solid was confirmed to be NH_4Cl (76% yield) by ^{1}H NMR analysis and phenol-hypochlorite titration. The solution was evaporated under vacuum. The resulting orange-red solid was washed with hexane and dried under vacuum to give Cp′$TiCl_3$ (23 mg, 0.066mmol, 88% yield).

In a similar way, the reaction of **5** with hydrochloric acid gave 3-methyl-1-pentene (31% yield), 3-methyl-2-pentene (16% yield), NH_4Cl (76% yield), and Cp′$TiCl_3$ (94% yield).

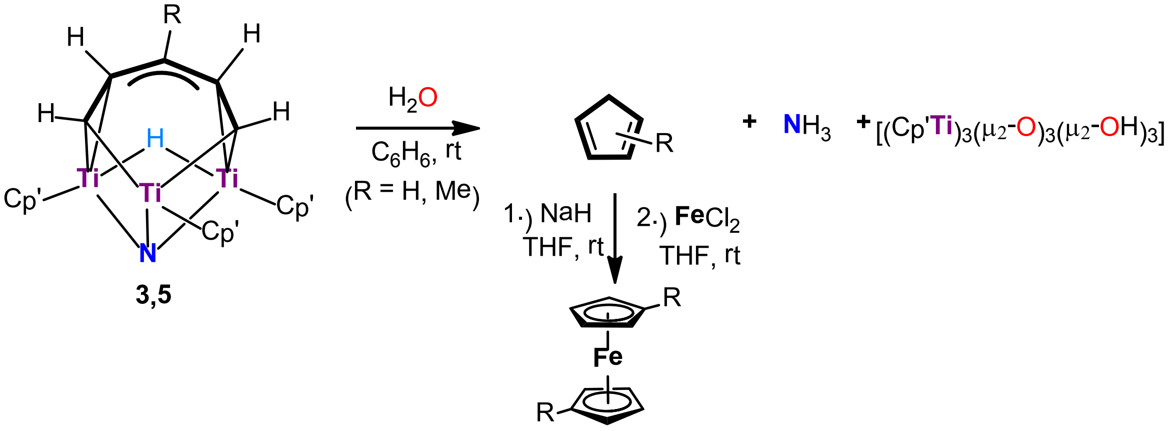

Reaction of 3 and 5 with Water

Degassed ultrapure H_2O (2.0 ml) was added to a 4 ml benzene solution of complex **3** (100 mg, 0.12 mmol) under vigorously stirring at room temperature. After 15min, the resulting dark-red suspension was separated by a "trap-to-trap" technique. The solid residue was extracted with hexane. The hexane solution was then concentrated and kept at −33 ^{o}C to give $[(C_5Me_4SiMe_3)Ti]_3(\mu_2\text{-}O)_3(\mu_2\text{-}OH)_3$ (65 mg, 0.079 mmol, 66% yield) as red crystals. Single crystals suitable for X-ray diffraction studies were obtained by recrystallization from THF/hexane (1:3).

The volatile part obtained in the above "trap-to-trap" separation was first dried with Na_2SO_4 and then filtered. NaH (30 mg, 1.25 mmol) was added to the filtrate and was then stirred at room temperature for 12 h. After filtration, the solvent was evaporated under vacuum, which gave sodium cyclopentadienylide (CpNa) as white solid (6 mg, 0.068 mmol). The resulting CpNa was dissolved in THF, to which $FeCl_2$ (5 mg, 0.34 mmol) was added and then stirred at room temperature for 12 h. After evaporation of the solvent under vacuum, the residue was extracted with hexane and filtered. The yellow filtrate was evaporated to give ferrocene (Cp_2Fe) (6 mg, 0.032 mmol, 52% yield), which was confirmed by an X-ray diffraction analysis. Alternatively, the volatile part obtained in the "trap-to-trap" step in a separated reaction of **3** with H_2O was treated with HCl (1.0 M in ether), which led to immediate formation of NH_4Cl as white solid (5 mg, 0.093 mmol, 75% yield).

The reaction of **3** with D_2O afforded the H/D scrambled cyclopentadiene, ammonia and $[(C_5Me_4SiMe_3)Ti]_3(\mu_2\text{-}O)_3(\mu_2\text{-}OD)_3$, as confirmed by NMR and IR analyses.

In a similar manner, the reaction of **5** (100 mg, 0.12 mmol) with water afforded methylcyclopentadienes (confirmed by formation of $(C_5H_4Me)_2Fe$: 8 mg, 0.037 mmol, 61%

yield), $[(C_5Me_4SiMe_3)Ti]_3(\mu_2\text{-}O)_3(\mu_2\text{-}OH)_3$ (68 mg, 0.088 mmol, 72% yield), and ammonia (in the form of NH_4Cl, 4 mg, 0.093 mmol, 61% yield).

$[(C_5Me_4SiMe_3)Ti]_3(\mu_2\text{-}O)_3(\mu_2\text{-}OH)_3$

^1H NMR (C_6D_6, rt): 2.46 (s, 3H, OH), 2.16 (s, 18H, $C_5Me_4SiMe_3$), 1.89 (s, 18H, $C_5Me_4SiMe_3$), 0.44 (s, 27H, $C_5Me_4SiMe_3$). ^{13}C NMR (C_6D_6, rt): 125.7, 122.3 (s, $C_5Me_4SiMe_3$), 110.3 (s, *ipso*-$C_5Me_4SiMe_3$), 14.3, 11.2 (s, $C_5Me_4SiMe_3$), 2.4 (s, $C_5Me_4SiMe_3$). IR (Nujol mull) 3646, 1338, 1246, 1090, 843, 782,cm^{-1}.

$[(C_5Me_4SiMe_3)Ti]_3(\mu_2\text{-}O)_3(\mu_2\text{-}OD)_3$

^2H NMR of (C_6H_6, rt): 2.46 (brs, *OD*) IR (Nujol mull) 2688, 1338, 1245, 1091, 842, 753cm^{-1}.

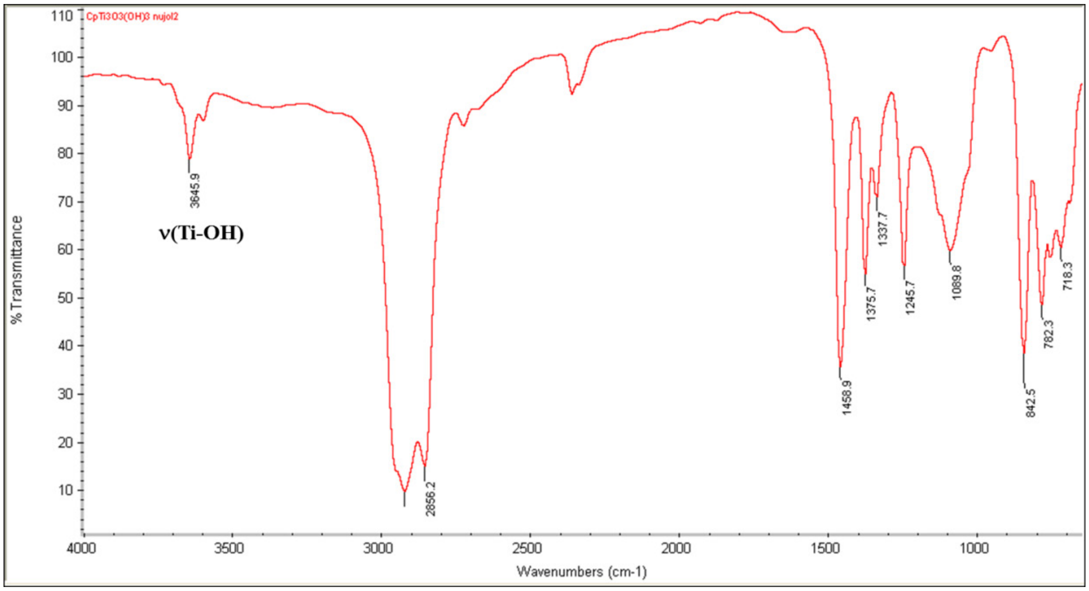

Supplementary Fig. 37 | IR of $[(C_5Me_4SiMe_3)Ti]_3(\mu_2\text{-}O)_3(\mu_2\text{-}OH)_3$ (nujol)

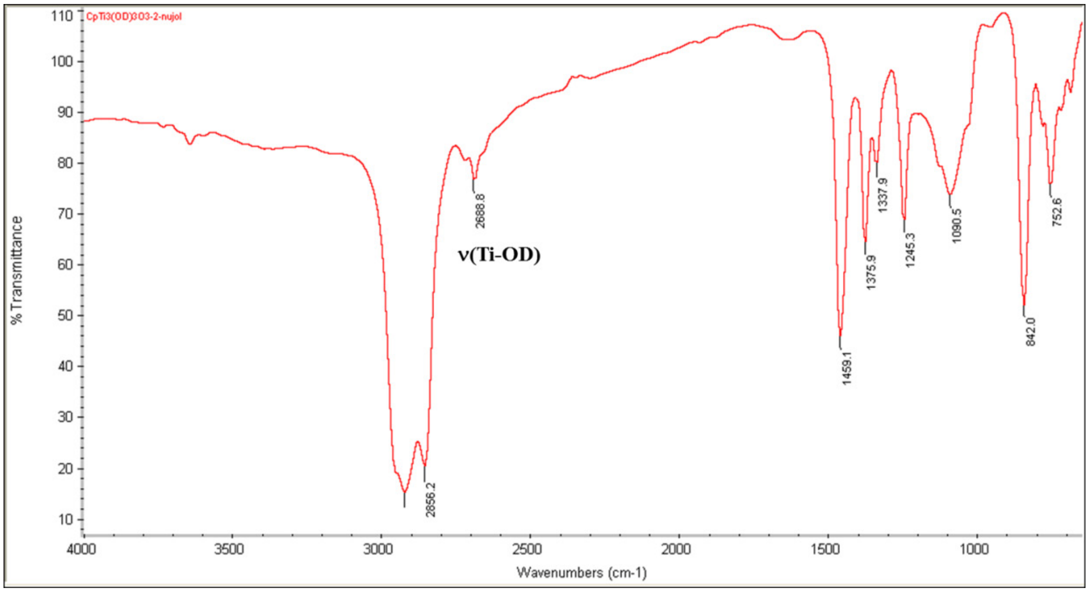

Supplementary Fig. 38 | IR of $[(C_5Me_4SiMe_3)Ti]_3(\mu\text{-}O)_3(\mu\text{-}OD)_3$ (nujol)

NMR Analyses of the *In-situ* Generated Cyclopentadienes

After the reaction of complex **3** (30 mg, 0.037 mmol) with H_2O (5.0 M in THF, 75 µl, 0.37 mmol) in THF-d_8, the volatile part was transferred to a J. young NMR tube through trap-to-trap. The formation of cyclopentadiene was confirmed by the subsequent ^{1}H NMR analysis (Supplementary Fig. 39). The formation of the H/D scrambled cyclopentadiene in the reaction of **3** (10 mg, 0.012 mmol) with D_2O (25 µl, 0.12 mmol) in THF was confirmed similarly by the ^{2}H NMR analysis (Supplementary Fig. 40). The formation of the methylcyclopentadiene isomers in the reaction of **5** (20 mg, 0.024 mmol) with water (neat, 5 µl, 0.24 mmol) was also confirmed by the ^{1}H NMR analysis (Supplementary Fig. 41).

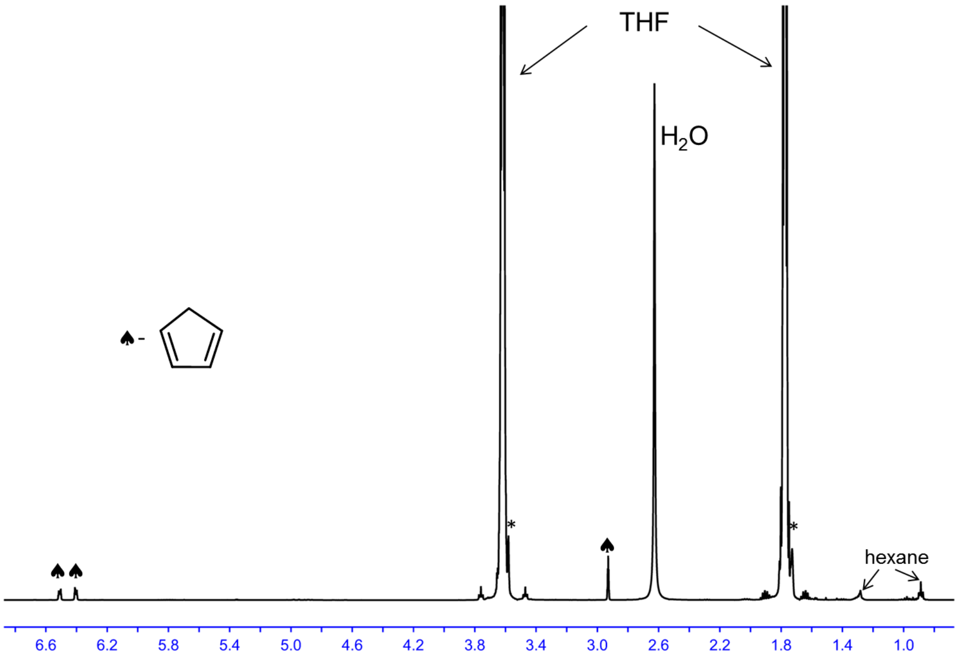

Supplementary Fig. 39 | ^{1}H NMR spectrum of solvent part of 3 with water (THF-d_8, rt, * residual signals of THF-d_8)

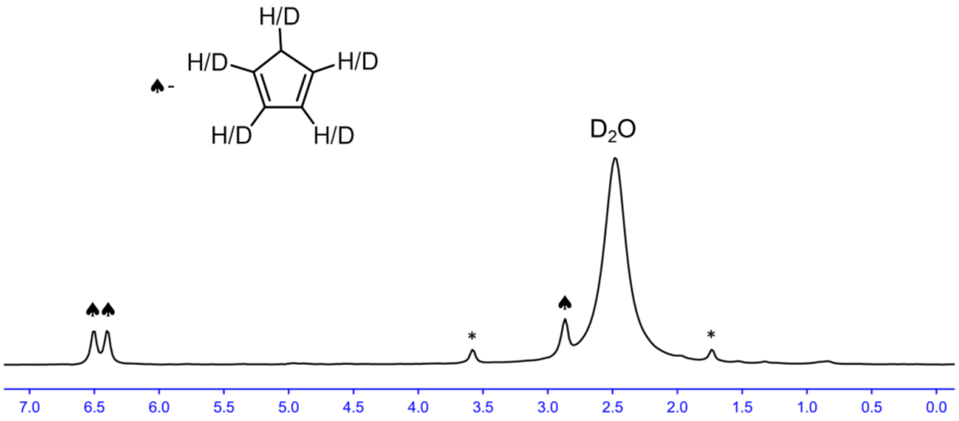

Supplementary Fig. 40 | ^{2}H NMR spectrum of solvent part of 3 with D_2O (THF, rt)

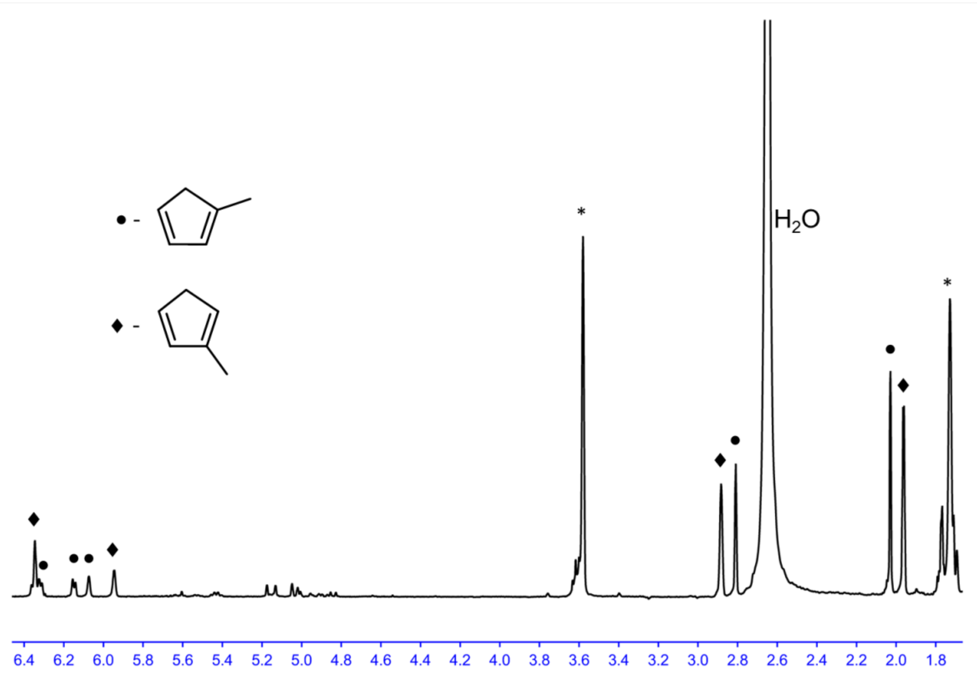

Supplementary Fig. 41 | ^{1}H NMR spectrum of solvent part of 5 with water (THF-d_8, rt, * residual signals of THF-d_8)

Transformation of [($C_5Me_4SiMe_3$)Ti]$_3$(μ_2-O)$_3$(μ_2-OH)$_3$ to ($C_5Me_4SiMe_3$)$TiCl_3$

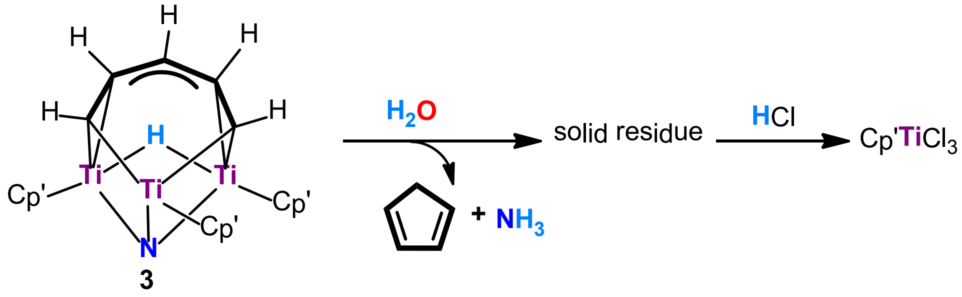

Degassed ultrapure H_2O (1 ml) was added to a 2 ml benzene solution of complex **3** (50 mg, 0.062 mmol) under vigorous stirring. After 15 min, the reaction mixture was separated by "trap-to-trap" under vacuum. The solid residue was treated with an ether solution of HCl (1.0 M, 100 µl). The resulting orange–red solution was evaporated. The solid was washed with cold hexane and dried under vacuum to give ($C_5Me_4SiMe_3$)$TiCl_3$ (60 mg, 0.17 mmol, 93%). Alternatively, ($C_5Me_4SiMe_3$)$TiCl_3$ was obtained quantitatively by a similar treatment of the isolated [($C_5Me_4SiMe_3$)Ti]$_3$(μ_2-O)$_3$(μ_2-OH)$_3$with HCl.

Titanium-Mediated Cycle for Hydrodenitrogenation of Pyridine by H_2

The reaction of $(C_5Me_4SiMe_3)TiCl_3$ with 3 equivalent of $LiCH_2SiMe_3$ in toluene gave $(C_5Me_4SiMe_3)Ti(CH_2SiMe_3)_3$ in 63% yield, which upon hydrogenolysis with H_2 (4 atm) afforded the hydride cluster complex **1** in 69% yield[3]. The reaction of **1** with pyridine at 60 ºC yielded **3**, which upon treatment with HCl regenerated $(C_5Me_4SiMe_3)TiCl_3$ with release of 1-pentene/2-pentene and NH_4Cl. When **3** was treated with H_2O, $[(C_5Me_4SiMe_3)Ti]_3(\mu_2\text{-O})_3(\mu_2\text{-OH})_3$was formed with release of cyclopentadiene and NH_3. Treatment of $[(C_5Me_4SiMe_3)Ti]_3(\mu_2\text{-O})_3(\mu_2\text{-OH})_3$ with HCl then regenerated $(C_5Me_4SiMe_3)TiCl_3$.

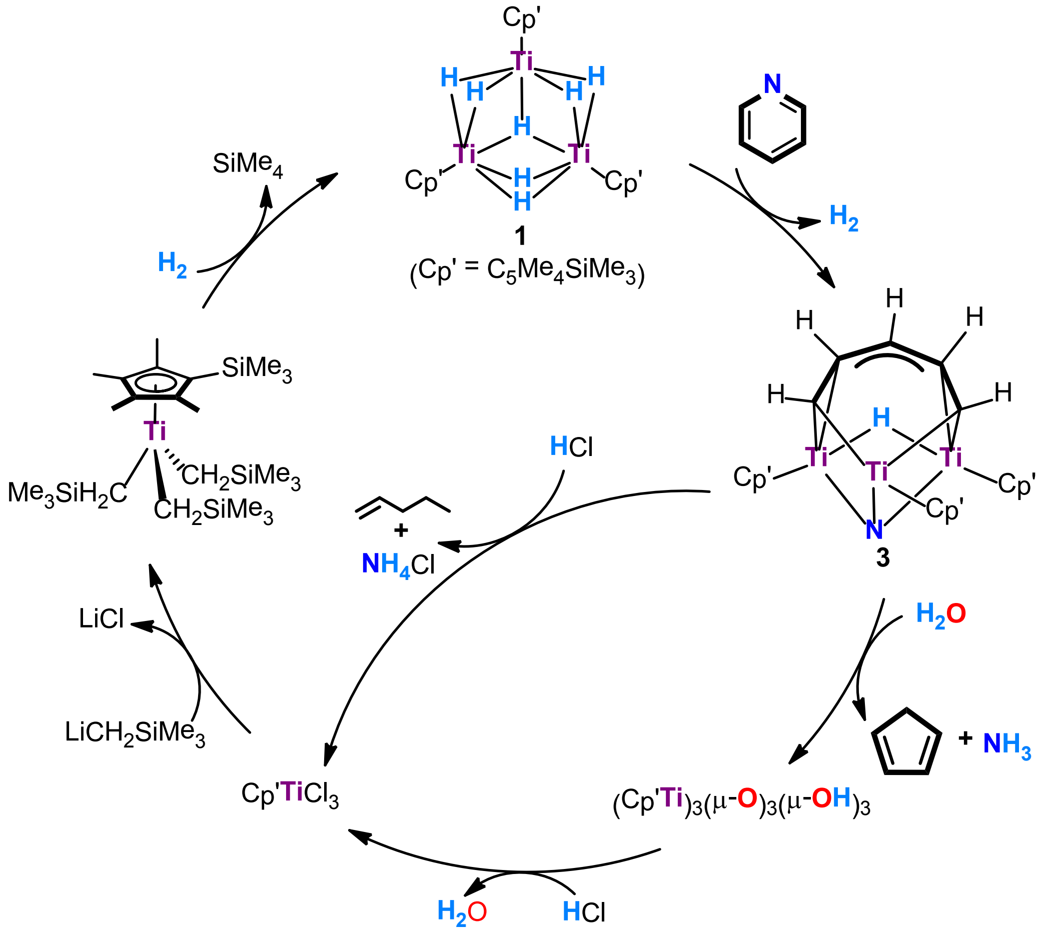

Supplementary Fig. 42 | Titanium-mediated cycle for the hydrodenitrogenation of pyridine by H_2.

Kinetic Studies of Conversion of 2 to 3

In a typical experiment, a 5-mm NMR tube with a J. Young valve was charged with 10 mg of **2** (0.0112 mmol) and toluene-d_8 (0.70 ml) in a glovebox, and was then subjected to the ^{1}H NMR measurements. The relative concentration was calculated based on the integration of the resonances of the $SiMe_3$ groups in the $C_5Me_4SiMe_3$ ligands. The activation parameters for the conversion of **2** to **3** were determined by the rate constants obtained at various temperatures (Supplementary Fig. 43). An Eyring plot (Supplementary Fig. 44) was constructed from these data (Supplementary Table 1). The activation enthalpy and entropy were extracted as follows: $\Delta H^{\neq} = 25.3$ (5) kcal/mol, $\Delta S^{\neq} = -0.6(14)$ eu.

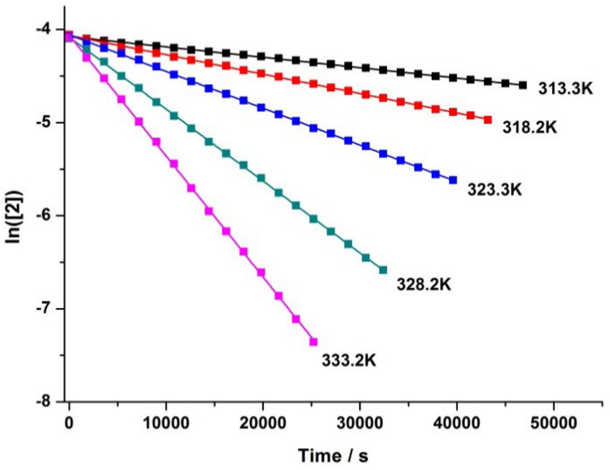

Supplementary Fig. 43 | First order plots for the disappearance of 2 as a function of time at various temperatures.

Supplementary Table 1 | Observed rate constants (k_{obs}) for the conversion of 2 to 3.

Temperature (K)	k_{obs} (s^{-1})
313.3	1.11×10^{-5}
318.2	2.08×10^{-5}
323.3	3.95×10^{-5}
328.2	7.73×10^{-5}
333.2	1.30×10^{-4}

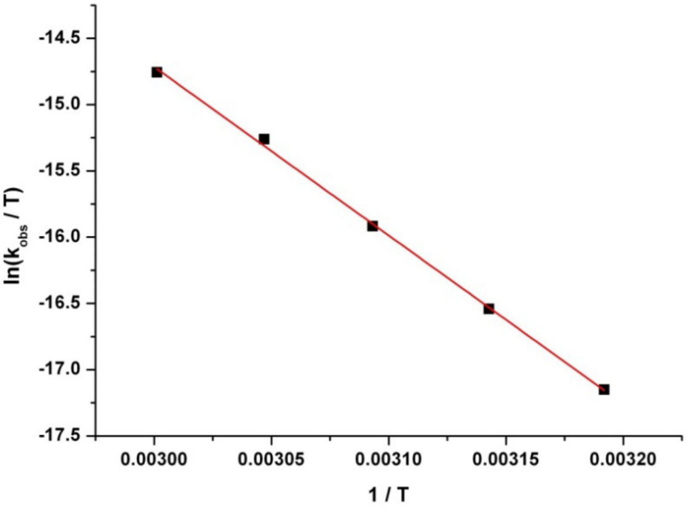

Supplementary Fig. 44 | Eyring plot for the conversion of 2 to 3.

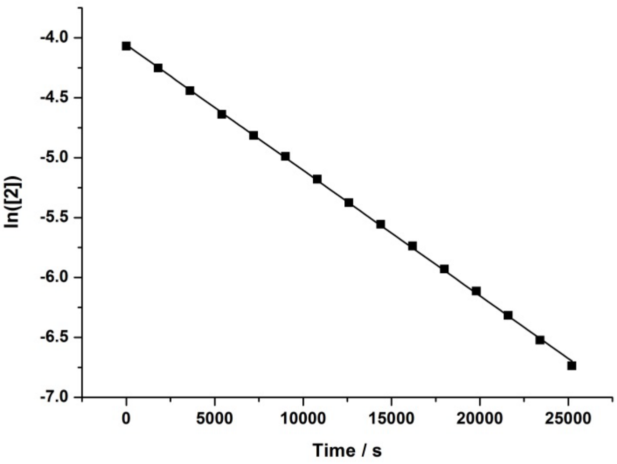

Supplementary Fig. 45 | First order plots for the conversion of 2 to 3 under H_2 atmosphere at 60 °C, $k_{(H2)}$ = 1.05×10^{-4} s^{-1}

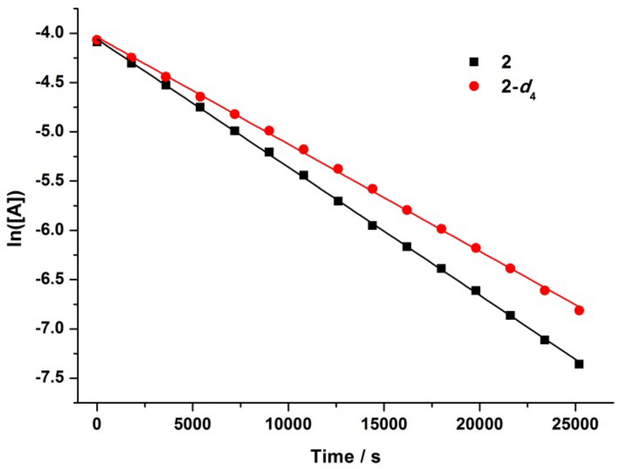

Supplementary Fig. 46 | First order plots for the conversion of 2 (black) and 2-d_4 (red) at 60 ^{o}C, k_D = 1.09×10^{-4} s^{-1}. k_H/k_D = 1.19.

X-ray Crystallographic Studies

Crystals for X-ray diffraction studies were obtained as described in the preparations. The crystals were manipulated in a glovebox under a microscope, and were sealed in thin-walled glass capillaries. X-ray diffraction data collections were performed on a Bruker D8 QUEST diffractometer equipped with a CMOS area detector, using a IμS (Incoatec Microfocus Source) microfocus sealed tube with Mo Kα radiation (λ = 0.71073 Å) at 173 K. The Bravais lattice and the unit cell parameters were determined by the Bruker APEX2 software package[4]. The raw frame data were processed, and absorption corrections were done using SAINT and SADABS embedded in Bruker APEX2 to yield the reflection data (hkl) file[4]. All of the structures were solved using SHELXS-2013[5]. Structural refinement was performed using the SHELXL option in the WINGX system[6], on F^2 anisotropically for all of the non-hydrogen atoms by the full matrix least-squares method. Analytical scattering factors for neutral atoms were used throughout the analysis. The hydrogen atoms of the Cp′ ligands were placed at the calculated positions, which were refined using a riding model. Other hydrogen atoms could be located on difference Fourier maps and refined. The analytical scattering factors for neutral atoms were used throughout the analysis. The residual electron densities were of no chemical significance. Disordered solvent (hexane) in complex **6** was removed using PLATON SQUEEZE.

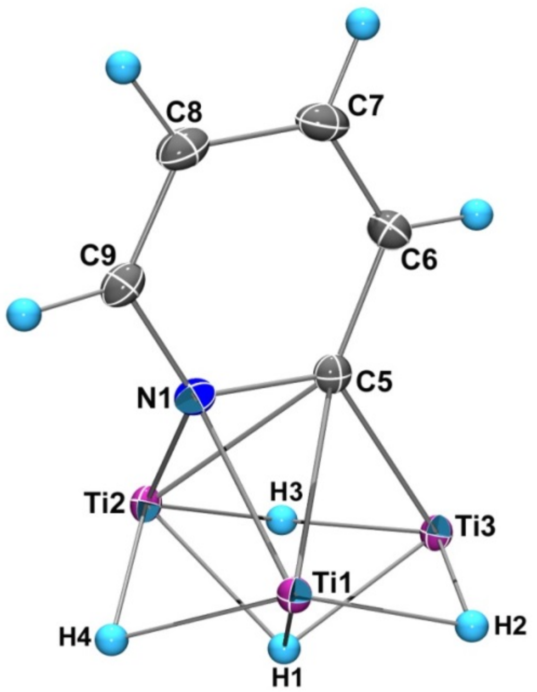

Supplementary Fig. 47 | Solid state molecular structure of 2 at 30% probability ellipsoids. Cp′ ligands are omitted for clarity.

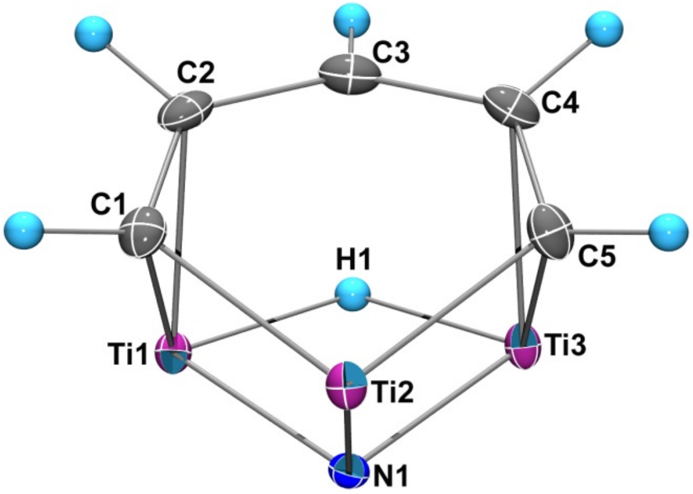

Supplementary Fig. 48 | Solid state molecular structure of 3 at 30% probability ellipsoids. Cp′ ligands are omitted for clarity.

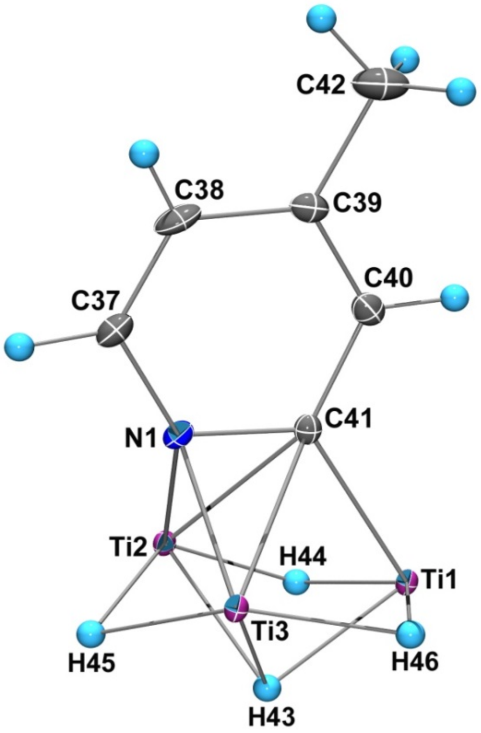

Supplementary Fig. 49 | Solid state molecular structure of 4 at 30% probability ellipsoids. Cp′ ligands are omitted for clarity.

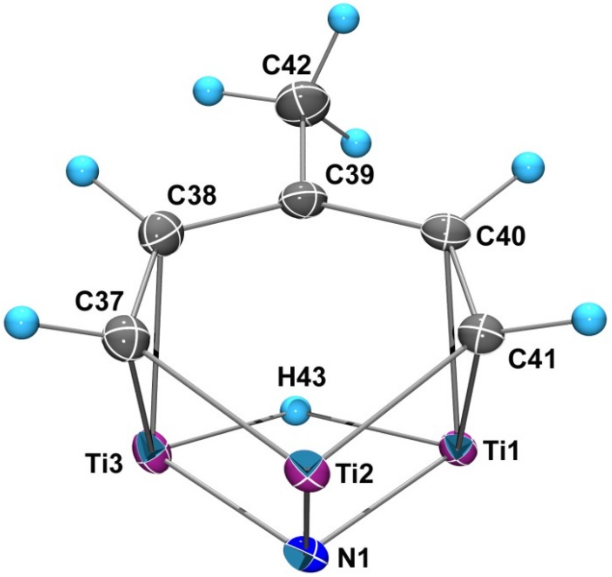

Supplementary Fig. 50 | Solid state molecular structure of 5 at 30% probability ellipsoids. Cp′ ligands are omitted for clarity.

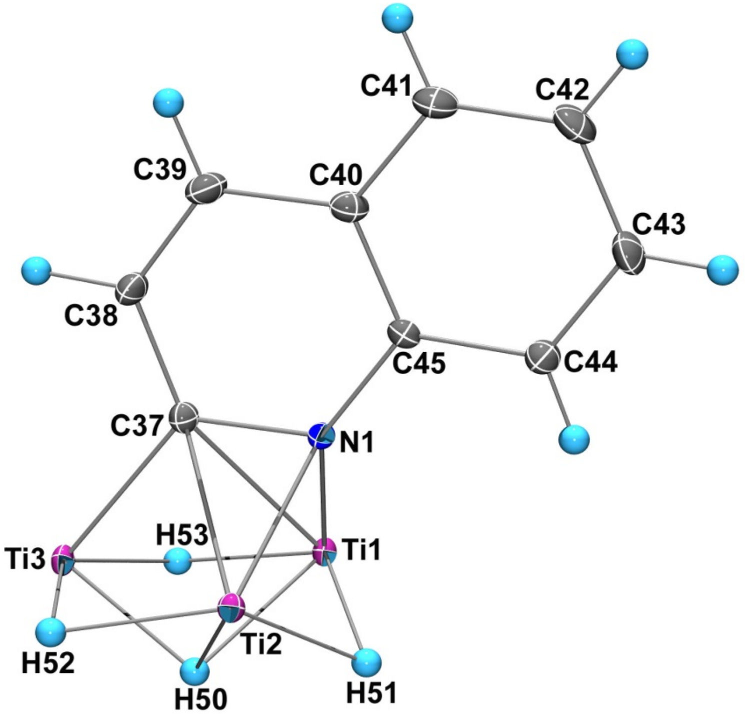

Supplementary Fig. 51 | Solid state molecular structure of 6 at 30% probability ellipsoids. Cp′ ligands are omitted for clarity.

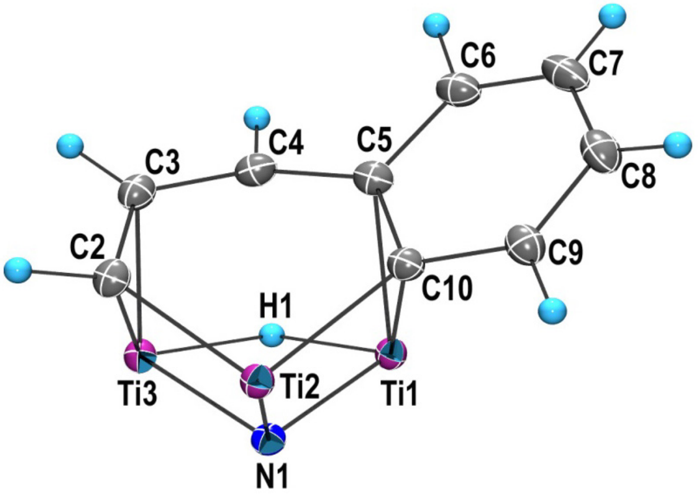

Supplementary Fig. 52 | Solid state molecular structure of 7 at 30% probability ellipsoids. Cp′ ligands are omitted for clarity.

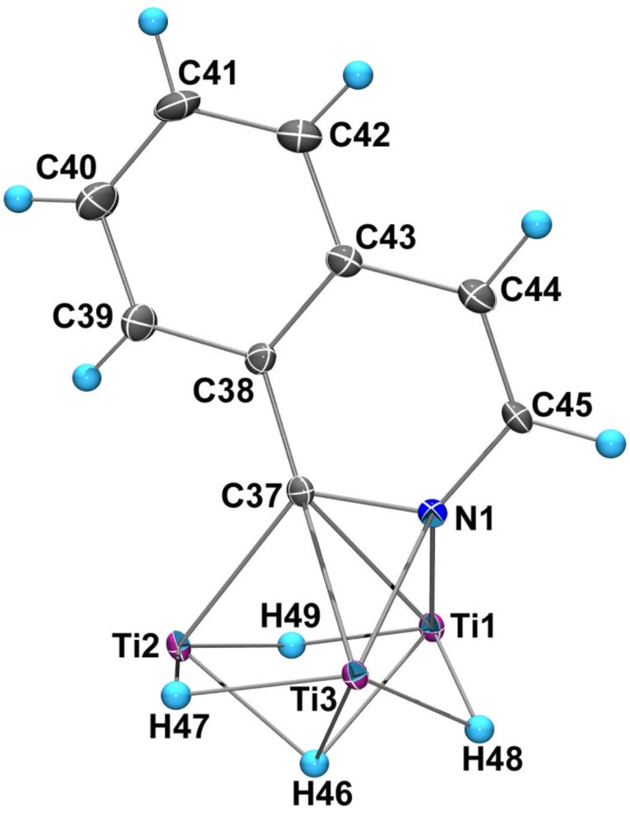

Supplementary Fig. 53 | Solid state molecular structure of 8 at 30% probability ellipsoids. Cp′ ligands are omitted for clarity.

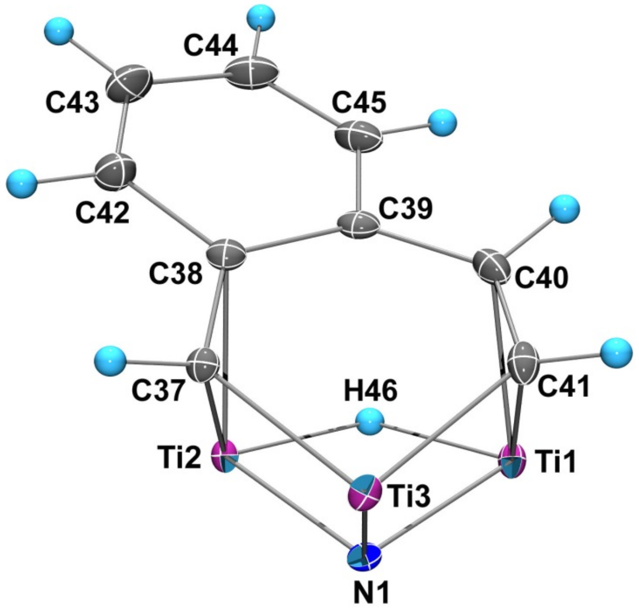

Supplementary Fig. 54 | Solid state molecular structure of 9 at 30% probability ellipsoids. Cp′ ligands are omitted for clarity.

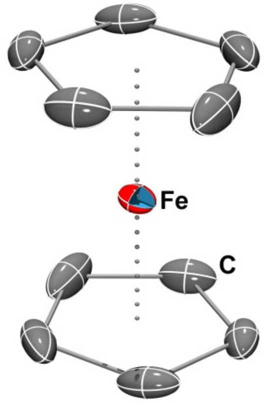

Supplementary Fig. 55 | Solid state molecular structure of ferrocene at 30% probability ellipsoids. Cp′ are ligands omitted for clarity.

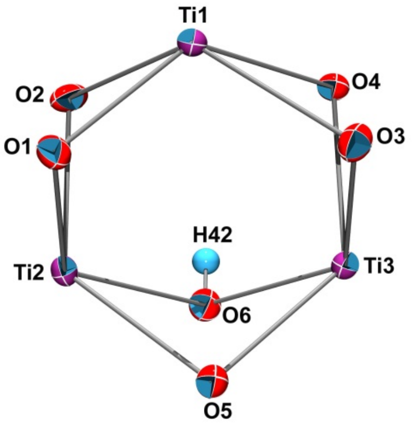

Supplementary Fig. 56 | Solid state molecular structure of [($C_5Me_4SiMe_3$)Ti]$_3$(μ_2-O)$_3$(μ_2-OH)$_3$ at 30% probability ellipsoids. Cp′ ligands are omitted for clarity. Only one hydroxyl proton H42 was found on the difference Fourier maps.

Supplementary Table 2 | Crystal data and structure refinement for 2.

Identification code	CCDC 1535992
Empirical formula	$C_{42}H_{74}Si_3Ti_3$
Formula weight	805.95
Temperature	173(2) K
Wavelength	0.71073 Å
Crystal system	Monoclinic
Space group	$P2_1/c$
Unit cell dimensions	a = 19.8980 (14) Å α= 90 °.
	b = 11.2053 (8) Å β= 104.008 (2)°.
	c = 20.1236 (12) Å γ = 90 °.
Volume	4353.4 (5) Å^3
Z	4
Density (calculated)	1.230 Mg/m^3
F(000)	1728
Theta range for data collection	1.1° to 25.00°
Index ranges	-23<=h<=23, -13<=k<=13, -23<=l<=23
Reflections collected	88917
Independent reflections	7705 [R(int) = 0.075]
Absorption correction	multi-scan
Refinement method	full-matrix least-squares on F^2
Data / restraints / parameters	7705 / 0 / 470
Goodness-of-fit on F^2	1.285
Final R indices [I>2sigma(I)]	R1 = 0.0507, wR2 = 0.1196
R indices (all data)	R1 = 0.0666, wR2 = 0.1264
Largest diff. peak and hole	0.54 and -0.52 e. Å^{-3}

Supplementary Table 3 | Crystal data and structure refinement for 3.

Identification code	CCDC 1535993
Empirical formula	$C_{41}H_{69}NSi_3Ti_3$
Formula weight	803.94
Temperature	173(2) K
Wavelength	0.71073 Å
Crystal system	Monoclinic
Space group	$P2_1/c$
Unit cell dimensions	a = 11.4412 (10) Å α= 90 °.
	b = 16.4664 (15) Å β= 102.987 (4)°.
	c = 23.630 (2) Å γ = 90 °.
Volume	4337.8 (7) $Å^3$
Z	4
Density (calculated)	1.231 Mg/m^3
F(000)	1720
Theta range for data collection	1.8° to 25.1°
Index ranges	-13<=h<=13, -19<=k<=19, -28<=l<=28
Reflections collected	93080
Independent reflections	7738 [R(int) = 0.115]
Absorption correction	multi-scan
Refinement method	full-matrix least-squares on F^2
Data / restraints / parameters	7738 / 0 / 458
Goodness-of-fit on F^2	1.086
Final R indices [I>2sigma(I)]	R1 = 0.0440, wR2 = 0.1026
R indices (all data)	R1 = 0.0739, wR2 = 0.1169
Largest diff. peak and hole	0.36 and -0.33 e. $Å^{-3}$

Supplementary Table 4 | Crystal data and structure refinement for 4.

Identification code	CCDC 1535995
Empirical formula	$C_{42}H_{73}NSi_3Ti_3$
Formula weight	819.98
Temperature	173(2) K
Wavelength	0.71073 Å
Crystal system	Trigonal
Space group	$P3_1$
Unit cell dimensions	a = 11.5069 (13) Å α= 90 °.
	b = 11.5069(13)Å β= 90 °
	c = 29.933 (4) Å γ = 120°
Volume	3432.4 (9) $Å^3$
Z	3
Density (calculated)	1.190 Mg/m^3
F(000)	1320
Theta range for data collection	2.5° to 25.0°
Index ranges	-13<=h<=13, -13<=k<=13, -35<=l<=35
Reflections collected	76108
Independent reflections	8101 [R(int) = 0.088]
Absorption correction	multi-scan
Refinement method	full-matrix least-squares on F^2
Data / restraints / parameters	8101 / 1 / 470
Goodness-of-fit on F^2	0.956
Final R indices [I>2sigma(I)]	R1 = 0.0315, wR2 = 0.0673
R indices (all data)	R1 = 0.0406, wR2 = 0.0712
Largest diff. peak and hole	0.38 and -0.32 e. $Å^{-3}$
absolute structure Flack	0.023(11)

Supplementary Table 5 | Crystal data and structure refinement for 5.

Identification code	CCDC 1536094
Empirical formula	$C_{42}H_{71}NSi_3Ti_3$
Formula weight	817.96
Temperature	173(2) K
Wavelength	0.71073 Å
Crystal system	Triclinic
Space group	$P\bar{1}$
Unit cell dimensions	a = 10.130 (3) Å α= 99.125 (11)°
	b = 11.681 (3) Å β= 92.816 (11)°
	c = 19.410 (5) Å γ = 91.900 (11)°
Volume	2262.8 (10)Å^3
Z	2
Density (calculated)	1.201 Mg/m^3
F(000)	876
Theta range for data collection	1.1° to 25.1°
Index ranges	-12<=h<=12, -13<=k<=13, -23<=l<=23
Reflections collected	49649
Independent reflections	7984 [R(int) = 0.088]
Absorption correction	multi-scan
Refinement method	full-matrix least-squares on F^2
Data / restraints / parameters	7984 / 1 / 430
Goodness-of-fit on F^2	1.002
Final R indices [I>2sigma(I)]	R1 = 0.0567, wR2 = 0.1309
R indices (all data)	R1 = 0.0850, wR2 = 0.1469
Largest diff. peak and hole	0.87 and -0.96 e. Å^{-3}

Supplementary Table 6 | Crystal data and structure refinement for 6.

Identification code	CCDC 1535991
Empirical formula	$C_{45}H_{73}NSi_3Ti_3$
Formula weight	856.01
Temperature	173(2) K
Wavelength	0.71073 Å
Crystal system	Triclinic
Space group	$P\bar{1}$
Unit cell dimensions	a = 11.2599 (9) Å α= 97.203 (3)°.
	b = 11.3810 (8) Å β= 93.540 (3)°
	c = 22.4454 (18) Å γ = 117.872 (3)°
Volume	2498.7 (3) $Å^3$
Z	2
Density (calculated)	1.138 Mg/m^3
F(000)	916
Theta range for data collection	0.9° to 26.3°
Index ranges	-14<=h<=14, -14<=k<=14, -27<=l<=27
Reflections collected	60221
Independent reflections	10123 [R(int) = 0.080]
Absorption correction	multi-scan
Refinement method	full-matrix least-squares on F^2
Data / restraints / parameters	10123 / 0 / 509
Goodness-of-fit on F^2	0.940
Final R indices [I>2sigma(I)]	R1 = 0.0388, wR2 = 0.0942
R indices (all data)	R1 = 0.0597, wR2 = 0.1079
Largest diff. peak and hole	0.38 and -0.26 e. $Å^{-3}$

Supplementary Table 7 | Crystal data and structure refinement for 7.

Identification code	CCDC 1535989
Empirical formula	$C_{45}H_{71}NSi_3Ti_3$
Formula weight	853.99
Temperature	173(2) K
Wavelength	0.71073 Å
Crystal system	Monoclinic
Space group	$P2_1$
Unit cell dimensions	a = 9.8610 (18) Å α= 90.0°.
	b = 20.748 (3) Å β= 97.984 (4)°
	c = 11.347 (2) Å γ = 90.0°.
Volume	2299.0 (7) $Å^3$
Z	4
Density (calculated)	1.234 Mg/m^3
F(000)	912
Theta range for data collection	3.1° to 27.5°
Index ranges	-12<=h<=9, -25<=k<=26, -14<=l<=14
Reflections collected	19070
Independent reflections	9703 [R(int) = 0.048]
Absorption correction	Multi-scan
Refinement method	Full-matrix least-squares on F^2
Data / restraints / parameters	9703 / 2 / 501
Goodness-of-fit on F^2	1.057
Final R indices [I>2sigma(I)]	R1 = 0.0415, wR2 = 0.0936
R indices (all data)	R1 = 0.0471, wR2 = 0.1012
Largest diff. peak and hole	0.38 and -0.31 e. $Å^{-3}$
absolute structure Flack	-0.010(12)

Supplementary Table 8 | Crystal data and structure refinement for 8.

Identification code	CCDC 1535990
Empirical formula	$C_{45}H_{73}NSi_3Ti_3$
Formula weight	856.01
Temperature	173(2) K
Wavelength	0.71073 Å
Crystal system	Trigonal
Space group	$P3_2$
Unit cell dimensions	a = 11.5135 (11) Å α= 90.0°.
	b = 11.5135(11)Å β= 90.0°.
	c = 30.919(3)Å γ = 120.0°.
Volume	3549.6(8) $Å^3$
Z	3
Density (calculated)	1.201 Mg/m^3
F(000)	1374
Theta range for data collection	2.0° to 25.0°
Index ranges	-13<=h<=13, -13<=k<=13, -36<=l<=36
Reflections collected	78222
Independent reflections	8357 [R(int) = 0.093]
Absorption correction	multi-scan
Refinement method	full-matrix least-squares on F^2
Data / restraints / parameters	8357 / 1 / 509
Goodness-of-fit on F^2	0.941
Final R indices [I>2sigma(I)]	R1 = 0.0297, wR2 = 0.0671
R indices (all data)	R1 = 0.0356, wR2 = 0.0699
Largest diff. peak and hole	0.24 and -0.18 e. $Å^{-3}$
absolute structure Flack	0.022 (10)

Supplementary Table 9 | Crystal data and structure refinement for 9.

Identification code	CCDC 1535994
Empirical formula	$C_{45}H_{71}NSi_3Ti_3$
Formula weight	853.91
Temperature	173(2) K
Wavelength	0.71073 Å
Crystal system	Monoclinic
Space group	$P2_1/n$
Unit cell dimensions	a = 10.2991 (13) Å α= 90.0°.
	b = 23.613 (3) Å β= 93.484 (6)°
	c = 19.026 (3) Å γ = 90.0°.
Volume	4618.5 (11) $Å^3$
Z	4
Density (calculated)	1.228 Mg/m^3
F(000)	1824
Theta range for data collection	1.4° to 25.1°
Index ranges	-12<=h<=11, -28<=k<=28, -22<=l<=22
Reflections collected	101832
Independent reflections	8206 [R(int) = 0.129]
Absorption correction	multi-scan
Refinement method	full-matrix least-squares on F^2
Data / restraints / parameters	8206 / 0 / 501
Goodness-of-fit on F^2	1.058
Final R indices [I>2sigma(I)]	R1 = 0.0418, wR2 = 0.0946
R indices (all data)	R1 = 0.0707, wR2 = 0.1069
Largest diff. peak and hole	0.36and -0.29 e. $Å^{-3}$

Supplementary Table 10 | Crystal data and structure refinement for $[(C_5Me_4SiMe_3)Ti]_3(\mu$-$O)_3(\mu$-$OH)_3$.

Identification code	CCDC 1538069
Empirical formula	$C_{36}H_{64}O_6Si_3Ti_3 \cdot C_4H_8O$
Formula weight	892.86
Temperature	173(2) K
Wavelength	0.71073 Å
Crystal system	Monoclinic
Space group	$P2_1/n$
Unit cell dimensions	a = 17.806 (3) Å α= 90.0°.
	b = 14.032 (3) Å β= 92.305 (7)°°
	c = 18.710 (4) Å γ = 90.0°.
Volume	4671.1 (15) $Å^3$
Z	4
Density (calculated)	1.270 Mg/m^3
F(000)	1904
Theta range for data collection	1.6° to 25.1°
Index ranges	-21<=h<=21, -15<=k<=16, -22<=l<=22
Reflections collected	77915
Independent reflections	8306 [R(int) = 0.162]
Absorption correction	multi-scan
Refinement method	full-matrix least-squares on F^2
Data / restraints / parameters	8306 / 0 /514
Goodness-of-fit on F^2	1.098
Final R indices [I>2sigma(I)]	R1 = 0.0604, wR2 = 0.1312
R indices (all data)	R1 = 0.0934, wR2 = 0.1431
Largest diff. peak and hole	0.57 and -0.37 e. $Å^{-3}$

Computational Analyses

Due to relatively large molecular size, the ligands of $C_5Me_4SiMe_3$ in each structure were replaced by $C_5H_4SiH_3$ for the mechanism calculations. For example, $[(C_5H_4SiH_3)Ti]_3(\mu_3\text{-H})(\mu_2\text{-H})_6$ (**1m**) was used for modelling $[(C_5Me_4SiMe_3)Ti]_3(\mu_3\text{-H})(\mu_2\text{-H})_6$ (**1**). All the stationary points were optimized by TPSSTPSS functional[7], which has been successfully used for the calculations of trinuclear titanium complexes[1,8]. In the geometrical optimizations, the 6-31G(d) basis set was considered for C, H, and N atoms and the Stuttgart/Dresden effective core potentials (ECP) as well as the associated valence basis sets[9,10] MWB10 and MDF10 were used for the Si and Ti atoms, respectively. The basis set of the Si atom was also augmented by one d polarization function (exponent of 0.284)[11]. Considering that antiferromagnetic coupling singlet ground state may exist for some structures, the combination of the spin unrestricted strategy and scf=nosymm keyword was used in the optimizations. Each optimized structure was subsequently analyzed by harmonic vibration frequencies for characterization of a minimum ($N_{\text{imag}} = 0$) or a transition state ($N_{\text{imag}} = 1$). To obtain more reliable relative energies, the single-point calculations of optimized structures were carried out at the level of TPSSTPSS/BSII. In the BSII, the 6-311G(d,p) basis set was used for C, H, N, and Si atoms and MDF10 basis set together with ECP was considered for Ti atoms. In these single point calculations, the solvation effect of toluene was considered with the CPCM solvation model[12,13] and dispersion correction was included through GD3BJ approach[14]. Actually, it was previously reported that TPSSTPSS also often shows the best performance for the structure and energy calculations for transition-metal-containing systems[15-17]. The stabilities of wave functions were tested by the keyword of stable=opt in this study. All of the structures were optimized without any geometrical symmetry restriction. The calculated activation free energy is well in line with the experimental value (*vide infra*), suggesting that the theoretical strategy adopted here is reliable for such reaction. The relative Gibbs free energies in solution were used for discussion of the mechanism in this study. The relative Gibbs free energies in gas-phase of all stationary points are also provided in Supplementary Table 11 for reference. The results suggest that the solvation effect has little influence on the overall reaction. Triplet state pathway was also checked for some important intermediates and the rate-determining transition state. The results shows that triplet states of the structures such as **1m**, **B**, **2m**, $\mathbf{TS_{CD}}$, **D**, **E**, and **3m** are higher in free energy than their corresponding single states by 6.5, 14.2, 7.7, 12.2, 9.9, 4.0, and 12.4 kcal/mol, respectively. Therefore, the triplet state pathway was

not discussed in the text[8]. All calculations were performed with Gaussian 09 program[18].

Results and Discussion

As shown in Supplementary Fig. 57, the Kohn-Sham orbital analysis revealed that the red parts (same sign) of LUMO of $(Cp'Ti)_3H_7$ and HOMO of pyridine concentrate on the Ti1 atom and the N atom, respectively, suggesting that the N atom of pyridine could coordinate to the Ti1 atom of **1m** to provide good orbital overlap. This led us to model the coordination of pyridine to the Ti1 atom via site 1 or site 2 (Supplementary Fig. 57). The site 1 is at the same side of the Ti1−Ti2−Ti3 plane as the μ_3-H is, while the site 2 is at the opposite site.

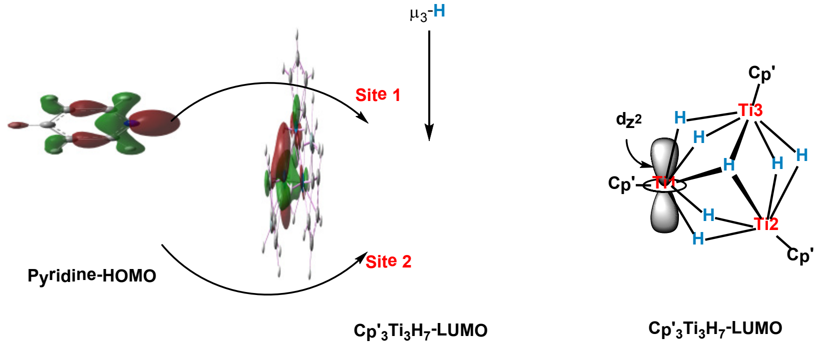

Supplementary Fig. 57 | Orbital analysis of pyridine (HOMO) and $Cp'Ti_3H_7$ (1m, LUMO). Cp′ denotes $C_5H_4SiH_3$ ligand as a model of $C_5Me_4SiMe_3$.

Transformation of 1m + pyridine to 2m + $2H_2$.

This transformation involves several fundamental events, viz., pyridine coordination, H_2 elimination, and C−H bond activation (another molecule of H_2 elimination). As illustrated in Supplementary Fig. 57, two possible sites exist for the coordination of pyridine to Ti1 atom. One site is at the same side of the Ti1−Ti2−Ti3 plane as the μ-H7 is, while the other site is opposite. Both possibilities were successfully located, viz., **A** and **A′**. The result shown in Supplementary Fig. 58 suggests that the former is more favorable than the latter by 6.7 kcal/mol. Then, hydride rearrangement in **A** could occur via transition state $\mathbf{TS_{Aa1}}$ to form intermediate **a1** with a newly formed terminal H (H7) ligand. After the change in coordination fashion of H1 ligand from μ_2 in **a1** to μ_3 in **a2**, the terminal H7 ligand could bind with μ_2-H3 via transition state $\mathbf{TS_{a2B}}$ to release one molecule of H_2 and form intermediate **B**. In **B**, pyridine moiety was partially reduced. During this reduction process, the N−C1 bond was elongated from 1.36 Å in **a2** to 1.45 Å in **B**. In complex **B**, there is an unambiguous agostic interaction between C1−H8 bond and Ti2 atom.

Subsequently, the C1−H bond in [C_5H_4N] moiety was further activated via transition state $\mathbf{TS_{B2m}}$ to give **2m**, which is equivalent to **2** obtained experimentally, accompanied by elimination of another molecule of H_2 (H6−H8). The whole process of the formation of **2m** from **1m** and pyridine overcomes a low energy barrier of 13.8 kcal/mol and is exergonic by 21.2 kcal/mol. Such a low energy barrier is in agreement with experimental observation that the formation of **2** by the reaction of **1** and pyridine could occur rapidly at room temperature.

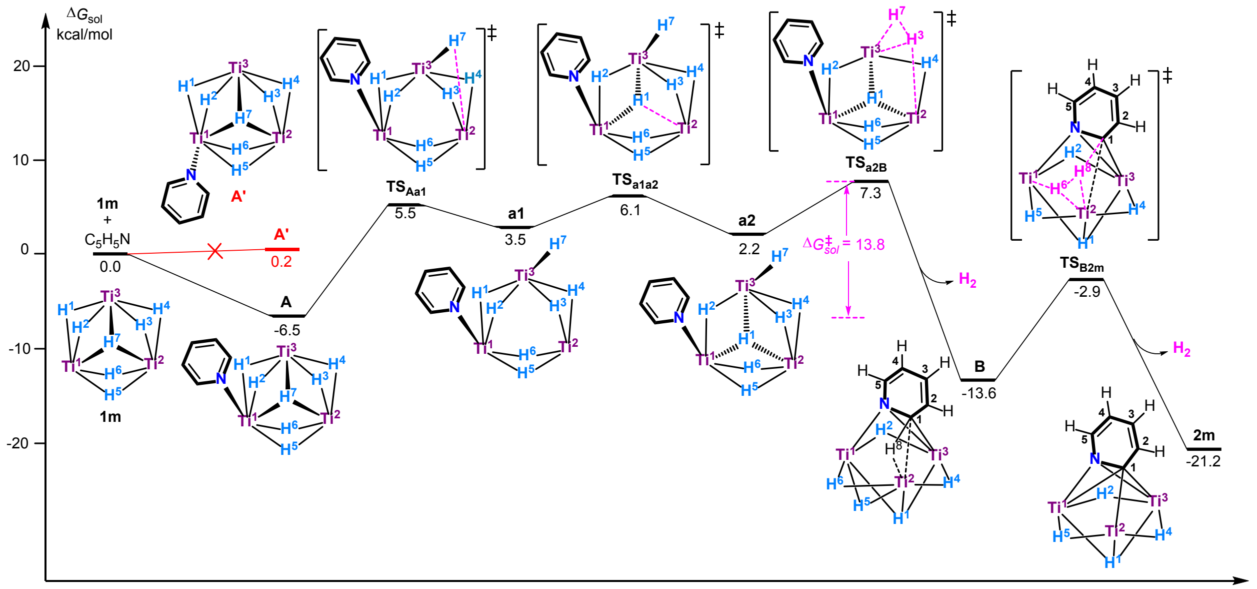

Supplementary Fig. 58 | Computed energy profiles for the transformation of 1m + pyridine to 2m + 2H$_2$. The Gibbs free energies (kcal/mol) are relative to the energy sum of **1m** and pyridine. The $C_5H_4SiH_3$ ligands were omitted for clarity.

Transformation of 2m to (3m + H$_2$).

Such a conversion consists of several chemical events: twofold C−N bond cleavage, C−H bond formation, and H_2 elimination. It is computationally found that the model complex **2m** is difficult to directly cleave the C−N bond or liberate a molecule of H_2. For further transformation, as shown in Supplementary Fig. 59a, the [C_5NH_4] moiety in **2m** needs to change its coordination mode from perpendicular to parallel fashion over the Ti1−Ti2−Ti3 plane via an intermediate **c1**, leading to more active species **C** with higher energy. Starting from **C**, the oxidative addition of C1−N bond of [C_5H_4N] unit to Ti3 atom (*i.e.,* C1−N bond cleavage) and the reductive elimination of the C1 atom and the hydride μ-H4 (*i.e.,* C1−H4 bond formation) could occur.

There are two possible pathways, *i.e.*, the C1−N bond cleavage followed by the C1−H4 bond formation (black line) and the C1−H4 bond formation followed by the C1−N bond cleavage (red line). The energy barriers shown in Supplementary Fig. 59a suggest that the former path (black line) is more favourable than the latter path (red line) by 6.4 kcal/mol (**TS$_{\mathbf{CD}}$** *vs* **TS′$_{\mathbf{CD}}$**). Both the pathways give the same thermodynamically stable complex **E**. During this transformation, the C1−N bond cleavage (via **TS$_{\mathbf{CD}}$**) overcomes an energy barrier of 26.5 kcal/mol. As shown in Supplementary Fig. 59b, the N−H2 bond formation in **E** possibly occur via **TS′$_{\mathbf{EF}}$**, but it needs to overcome a high energy barrier of 30.3 kcal/mol. Alternatively, the rearrangement of hydride ligands in **E** could easily occur via **TS$_{\mathbf{EF}}$** with a low energy barrier of 8.9 kcal/mol, leading to a slightly more stable intermediate **F**.

In **F**, the Ti3−C5 bond could form via the transition state **TS′$_{\mathbf{FG}}$** to give the intermediate **G′**, which could further transform to **3m**, which is equivalent to **3** obtained experimentally, by the sequential oxidative addition of C5−N bond (*i.e.*, C5−N bond cleavage via **TS′$_{\mathbf{GH}}$**) and reductive elimination of H1 and H2 atoms (H_2 elimination via **TS′$_{\mathbf{H3m}}$**). This transformation pathway (red line in the right part of Supplementary Fig. 59b) needs to overcome a high energy barrier of 29.7 kcal/mol. In contrast, the reductive elimination of C1 and H1 atoms in **F** followed by the Ti3−C5 bond formation (via **TS$_{\mathbf{GH}}$**), C5−N bond cleavage (**TS$_{\mathbf{HI}}$**), and H_2 (H1−H2) elimination (via **TS$_{\mathbf{I3m}}$**) could also give the final product **3m** but with a low energy of 19.0 kcal/mol (black line in the right part of Supplementary Fig. 59b). Therefore, the latter path is more likely to work here. The whole process of transformation of **2m** to **3m** is significantly exergonic by 35.9 kcal/mol and the rate-determining step is the cleavage of C1−N bond with an energy barrier of 26.5 kcal/mol, which is well in agreement with experimental result ($\Delta G^{\ddagger}_{(298\ \mathrm{K})}$ = 25.5 kcal/mol) obtained from kinetic studies for the transformation of **2** to **3**.

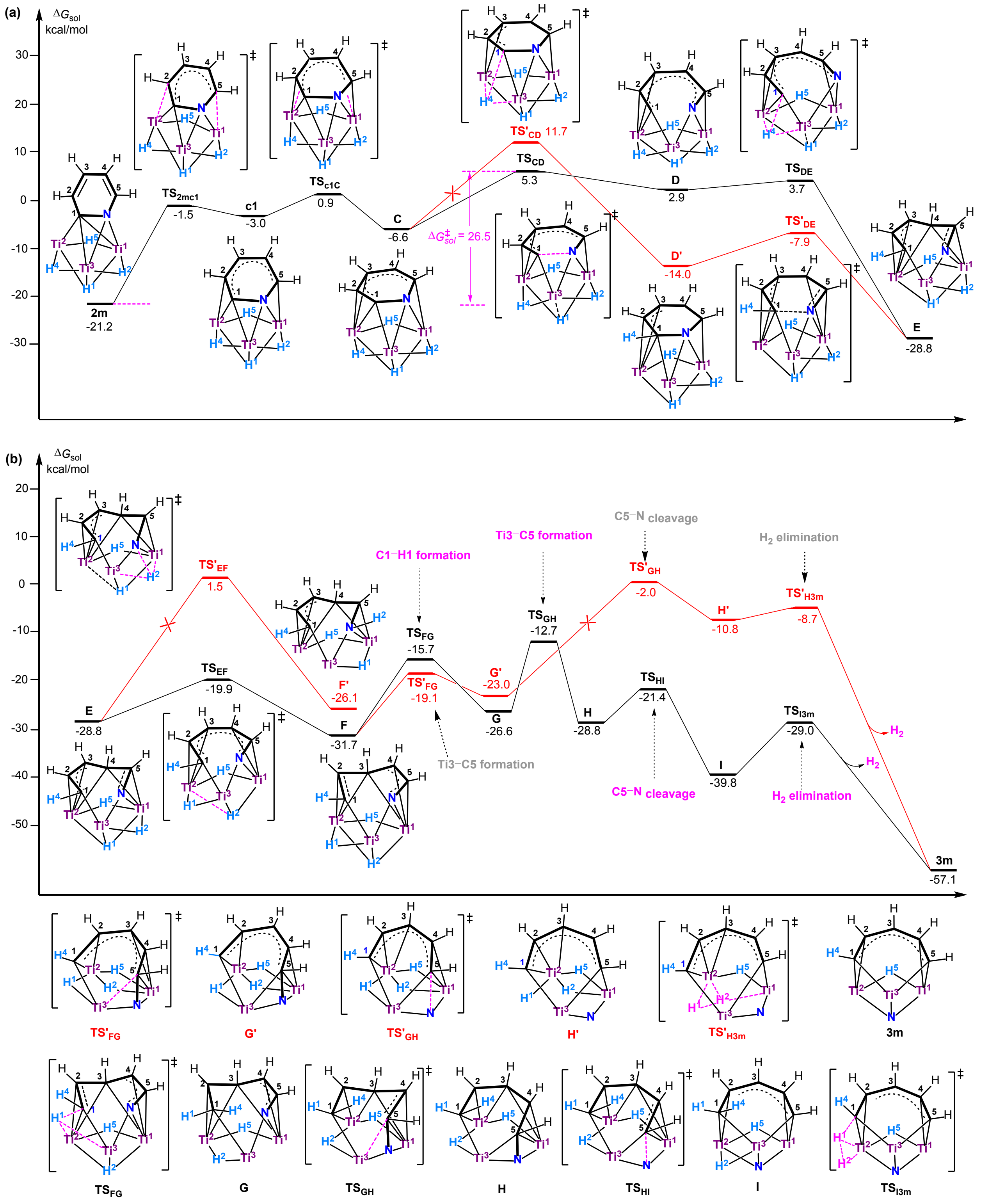

Supplementary Fig. 59 | Computed energy profiles for the reaction of the transformation of 2m to 3m + H_2. The Gibbs free energies (kcal/mol) are relative to the energy sum of **1m** and pyridine. The $C_5H_4SiH_3$ ligands were omitted for clarity.

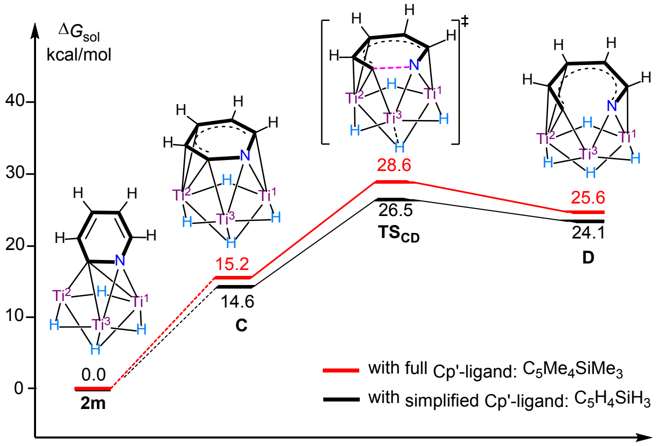

Supplementary Fig. 60 | Energy profiles (in kcal/mol) for the processes of 2m→C→TS_{CD}→D with full Cp′-ligand (red path) and simplified Cp′-ligand (black path). The small energy differences between the two energy profiles indicates that the use of $C_5H_4SiH_3$ as a model of C_5Me_4SiMe is appropriate in this study.

Supplementary Table 11 |. Imaginary frequency of transition states (*IF*, cm^{-1}), Gas-phase zero-point correction ($\Delta\Delta E_{gas}$, a.u.), thermal correction to enthalpy ($\Delta\Delta H_{gas}$, a.u.) and to Gibbs free energy ($\Delta\Delta G_{gas}$, a.u.), single-point energy in gas-phase ($E_{sp\text{-}gas}$, a.u.) and solution ($E_{sp\text{-}sol}$, a.u.), relative Gibbs free energy in gas-phase (ΔG_{gas}, a.u.) and solution (ΔG_{sol}, kcal/mol)

	IF	$\Delta\Delta E_{gas}$	$\Delta\Delta H_{gas}$	$\Delta\Delta G_{gas}$	$E_{sp\text{-}gas}$	$E_{sp\text{-}sol}$	ΔG_{gas}	ΔG_{sol}
**H_2**	/	0.01017	0.01348	-0.00131	-1.17964	-1.17973		/
**Py**	/	0.08774	0.09304	0.06028	-248.40098	-248.40371		/
**1m**	/	0.34958	0.37721	0.29010	-1632.42555	-1632.43132	0.0	0.0
**A'**	/	0.43985	0.47338	0.37356	-1880.84926	-1880.85784	0.3	0.2
**A**	/	0.44097	0.47412	0.37520	-1880.86207	-1880.87028	-6.7	-6.5
**TS_{Aa1}**	-110.05	0.43980	0.47258	0.37553	-1880.84209	-1880.85137	6.0	5.5
**a1**	/	0.44025	0.47372	0.37371	-1880.84344	-1880.85284	4.0	3.5
**TS_{a1a2}**	-167.18	0.43932	0.47240	0.37422	-1880.84061	-1880.84908	6.1	6.1
**a2**	/	0.43931	0.47297	0.37299	-1880.84605	-1880.85407	1.9	2.2
**TS_{a2B}**	-817.72	0.43654	0.47037	0.36876	-1880.83428	-1880.84180	6.7	7.3
**B**	/	0.42477	0.45650	0.36128	-1879.68125	-1879.68648	-15.5	-13.6

**TS_{B2m}**	-539.12	0.41952	0.45154	0.35609	-1879.65925	-1879.66433	-5.0	-2.9
**2m**	/	0.40569	0.43757	0.34177	-1878.49203	-1878.49808	-22.6	-21.2
**TS_{2mc1}**	-72.17	0.40314	0.43494	0.34026	-1878.45941	-1878.46525	-3.1	-1.5
**c1**	/	0.40282	0.43506	0.33919	-1878.46107	-1878.46652	-4.8	-3.0
**TS_{c1C}**	-110.19	0.40214	0.43365	0.33970	-1878.45531	-1878.46082	-0.9	0.9
**C**	/	0.40408	0.43571	0.34220	-1878.46923	-1878.47536	-8.0	-6.6
**TS_{CD}**	-259.22	0.40160	0.43312	0.33934	-1878.44748	-1878.45341	3.8	5.3
**D**	/	0.40124	0.43350	0.33817	-1878.45023	-1878.45612	1.4	2.9
**TS_{DE}**	-329.10	0.40044	0.43214	0.33862	-1878.44938	-1878.45526	2.2	3.7
**E**	/	0.40652	0.43853	0.34371	-1878.50604	-1878.51216	-30.2	-28.8
**TS'_{CD}**	-842.97	0.40143	0.43272	0.33989	-1878.43844	-1878.44382	9.9	11.7
**D'**	/	0.40688	0.43855	0.34508	-1878.48420	-1878.48990	-15.6	-14.0
**TS'_{DE}**	-378.47	0.40442	0.43569	0.34359	-1878.47353	-1878.47873	-9.8	-7.9
**TS_{EF}**	-122.08	0.40520	0.43672	0.34324	-1878.49183	-1878.49759	-21.5	-19.9
**F**	/	0.40725	0.43892	0.34568	-1878.51330	-1878.51877	-33.5	-31.7
**TS'_{EF}**	-1115.67	0.40453	0.43625	0.34326	-1878.45822	-1878.46345	-0.4	1.5
**F'**	/	0.41011	0.44203	0.34817	-1878.50609	-1878.51233	-27.4	-26.1
**TS'_{FG}**	-137.81	0.40520	0.43664	0.34367	-1878.49018	-1878.49668	-20.2	-19.1
**G'**	/	0.40500	0.43691	0.34221	-1878.49469	-1878.50147	-24.0	-23.0
**TS'_{GH}**	-151.75	0.40287	0.43456	0.34115	-1878.46104	-1878.46686	-3.5	-2.0
**H'**	/	0.40442	0.43683	0.34181	-1878.47439	-1878.48152	-11.5	-10.8
**TS'_{H3m}**	-884.62	0.40192	0.43447	0.33795	-1878.46826	-1878.47440	-10.1	-8.7
**TS_{FG}**	-527.50	0.40464	0.43623	0.34308	-1878.48540	-1878.49069	-17.6	-15.7
**G**	/	0.40900	0.44092	0.34701	-1878.50601	-1878.51189	-28.1	-26.6
**TS_{GH}**	-347.54	0.40622	0.43786	0.34497	-1878.48249	-1878.48771	-14.6	-12.7
**H**	/	0.40750	0.43927	0.34557	-1878.50897	-1878.51406	-30.8	-28.8
**TS_{HI}**	-313.89	0.40659	0.43828	0.34458	-1878.49590	-1878.50125	-23.3	-21.4
**I**	/	0.40767	0.43987	0.34572	-1878.52674	-1878.53171	-41.9	-39.8
**TS_{I3m}**	-1167.47	0.40363	0.43531	0.34105	-1878.50436	-1878.50977	-30.8	-29.0
**3m**	/	0.38818	0.41994	0.32436	-1877.35127	-1877.35681	-58.7	-57.1

Optimized Cartesian Coordinates for All of the Stationary Points

H_2

H	1.319436000	0.064935000	0.000000000
H	0.579265000	0.064935000	0.000000000

Pyridine

C	-1.147187000	-0.723706000	-0.000215000
C	-1.203366000	0.676888000	-0.000121000
C	0.000426000	1.392141000	0.000082000
C	1.203783000	0.676171000	0.000213000
C	1.146745000	-0.724389000	0.000134000
N	-0.000439000	-1.430471000	-0.000098000
H	0.000732000	2.481598000	0.000179000
H	-2.065790000	-1.313267000	-0.000369000
H	-2.164300000	1.189088000	-0.000230000
H	2.165044000	1.187762000	0.000362000
H	2.064983000	-1.314515000	0.000191000

1m

Ti	-0.304276000	1.216262000	0.630612000
Ti	-1.037041000	-1.232595000	0.253900000
Ti	1.348512000	-0.481437000	-0.419237000
C	-0.370398000	3.488277000	0.110383000
C	0.445964000	3.330072000	1.286904000
C	-0.358354000	2.839157000	2.353378000
C	-1.685799000	2.682595000	1.855032000
C	-1.695075000	3.082580000	0.487271000
H	-1.556525000	0.389115000	-0.456568000
H	-1.470033000	0.070662000	1.481652000
H	0.484478000	1.047378000	-1.023985000
H	1.522572000	1.027652000	0.637468000
H	0.416701000	-0.461342000	1.272996000
C	-3.199385000	-1.910720000	-0.300209000
C	-2.280401000	-2.864499000	-0.854420000
C	-1.597631000	-3.522589000	0.210026000
C	-2.083989000	-2.993638000	1.441709000
C	-3.061254000	-2.006633000	1.129803000
H	-0.144615000	-1.066275000	-1.351283000
H	0.596878000	-2.067548000	0.162808000
C	3.564594000	-1.195258000	-0.127684000

C	3.585809000	0.105495000	-0.741769000
C	2.985378000	0.028671000	-2.032581000
C	2.579490000	-1.319385000	-2.243430000
C	2.930363000	-2.066420000	-1.081038000
Si	0.193494000	4.099138000	-1.576319000
Si	-4.354770000	-0.765294000	-1.243239000
Si	4.258186000	-1.665926000	1.552152000
H	2.058157000	-1.702185000	-3.114795000
H	2.726259000	-3.121837000	-0.929177000
H	5.686300000	-2.090144000	1.435116000
H	4.196846000	-0.496585000	2.472319000
H	3.483667000	-2.803359000	2.121541000
H	3.972739000	1.010457000	-0.283663000
H	2.828879000	0.858975000	-2.713563000
H	-2.534827000	2.298770000	2.411002000
H	-2.556826000	3.046320000	-0.172207000
H	0.005433000	5.578492000	-1.676665000
H	1.641613000	3.806049000	-1.759054000
H	-0.606347000	3.454524000	-2.652095000
H	1.511658000	3.528799000	1.341509000
H	-0.016401000	2.595198000	3.354655000
H	-0.823571000	-4.274834000	0.100220000
H	-2.106207000	-3.032608000	-1.912637000
H	-5.652095000	-1.456173000	-1.514031000
H	-4.640359000	0.445850000	-0.425886000
H	-3.751729000	-0.380862000	-2.547422000
H	-3.599073000	-1.402053000	1.853313000
H	-1.744470000	-3.268569000	2.435816000

A

Ti	-1.054212000	1.041865000	0.834897000
Ti	-1.135367000	-1.480977000	0.058716000
Ti	0.939211000	0.081019000	-0.748578000
C	-2.169293000	3.091950000	0.731313000
C	-1.019974000	3.235290000	1.586800000
C	-1.187762000	2.419739000	2.747218000
C	-2.443339000	1.759149000	2.623512000
C	-3.040321000	2.164576000	1.396741000
H	-2.215640000	0.010571000	-0.192535000
H	-1.561373000	-0.565662000	1.627461000
H	-0.475862000	1.188038000	-0.903947000

H	0.802978000	1.436033000	0.502551000
H	0.163661000	-0.395529000	0.887118000
C	-3.117937000	-2.656878000	-0.409105000
C	-2.065871000	-3.244910000	-1.184850000
C	-1.101774000	-3.811496000	-0.301139000
C	-1.536656000	-3.586955000	1.039092000
C	-2.771726000	-2.877887000	0.969278000
H	-0.520861000	-0.682143000	-1.486972000
H	0.693583000	-1.732134000	-0.458809000
C	2.966663000	0.667883000	-1.948746000
C	2.028736000	1.739379000	-2.082898000
C	0.886693000	1.270666000	-2.794100000
C	1.094352000	-0.106941000	-3.092991000
C	2.358257000	-0.485554000	-2.554130000
Si	-2.462397000	3.925277000	-0.919902000
Si	-4.618391000	-1.738148000	-1.059733000
Si	4.722368000	0.849003000	-1.317272000
H	0.404538000	-0.755207000	-3.620065000
H	2.801090000	-1.474793000	-2.623576000
H	5.610105000	1.291306000	-2.432653000
H	4.782861000	1.883099000	-0.246028000
H	5.250013000	-0.443552000	-0.798646000
H	2.158687000	2.746778000	-1.699626000
H	0.009514000	1.853461000	-3.048512000
H	-2.854878000	1.036137000	3.320136000
H	-3.981126000	1.791837000	1.003828000
H	-3.200064000	5.215792000	-0.751503000
H	-1.158239000	4.239593000	-1.572170000
H	-3.271079000	3.048088000	-1.810504000
H	-0.148887000	3.845981000	1.367349000
H	-0.485459000	2.314182000	3.568209000
H	-0.180179000	-4.301879000	-0.599278000
H	-1.995145000	-3.228016000	-2.268607000
H	-5.724167000	-2.698344000	-1.368997000
H	-5.123446000	-0.785373000	-0.032874000
H	-4.283645000	-1.013530000	-2.316230000
H	-3.343702000	-2.526146000	1.822180000
H	-1.019790000	-3.889367000	1.944265000
C	3.080842000	-1.760107000	0.764924000
C	3.968600000	-2.228415000	1.735989000
C	4.361518000	-1.368123000	2.768398000

C	3.850540000	−0.065692000	2.778086000
C	2.963330000	0.324637000	1.770333000
N	2.570257000	−0.504468000	0.773356000
H	5.047285000	−1.704449000	3.544171000
H	2.736073000	−2.397207000	−0.044689000
H	4.337265000	−3.250238000	1.677603000
H	4.124719000	0.645698000	3.554101000
H	2.526060000	1.318600000	1.747330000

A′

Ti	−0.755665000	0.666294000	1.308839000
Ti	−1.495420000	−0.798325000	−0.622689000
Ti	1.042751000	−0.835508000	0.094326000
C	−0.411398000	2.730938000	2.370832000
C	−0.189129000	1.680179000	3.332187000
C	−1.411063000	0.983895000	3.561888000
C	−2.405959000	1.587896000	2.739576000
C	−1.798215000	2.651185000	2.011897000
H	−1.377493000	1.043634000	−0.423000000
H	−2.328246000	−0.126197000	0.845481000
H	0.712539000	1.027408000	0.315892000
H	0.792067000	−0.207431000	1.768641000
H	−0.687775000	−1.236324000	1.071172000
C	−3.215149000	−0.338080000	−2.152397000
C	−2.284520000	−1.225262000	−2.788490000
C	−2.282765000	−2.469641000	−2.090962000
C	−3.216650000	−2.379782000	−1.016540000
C	−3.783177000	−1.074095000	−1.053640000
H	0.032028000	−0.284225000	−1.415262000
H	−0.122548000	−2.015649000	−0.625155000
C	1.650820000	−2.802130000	1.266043000
C	2.563705000	−1.792192000	1.724097000
C	3.415914000	−1.419709000	0.655102000
C	3.069430000	−2.214280000	−0.478715000
C	1.996598000	−3.058166000	−0.108122000
Si	0.849634000	3.962179000	1.744258000
Si	−3.588326000	1.437256000	−2.631507000
Si	0.337546000	−3.675772000	2.284549000
H	3.531645000	−2.161771000	−1.459479000
H	1.506642000	−3.780625000	−0.753010000
H	0.916100000	−4.913964000	2.896400000

H	-0.127813000	-2.797101000	3.391296000
H	-0.806368000	-4.086728000	1.426327000
H	2.586603000	-1.375932000	2.725761000
H	4.193300000	-0.662553000	0.691847000
H	-3.440704000	1.272897000	2.655692000
H	-2.297475000	3.273402000	1.275182000
H	0.817821000	5.225581000	2.544941000
H	2.224236000	3.394919000	1.877664000
H	0.594425000	4.319943000	0.321632000
H	0.764812000	1.447965000	3.796340000
H	-1.552383000	0.132752000	4.220432000
H	-1.663507000	-3.329657000	-2.326180000
H	-1.664497000	-0.980645000	-3.645508000
H	-4.686863000	1.485237000	-3.647192000
H	-4.039931000	2.209640000	-1.441700000
H	-2.386355000	2.074850000	-3.236181000
H	-4.510053000	-0.683437000	-0.348596000
H	-3.433592000	-3.153585000	-0.286912000
C	2.269608000	0.205902000	-2.723590000
C	2.951897000	0.993687000	-3.654746000
C	3.698471000	2.086887000	-3.199243000
C	3.733685000	2.344470000	-1.823919000
C	3.024366000	1.505089000	-0.960297000
N	2.301242000	0.447944000	-1.393460000
H	4.235358000	2.724851000	-3.899175000
H	1.660752000	-0.640860000	-3.031615000
H	2.887264000	0.752841000	-4.713695000
H	4.292191000	3.185078000	-1.417894000
H	3.007673000	1.679418000	0.112664000

TS_{Aa1}

Ti	-1.082655000	1.027543000	0.864854000
Ti	-1.087152000	-1.483012000	0.176346000
Ti	0.863183000	0.086834000	-0.740040000
C	-2.025936000	3.146135000	0.518614000
C	-1.096935000	3.264707000	1.611566000
C	-1.561184000	2.475336000	2.698626000
C	-2.780797000	1.852698000	2.303871000
C	-3.067248000	2.263822000	0.970474000
H	-2.195334000	0.012363000	-0.131347000
H	-1.646768000	-0.494321000	1.649641000

H	-0.555512000	1.246958000	-0.851998000
H	0.688959000	1.369578000	0.625272000
H	-0.045693000	-1.581226000	1.547525000
C	-3.060913000	-2.622943000	-0.400159000
C	-1.993841000	-3.182928000	-1.176119000
C	-1.059047000	-3.801048000	-0.296677000
C	-1.532785000	-3.642888000	1.039811000
C	-2.755472000	-2.916893000	0.975345000
H	-0.563350000	-0.758002000	-1.439795000
H	0.673735000	-1.691392000	-0.388328000
C	2.980474000	0.289069000	-1.993658000
C	2.289212000	1.544890000	-2.006654000
C	1.053901000	1.384160000	-2.700698000
C	0.950848000	0.021153000	-3.098964000
C	2.120803000	-0.655132000	-2.644897000
Si	-1.912168000	4.013731000	-1.141370000
Si	-4.547679000	-1.680596000	-1.044294000
Si	4.756182000	0.002660000	-1.467110000
H	0.124711000	-0.423872000	-3.640880000
H	2.331679000	-1.710504000	-2.789940000
H	5.640458000	-0.008922000	-2.669625000
H	5.207452000	1.110465000	-0.578097000
H	4.925812000	-1.300891000	-0.765898000
H	2.657554000	2.476972000	-1.586890000
H	0.320027000	2.159798000	-2.885180000
H	-3.371552000	1.166517000	2.901188000
H	-3.922642000	1.942800000	0.385125000
H	-2.683361000	5.293911000	-1.117956000
H	-0.489437000	4.345312000	-1.435581000
H	-2.469580000	3.159598000	-2.225166000
H	-0.180948000	3.847419000	1.597276000
H	-1.053190000	2.334930000	3.649467000
H	-0.132493000	-4.282866000	-0.592787000
H	-1.894196000	-3.114772000	-2.255379000
H	-5.650965000	-2.622657000	-1.410589000
H	-5.072270000	-0.762725000	0.005981000
H	-4.191912000	-0.905326000	-2.265060000
H	-3.347511000	-2.603914000	1.830051000
H	-1.046282000	-3.998931000	1.940410000
C	2.695105000	-1.625973000	1.317357000
C	3.581751000	-1.895417000	2.363861000

C	4.211410000	-0.831980000	3.019111000
C	3.935644000	0.470357000	2.584436000
C	3.046459000	0.654862000	1.523391000
N	2.418880000	-0.368400000	0.891654000
H	4.897679000	-1.011340000	3.845057000
H	2.162344000	-2.417719000	0.801787000
H	3.759289000	-2.927498000	2.658548000
H	4.398769000	1.336301000	3.052685000
H	2.800745000	1.649679000	1.160428000

a1

Ti	-1.140299000	1.066873000	0.780242000
Ti	-0.867529000	-1.562637000	0.433387000
Ti	0.874576000	0.121241000	-0.726935000
C	-2.420738000	2.943870000	0.187088000
C	-1.458706000	3.372344000	1.166623000
C	-1.730765000	2.712355000	2.396589000
C	-2.862846000	1.868222000	2.208677000
C	-3.285566000	2.009070000	0.856222000
H	-2.155540000	-0.240101000	0.086668000
H	-1.249159000	-0.385028000	1.851485000
H	-0.688659000	1.068288000	-0.972701000
H	0.559613000	1.577414000	0.412064000
H	0.151944000	-1.783639000	1.794528000
C	-2.782830000	-2.811020000	-0.115237000
C	-1.697105000	-3.315584000	-0.901193000
C	-0.719034000	-3.875930000	-0.031151000
C	-1.188894000	-3.741756000	1.310096000
C	-2.447931000	-3.081804000	1.258653000
H	-0.446819000	-0.969245000	-1.255528000
H	0.906437000	-1.591594000	-0.100791000
C	2.910418000	0.003259000	-2.083484000
C	2.415054000	1.353194000	-2.087091000
C	1.142529000	1.374732000	-2.722874000
C	0.817530000	0.038743000	-3.094027000
C	1.898507000	-0.798884000	-2.698326000
Si	-2.546541000	3.530791000	-1.591598000
Si	-4.337701000	-1.975516000	-0.744740000
Si	4.606960000	-0.600596000	-1.563233000
H	-0.102443000	-0.285460000	-3.567567000
H	1.934934000	-1.877012000	-2.821477000

H	5.532331000	-0.591905000	-2.734959000
H	5.190449000	0.284208000	-0.516378000
H	4.526354000	-1.998676000	-1.053646000
H	2.938300000	2.222016000	-1.697634000
H	0.515093000	2.247114000	-2.868562000
H	-3.302404000	1.211026000	2.951356000
H	-4.115748000	1.479298000	0.400828000
H	-3.511313000	4.667149000	-1.699907000
H	-1.216137000	4.019661000	-2.052160000
H	-3.022167000	2.434314000	-2.477621000
H	-0.645278000	4.068919000	0.988215000
H	-1.148231000	2.800941000	3.310420000
H	0.231806000	-4.303174000	-0.333842000
H	-1.616038000	-3.249318000	-1.981751000
H	-5.373288000	-2.995552000	-1.099394000
H	-4.916750000	-1.100845000	0.313980000
H	-4.056905000	-1.173089000	-1.967270000
H	-3.045545000	-2.795814000	2.119415000
H	-0.677857000	-4.077193000	2.204445000
C	2.874681000	-0.997703000	1.573665000
C	3.831251000	-0.970197000	2.593516000
C	4.381587000	0.253902000	2.986436000
C	3.953674000	1.413628000	2.327541000
C	3.002986000	1.302228000	1.312075000
N	2.454924000	0.119962000	0.929455000
H	5.121777000	0.305086000	3.783180000
H	2.395988000	-1.919346000	1.261600000
H	4.124314000	-1.902785000	3.070840000
H	4.346761000	2.393909000	2.588886000
H	2.640392000	2.178675000	0.781035000

TS$_{a1a2}$

Ti	-1.139401000	-1.155095000	-0.555625000
Ti	-1.217658000	1.523560000	-0.205148000
Ti	0.858505000	-0.002587000	0.749258000
C	-1.487887000	-3.486579000	-0.597202000
C	-0.899359000	-3.077255000	-1.847371000
C	-1.814063000	-2.235106000	-2.542328000
C	-2.981142000	-2.104917000	-1.735589000
C	-2.780931000	-2.867964000	-0.551900000
H	-2.407517000	0.101525000	-0.250797000

H	-0.884995000	0.277961000	-1.606945000
H	-0.256406000	-1.454482000	1.002397000
H	0.662820000	-1.223510000	-0.668696000
H	-0.232505000	2.158751000	-1.452354000
C	-3.320816000	2.359712000	0.441926000
C	-2.352185000	2.839358000	1.380610000
C	-1.437279000	3.691909000	0.700656000
C	-1.832504000	3.766082000	-0.669884000
C	-2.980967000	2.944368000	-0.830275000
H	-0.814048000	0.470371000	1.261068000
H	0.458698000	1.750252000	0.487805000
C	3.045522000	-0.153844000	1.864981000
C	2.293898000	-1.331838000	2.157547000
C	1.124335000	-0.966160000	2.890895000
C	1.124389000	0.447876000	3.044222000
C	2.286915000	0.950081000	2.390913000
Si	-0.719201000	-4.604948000	0.698137000
Si	-4.751263000	1.200453000	0.793049000
Si	4.777048000	-0.115115000	1.150139000
H	0.360833000	1.038721000	3.537022000
H	2.564877000	1.998002000	2.324541000
H	5.781977000	-0.138501000	2.254049000
H	5.001529000	-1.319942000	0.301293000
H	5.012626000	1.117239000	0.347522000
H	2.565809000	-2.344965000	1.874686000
H	0.362861000	-1.647226000	3.253758000
H	-3.850579000	-1.497453000	-1.963182000
H	-3.480172000	-2.930759000	0.278633000
H	-1.064555000	-6.035815000	0.438911000
H	0.764671000	-4.480031000	0.667126000
H	-1.230473000	-4.248708000	2.051414000
H	0.089817000	-3.357807000	-2.195755000
H	-1.643415000	-1.753481000	-3.499423000
H	-0.574179000	4.179592000	1.142302000
H	-2.300562000	2.566575000	2.430196000
H	-5.927790000	1.963233000	1.314845000
H	-5.177887000	0.516959000	-0.460401000
H	-4.381036000	0.187307000	1.820706000
H	-3.498328000	2.757002000	-1.767145000
H	-1.345627000	4.344094000	-1.445934000
C	2.512722000	1.957396000	-1.196771000

C	3.337966000	2.373696000	-2.244610000
C	3.920432000	1.414072000	-3.079631000
C	3.663415000	0.063781000	-2.815200000
C	2.837734000	-0.270901000	-1.739136000
N	2.254028000	0.652162000	-0.934870000
H	4.556566000	1.709138000	-3.912448000
H	2.016486000	2.670188000	-0.547307000
H	3.502078000	3.437447000	-2.402611000
H	4.092921000	-0.727306000	-3.426175000
H	2.612157000	-1.306870000	-1.501359000

a2

Ti	-0.832878000	1.126800000	0.727932000
Ti	-1.402790000	-1.423527000	0.031144000
Ti	1.015025000	-0.108584000	-0.711974000
C	-0.980601000	3.483002000	0.858315000
C	-0.301899000	2.997601000	2.033439000
C	-1.202907000	2.204834000	2.797499000
C	-2.446194000	2.175821000	2.102699000
C	-2.312039000	2.958978000	0.918602000
H	-2.330182000	0.114027000	0.423991000
H	-0.770586000	-0.507972000	1.555458000
H	0.288878000	1.584281000	-0.654023000
H	0.949905000	0.770816000	0.940023000
H	-0.511766000	-2.435207000	1.070626000
C	-3.628064000	-1.754801000	-0.655753000
C	-2.774278000	-2.183721000	-1.721468000
C	-2.005502000	-3.297050000	-1.276915000
C	-2.374439000	-3.582225000	0.071386000
C	-3.361804000	-2.632571000	0.454413000
H	-0.844258000	0.100382000	-0.951153000
H	0.125618000	-1.699892000	-0.946209000
C	3.004124000	0.836777000	-1.616847000
C	1.866565000	1.317210000	-2.362218000
C	1.245591000	0.224804000	-3.029550000
C	1.997396000	-0.949046000	-2.726224000
C	3.070439000	-0.571772000	-1.877001000
Si	-0.278637000	4.600258000	-0.474024000
Si	-4.847030000	-0.328806000	-0.691468000
Si	4.210656000	1.838830000	-0.590488000
H	1.772622000	-1.953544000	-3.070664000

H	3.806701000	-1.248654000	-1.454969000
H	5.320581000	2.377376000	-1.433924000
H	3.524121000	2.989246000	0.057152000
H	4.817714000	0.964415000	0.453991000
H	1.535164000	2.348417000	-2.407555000
H	0.351688000	0.273527000	-3.642192000
H	-3.330078000	1.621670000	2.401084000
H	-3.086003000	3.106289000	0.170519000
H	-0.608254000	6.030757000	-0.192992000
H	1.203925000	4.473752000	-0.525815000
H	-0.858182000	4.252692000	-1.802587000
H	0.737382000	3.190197000	2.281676000
H	-0.974981000	1.682401000	3.720903000
H	-1.247980000	-3.817735000	-1.853518000
H	-2.694274000	-1.710215000	-2.695758000
H	-6.150474000	-0.778663000	-1.271247000
H	-5.108831000	0.155978000	0.692045000
H	-4.333934000	0.784135000	-1.536794000
H	-3.815231000	-2.555691000	1.438989000
H	-1.974307000	-4.375573000	0.690749000
C	2.306331000	-2.769950000	0.395581000
C	2.950034000	-3.678995000	1.233753000
C	3.381218000	-3.252521000	2.497559000
C	3.151529000	-1.921237000	2.857609000
C	2.512796000	-1.068021000	1.952531000
N	2.087599000	-1.472387000	0.730993000
H	3.877048000	-3.940264000	3.180391000
H	1.925114000	-3.071202000	-0.576299000
H	3.093069000	-4.705162000	0.901801000
H	3.459655000	-1.534116000	3.826506000
H	2.311448000	-0.029553000	2.195871000

TS_{a2B}

Ti	-0.814363000	1.024895000	0.919159000
Ti	-1.018337000	-1.680824000	0.226397000
Ti	0.978716000	0.048450000	-0.718392000
C	-2.117473000	2.961805000	1.033578000
C	-0.763770000	3.324099000	1.363672000
C	-0.387504000	2.681084000	2.577063000
C	-1.486111000	1.896997000	3.017374000
C	-2.546111000	2.066638000	2.073864000

H	-1.902199000	-0.422202000	1.248649000
H	0.199602000	-0.925837000	1.360059000
H	-0.051154000	1.551805000	-0.663007000
H	1.049978000	1.039008000	0.855397000
H	0.079106000	-1.926701000	1.618820000
C	-3.056603000	-2.493031000	-0.540587000
C	-2.014483000	-3.116992000	-1.308108000
C	-1.226813000	-3.934330000	-0.444085000
C	-1.770268000	-3.842175000	0.869589000
C	-2.882170000	-2.959146000	0.813849000
H	-0.936884000	-0.087343000	-0.752658000
H	0.521054000	-1.697418000	-0.798552000
C	2.437819000	1.420524000	-2.009116000
C	1.127207000	1.427783000	-2.614311000
C	0.811389000	0.116957000	-3.059679000
C	1.928957000	-0.723235000	-2.760706000
C	2.921083000	0.076759000	-2.138694000
Si	-3.098109000	3.575940000	-0.439097000
Si	-4.392372000	-1.342689000	-1.169850000
Si	3.346063000	2.909350000	-1.324169000
H	1.995055000	-1.787536000	-2.960954000
H	3.878226000	-0.281645000	-1.772875000
H	4.167980000	3.571022000	-2.381771000
H	2.374539000	3.907683000	-0.799545000
H	4.274726000	2.493333000	-0.233075000
H	0.484288000	2.295381000	-2.710536000
H	-0.119009000	-0.194377000	-3.521659000
H	-1.507317000	1.254050000	3.891807000
H	-3.511475000	1.571789000	2.122218000
H	-3.892872000	4.794223000	-0.091482000
H	-2.164744000	3.942018000	-1.542881000
H	-4.049051000	2.532575000	-0.912507000
H	-0.119297000	3.959284000	0.763268000
H	0.586583000	2.739722000	3.052437000
H	-0.349898000	-4.506537000	-0.732217000
H	-1.833595000	-2.964921000	-2.368409000
H	-5.567416000	-2.116882000	-1.680184000
H	-4.875785000	-0.464280000	-0.068639000
H	-3.879411000	-0.512263000	-2.295475000
H	-3.485072000	-2.652482000	1.663505000
H	-1.388775000	-4.337167000	1.756266000

C	2.852195000	-2.222335000	0.502663000
C	3.824600000	-2.850402000	1.286014000
C	4.571398000	-2.085831000	2.189220000
C	4.312290000	-0.711789000	2.266749000
C	3.325647000	-0.158375000	1.447298000
N	2.595847000	-0.891175000	0.569490000
H	5.331492000	-2.547241000	2.817306000
H	2.237866000	-2.779337000	-0.198597000
H	3.979235000	-3.922727000	1.187010000
H	4.860385000	-0.068444000	2.951715000
H	3.084748000	0.899758000	1.482275000

B

Ti	-0.769149000	1.113384000	-0.733404000
Ti	1.578896000	0.236558000	0.040540000
Ti	-0.595371000	-0.982043000	0.781335000
C	-2.457130000	2.739210000	-0.502657000
C	-2.815602000	1.855538000	-1.584373000
C	-1.868539000	1.997125000	-2.636688000
C	-0.889510000	2.940467000	-2.211053000
C	-1.254657000	3.399134000	-0.906505000
H	0.910856000	1.811914000	-0.562465000
H	-1.722891000	0.507343000	0.715243000
H	-1.542840000	-0.567415000	-0.770227000
C	3.375321000	1.034875000	1.329275000
C	3.639070000	-0.347030000	1.011009000
C	3.805050000	-0.479255000	-0.393480000
C	3.643175000	0.810452000	-0.976412000
C	3.391506000	1.736188000	0.077568000
H	0.072159000	0.844352000	0.989178000
H	1.093377000	-0.794143000	1.478238000
C	-2.412806000	-2.455224000	1.046252000
C	-2.411673000	-1.544385000	2.162962000
C	-1.228141000	-1.737776000	2.920577000
C	-0.480435000	-2.786493000	2.294359000
C	-1.212056000	-3.231759000	1.159827000
Si	-3.384145000	2.953026000	1.119454000
Si	3.105889000	1.766860000	3.035353000
Si	-3.729023000	-2.612025000	-0.285438000
H	0.483928000	-3.160035000	2.624238000
H	-0.903233000	-4.008055000	0.466407000

H	-4.760481000	-3.614533000	0.122291000
H	-4.417073000	-1.307198000	-0.485749000
H	-3.115657000	-3.073546000	-1.560441000
H	-3.174614000	-0.798719000	2.363633000
H	-0.926685000	-1.175865000	3.799005000
H	-0.009148000	3.247637000	-2.767111000
H	-0.688929000	4.108331000	-0.310039000
H	-4.276833000	4.149113000	1.047109000
H	-4.232221000	1.755888000	1.371380000
H	-2.430547000	3.153819000	2.243869000
H	-3.661648000	1.175245000	-1.585875000
H	-1.879156000	1.461643000	-3.581229000
H	3.969510000	-1.406595000	-0.932193000
H	3.678072000	-1.159158000	1.730650000
H	4.409910000	2.155141000	3.655247000
H	2.261771000	2.990459000	2.939529000
H	2.455049000	0.768252000	3.928150000
H	3.205301000	2.798056000	-0.049913000
H	3.676062000	1.039119000	-2.036454000
C	1.328177000	-2.720136000	-1.121031000
C	2.029220000	-2.924116000	-2.277566000
C	2.122651000	-1.865524000	-3.255693000
C	1.473077000	-0.683601000	-3.030216000
C	0.687997000	-0.465432000	-1.814110000
N	0.648315000	-1.533803000	-0.836528000
H	2.708806000	-2.014392000	-4.161428000
H	1.252526000	-3.483721000	-0.348949000
H	2.514063000	-3.882976000	-2.449345000
H	1.502434000	0.125607000	-3.760837000
H	-0.392624000	-0.146317000	-2.126049000

TS$_{B2m}$

Ti	-0.780065000	1.086755000	-0.802937000
Ti	1.581613000	0.174053000	0.017986000
Ti	-0.592428000	-1.008766000	0.805585000
C	-2.153758000	2.946746000	-0.439097000
C	-2.835497000	2.024963000	-1.315164000
C	-2.129548000	1.941951000	-2.552920000
C	-0.985792000	2.778174000	-2.449304000
C	-1.006081000	3.399043000	-1.163014000
H	0.924218000	1.770235000	-0.558064000

H	-1.762539000	0.407612000	0.613527000
H	-1.584800000	-0.541353000	-1.066036000
C	3.444159000	0.872616000	1.241716000
C	3.640234000	-0.517829000	0.910874000
C	3.764614000	-0.649757000	-0.498576000
C	3.640755000	0.645263000	-1.073889000
C	3.451825000	1.576716000	-0.011012000
H	0.050663000	0.780201000	0.935288000
H	1.092500000	-0.835068000	1.477791000
C	-2.499571000	-2.320325000	1.212244000
C	-2.287501000	-1.489075000	2.371704000
C	-1.051755000	-1.838038000	2.968005000
C	-0.483755000	-2.909773000	2.202540000
C	-1.378342000	-3.214617000	1.143482000
Si	-2.669686000	3.440213000	1.296949000
Si	3.267020000	1.606825000	2.958705000
Si	-3.977255000	-2.282538000	0.053224000
H	0.472331000	-3.386426000	2.396333000
H	-1.229904000	-3.973098000	0.380732000
H	-5.037493000	-3.220169000	0.533507000
H	-4.560844000	-0.913980000	0.008623000
H	-3.568240000	-2.710256000	-1.313285000
H	-2.944424000	-0.688469000	2.697332000
H	-0.596604000	-1.362027000	3.830759000
H	-0.213093000	2.901540000	-3.202445000
H	-0.246753000	4.076628000	-0.784783000
H	-3.449722000	4.715474000	1.267949000
H	-3.538425000	2.383560000	1.884610000
H	-1.471965000	3.654162000	2.154415000
H	-3.737570000	1.474067000	-1.066889000
H	-2.405169000	1.335012000	-3.408959000
H	3.866812000	-1.581385000	-1.047230000
H	3.664197000	-1.334631000	1.625701000
H	4.603266000	1.980217000	3.515919000
H	2.433591000	2.840301000	2.905945000
H	2.647736000	0.611883000	3.877487000
H	3.311020000	2.646388000	-0.132223000
H	3.655770000	0.876000000	-2.133825000
C	1.031679000	-2.833092000	-1.248408000
C	1.569689000	-3.055972000	-2.490884000
C	1.645855000	-1.983475000	-3.440707000

C	1.217363000	-0.728048000	-3.065507000
C	0.682437000	-0.483805000	-1.756600000
N	0.514433000	-1.595572000	-0.870589000
H	2.061953000	-2.162478000	-4.431235000
H	0.942170000	-3.624038000	-0.506238000
H	1.923169000	-4.052633000	-2.749229000
H	1.291014000	0.115447000	-3.752301000
H	-1.137699000	-0.231623000	-1.910216000

2m

Ti	-0.207481000	1.319710000	0.457071000
Ti	-1.214848000	-1.058104000	0.094027000
Ti	1.033132000	-0.437584000	-1.019226000
C	-0.852384000	3.598416000	0.416682000
C	0.570972000	3.532776000	0.603100000
C	0.855384000	2.910214000	1.853787000
C	-0.386907000	2.568524000	2.460592000
C	-1.429147000	2.983689000	1.580158000
H	-1.831947000	0.547934000	0.674622000
H	1.099752000	1.364481000	-0.797126000
C	-3.422068000	-1.685348000	-0.403540000
C	-2.567523000	-2.822804000	-0.611298000
C	-2.004571000	-3.219362000	0.637346000
C	-2.496138000	-2.336591000	1.640392000
C	-3.361034000	-1.401988000	1.005926000
H	-0.745770000	0.408653000	-1.118109000
H	-0.436593000	-1.452027000	-1.547702000
C	3.329602000	-0.224546000	-1.456734000
C	2.623103000	0.341064000	-2.582083000
C	1.882328000	-0.678293000	-3.227147000
C	2.114831000	-1.898036000	-2.517914000
C	3.013584000	-1.624148000	-1.448659000
Si	-1.768148000	4.355309000	-1.036136000
Si	-4.441268000	-0.775275000	-1.690936000
Si	4.463279000	0.699253000	-0.279736000
H	1.666680000	-2.859700000	-2.748749000
H	3.372123000	-2.347040000	-0.722007000
H	5.887652000	0.571528000	-0.714356000
H	4.111061000	2.146338000	-0.278112000
H	4.353673000	0.147546000	1.098778000
H	2.629093000	1.390080000	-2.860902000

H	1.220370000	-0.551105000	-4.078242000
H	-0.523983000	2.055681000	3.407623000
H	-2.491042000	2.840446000	1.755075000
H	-2.046397000	5.802872000	-0.789448000
H	-0.941040000	4.249793000	-2.270607000
H	-3.070574000	3.662036000	-1.236958000
H	1.315663000	3.884492000	-0.104263000
H	1.845823000	2.707725000	2.249518000
H	-1.309078000	-4.037224000	0.794567000
H	-2.368441000	-3.293408000	-1.569186000
H	-5.775851000	-1.427265000	-1.861884000
H	-4.666174000	0.630300000	-1.255521000
H	-3.747533000	-0.799791000	-3.008239000
H	-3.874578000	-0.585387000	1.503124000
H	-2.229237000	-2.349851000	2.692889000
C	1.265079000	-2.821253000	1.109731000
C	1.859229000	-2.830687000	2.336608000
C	1.895961000	-1.612952000	3.108866000
C	1.336821000	-0.463041000	2.607821000
C	0.685361000	-0.403392000	1.309836000
N	0.690761000	-1.658794000	0.595280000
H	2.372000000	-1.614581000	4.088743000
H	1.204286000	-3.694127000	0.462525000
H	2.301330000	-3.748143000	2.720023000
H	1.376770000	0.449337000	3.200293000

TS_{2mc1}

Ti	0.448754000	1.269028000	0.777615000
Ti	-1.763577000	-0.327307000	0.373767000
Ti	0.606459000	-1.081536000	-0.522679000
C	1.316002000	3.236606000	-0.178155000
C	2.339965000	2.659569000	0.647343000
C	1.911876000	2.678158000	2.004816000
C	0.610393000	3.263034000	2.043676000
C	0.248422000	3.606436000	0.710014000
H	-1.329094000	1.472013000	0.363894000
H	1.722774000	-0.042021000	0.483179000
C	-3.840019000	-0.015198000	-0.668389000
C	-3.661242000	-1.432211000	-0.460696000
C	-3.625217000	-1.690063000	0.936536000
C	-3.779375000	-0.449572000	1.623498000

C	-3.918127000	0.571425000	0.640424000
H	0.504070000	0.715749000	-0.941680000
H	-1.057905000	-0.857313000	-1.201740000
C	2.587961000	-1.770567000	-1.563435000
C	1.749429000	-1.161263000	-2.563383000
C	0.576665000	-1.941651000	-2.718455000
C	0.667232000	-3.060356000	-1.828855000
C	1.906597000	-2.962051000	-1.136268000
Si	1.364522000	3.452656000	-2.043948000
Si	-3.978240000	0.874308000	-2.315128000
Si	4.265882000	-1.169961000	-0.971729000
H	-0.083074000	-3.835319000	-1.703375000
H	2.280835000	-3.662934000	-0.397197000
H	5.354834000	-1.807175000	-1.773285000
H	4.375754000	0.304436000	-1.143102000
H	4.472282000	-1.537709000	0.455703000
H	1.957116000	-0.225541000	-3.072551000
H	-0.257847000	-1.713427000	-3.372250000
H	-0.005318000	3.406485000	2.927368000
H	-0.696062000	4.049044000	0.409507000
H	1.812843000	4.835819000	-2.391419000
H	2.336105000	2.492541000	-2.635448000
H	0.013316000	3.246132000	-2.631564000
H	3.276330000	2.244197000	0.288951000
H	2.472283000	2.298157000	2.853042000
H	-3.456627000	-2.655526000	1.403424000
H	-3.550354000	-2.174594000	-1.244954000
H	-5.406204000	0.955058000	-2.749792000
H	-3.453071000	2.261990000	-2.194761000
H	-3.222051000	0.131854000	-3.361616000
H	-4.020066000	1.632547000	0.847371000
H	-3.768995000	-0.307261000	2.699603000
C	0.399072000	-2.646590000	1.541784000
C	1.364685000	-2.478608000	2.583590000
C	1.442872000	-1.292813000	3.294784000
C	0.574161000	-0.217721000	2.927794000
C	-0.338398000	-0.422636000	1.868660000
N	-0.550700000	-1.676033000	1.292238000
H	2.157160000	-1.175436000	4.108146000
H	0.187383000	-3.633393000	1.131062000
H	2.013199000	-3.321583000	2.814889000

H	0.481273000	0.669735000	3.551026000

c1

Ti	-0.359908000	1.338663000	-0.823049000
Ti	1.699205000	-0.470816000	-0.429448000
Ti	-0.671764000	-1.154698000	0.451076000
C	-0.902336000	3.271611000	0.417436000
C	-2.072323000	2.880052000	-0.320381000
C	-1.809407000	2.990148000	-1.713750000
C	-0.469446000	3.452857000	-1.868097000
C	0.082792000	3.628816000	-0.567735000
H	1.476450000	1.372519000	-0.550238000
H	-1.674185000	0.255634000	-0.143714000
C	3.864990000	-0.343519000	0.503209000
C	3.576844000	-1.731799000	0.235838000
C	3.425654000	-1.908918000	-1.165638000
C	3.606568000	-0.642944000	-1.796887000
C	3.890961000	0.309558000	-0.772981000
H	0.159024000	0.515298000	0.740532000
H	1.037044000	-1.245051000	1.090371000
C	-2.743606000	-1.617822000	1.489506000
C	-1.847351000	-1.064763000	2.474342000
C	-0.760607000	-1.954434000	2.660583000
C	-0.968077000	-3.085373000	1.804560000
C	-2.187403000	-2.882466000	1.105033000
Si	-0.713708000	3.357283000	2.281577000
Si	4.205665000	0.437881000	2.176428000
Si	-4.347029000	-0.851721000	0.882368000
H	-0.298613000	-3.934071000	1.698902000
H	-2.626922000	-3.564116000	0.384263000
H	-5.512058000	-1.466510000	1.588804000
H	-4.347987000	0.606438000	1.183734000
H	-4.526100000	-1.072800000	-0.577625000
H	-1.958511000	-0.094850000	2.949191000
H	0.087742000	-1.798636000	3.317812000
H	0.047669000	3.619761000	-2.809196000
H	1.096180000	3.954712000	-0.354492000
H	-1.010772000	4.735798000	2.777599000
H	-1.674726000	2.423871000	2.931829000
H	0.678422000	3.016673000	2.683989000
H	-2.999592000	2.525016000	0.117364000

H	-2.497449000	2.740260000	-2.514757000
H	3.153487000	-2.832743000	-1.665374000
H	3.467471000	-2.508660000	0.986129000
H	5.639181000	0.256295000	2.554882000
H	3.918674000	1.897841000	2.118280000
H	3.367448000	-0.201892000	3.228455000
H	4.055845000	1.370721000	-0.931804000
H	3.529139000	-0.439135000	-2.859881000
C	-0.791325000	-2.476525000	-1.494308000
C	-1.821931000	-2.094633000	-2.431991000
C	-1.810169000	-0.874619000	-3.072746000
C	-0.756904000	0.059380000	-2.756719000
C	0.223344000	-0.389777000	-1.810383000
N	0.380017000	-1.716757000	-1.348493000
H	-2.568792000	-0.621169000	-3.811874000
H	-0.687649000	-3.527480000	-1.220685000
H	-2.607260000	-2.824798000	-2.621637000
H	-0.479625000	0.811912000	-3.498716000

TS$_{c1C}$

Ti	-0.317531000	1.401383000	-0.629690000
Ti	1.729166000	-0.410251000	-0.569343000
Ti	-0.786158000	-1.407470000	0.131326000
C	-0.735844000	3.099569000	0.951892000
C	-1.999790000	2.785512000	0.344623000
C	-1.952731000	3.117309000	-1.036488000
C	-0.661865000	3.655646000	-1.310460000
C	0.082207000	3.642914000	-0.098668000
H	1.494920000	1.408594000	-0.216116000
H	-1.606893000	0.248568000	0.043865000
C	3.835414000	-0.567046000	0.519711000
C	3.570972000	-1.847853000	-0.070391000
C	3.491080000	-1.701531000	-1.482950000
C	3.728139000	-0.329589000	-1.799697000
C	3.940988000	0.364311000	-0.575337000
H	0.279983000	0.106710000	0.600902000
H	0.977485000	-1.664945000	0.638869000
C	-2.854258000	-1.822591000	1.226647000
C	-1.890477000	-1.512948000	2.253190000
C	-0.883171000	-2.505652000	2.253708000
C	-1.201528000	-3.447902000	1.225972000

C	-2.418490000	-3.044945000	0.614466000
Si	-0.262353000	2.922249000	2.758475000
Si	4.075646000	-0.205692000	2.345087000
Si	-4.432883000	-0.872654000	0.865387000
H	-0.602063000	-4.311971000	0.951064000
H	-2.925370000	-3.567107000	-0.191200000
H	-5.601940000	-1.559866000	1.491862000
H	-4.336113000	0.493799000	1.448522000
H	-4.687930000	-0.779457000	-0.598077000
H	-1.911821000	-0.633971000	2.890048000
H	-0.002165000	-2.526625000	2.886019000
H	-0.304187000	4.002032000	-2.275536000
H	1.109885000	3.973957000	0.014537000
H	-0.639193000	4.150106000	3.523078000
H	-0.983224000	1.771186000	3.370929000
H	1.206827000	2.727326000	2.886865000
H	-2.849228000	2.341249000	0.853004000
H	-2.753112000	2.973335000	-1.755202000
H	3.248490000	-2.485905000	-2.193361000
H	3.408024000	-2.771076000	0.477209000
H	5.513858000	-0.343370000	2.725645000
H	3.647269000	1.185344000	2.658050000
H	3.288113000	-1.171724000	3.162165000
H	4.126180000	1.429421000	-0.478549000
H	3.718792000	0.106268000	-2.792327000
C	-1.037087000	-1.874359000	-1.964991000
C	-2.009123000	-1.018608000	-2.619338000
C	-1.694747000	0.277357000	-2.992711000
C	-0.357137000	0.829797000	-2.810031000
C	0.591336000	0.051908000	-2.098299000
N	0.192246000	-1.267605000	-1.640902000
H	-2.445827000	0.889948000	-3.490993000
H	-1.025257000	-2.947153000	-2.176202000
H	-2.993722000	-1.430848000	-2.836162000
H	-0.047038000	1.696429000	-3.393429000

C

Ti	-0.481874000	1.325818000	-0.710235000
Ti	1.643849000	-0.559084000	-0.453560000
Ti	-0.916349000	-1.284937000	0.058353000
C	-0.526648000	3.071151000	0.855108000

C	-1.853642000	2.911490000	0.323119000
C	-1.841980000	3.229911000	-1.063060000
C	-0.506472000	3.596616000	-1.415274000
C	0.290296000	3.505840000	-0.242256000
H	1.398972000	1.254282000	-0.483025000
H	-1.702767000	0.362658000	0.274358000
C	3.770482000	-0.569306000	0.588016000
C	3.453392000	-1.925047000	0.230343000
C	3.344054000	-2.017049000	-1.183578000
C	3.598154000	-0.725380000	-1.734515000
C	3.861324000	0.159549000	-0.648806000
H	0.193401000	0.158308000	0.628932000
H	0.791872000	-1.725621000	0.634107000
C	-2.877922000	-1.757242000	1.248675000
C	-1.786098000	-1.761982000	2.190739000
C	-0.919452000	-2.835516000	1.875806000
C	-1.444599000	-3.511013000	0.731056000
C	-2.653770000	-2.863780000	0.360745000
Si	0.006006000	2.808136000	2.633339000
Si	4.129110000	0.095548000	2.307479000
Si	-4.342322000	-0.584388000	1.267777000
H	-0.982076000	-4.349909000	0.219323000
H	-3.302743000	-3.151679000	-0.460440000
H	-5.512412000	-1.228048000	1.937867000
H	-3.992670000	0.646232000	2.028105000
H	-4.759043000	-0.222632000	-0.116330000
H	-1.633181000	-1.033851000	2.981385000
H	0.002334000	-3.081931000	2.390750000
H	-0.161173000	3.890857000	-2.401355000
H	1.356943000	3.698898000	-0.190825000
H	-0.230909000	4.044926000	3.439326000
H	-0.779465000	1.705318000	3.253425000
H	1.458925000	2.494124000	2.691493000
H	-2.721308000	2.584857000	0.886576000
H	-2.699202000	3.210569000	-1.727544000
H	3.041273000	-2.896249000	-1.743656000
H	3.291363000	-2.741171000	0.927789000
H	5.498805000	-0.313297000	2.738648000
H	4.064577000	1.582261000	2.286026000
H	3.156289000	-0.439092000	3.301284000
H	4.074841000	1.220144000	-0.737215000

H	3.580577000	-0.465306000	-2.786510000
C	-1.252262000	-1.694334000	-1.983460000
C	-2.001751000	-0.483606000	-2.195765000
C	-1.427104000	0.703483000	-2.749209000
C	0.001800000	0.761768000	-2.822908000
C	0.644403000	-0.282085000	-2.103733000
N	0.158535000	-1.594871000	-1.907223000
H	-2.069539000	1.454361000	-3.201485000
H	-1.675540000	-2.676449000	-2.179099000
H	-3.088642000	-0.550310000	-2.133330000
H	0.554602000	1.581363000	-3.274898000

TS_{CD}

Ti	-0.779961000	1.417739000	-0.466096000
Ti	1.736110000	0.043527000	-0.617660000
Ti	-0.501948000	-1.340358000	-0.167597000
C	-1.336748000	2.678067000	1.429570000
C	-2.566227000	2.334152000	0.766391000
C	-2.590362000	2.949023000	-0.513510000
C	-1.382204000	3.698309000	-0.664855000
C	-0.623464000	3.538403000	0.526180000
H	1.022663000	1.720390000	-0.007669000
H	-1.750078000	-0.002964000	0.165219000
C	3.744429000	-0.224845000	0.660016000
C	3.831400000	-1.081726000	-0.496490000
C	3.845153000	-0.284036000	-1.666819000
C	3.757121000	1.083161000	-1.262666000
C	3.712357000	1.117923000	0.161249000
H	0.146359000	0.227864000	0.594558000
H	1.136283000	-1.326380000	0.561047000
C	-2.362599000	-2.515679000	0.696456000
C	-1.375200000	-2.406858000	1.738705000
C	-0.219593000	-3.134545000	1.353883000
C	-0.466302000	-3.697516000	0.064359000
C	-1.782710000	-3.333334000	-0.330374000
Si	-0.795758000	2.154253000	3.148035000
Si	3.722777000	-0.787534000	2.450892000
Si	-4.092386000	-1.789054000	0.724508000
H	0.244681000	-4.269870000	-0.524544000
H	-2.268886000	-3.619266000	-1.258158000
H	-5.083091000	-2.829192000	1.135431000

H	-4.158776000	-0.674334000	1.708618000
H	-4.488204000	-1.297026000	-0.625184000
H	-1.482723000	-1.825675000	2.649537000
H	0.695619000	-3.230693000	1.926735000
H	-1.098183000	4.290887000	-1.528624000
H	0.351187000	3.976302000	0.714513000
H	-1.302520000	3.119585000	4.170756000
H	-1.348181000	0.811216000	3.477254000
H	0.689660000	2.130970000	3.233707000
H	-3.338457000	1.687568000	1.169191000
H	-3.386022000	2.863041000	-1.246278000
H	3.841387000	-0.647587000	-2.688810000
H	3.829295000	-2.167524000	-0.472735000
H	5.080415000	-0.651188000	3.058697000
H	2.774408000	0.028292000	3.257683000
H	3.330671000	-2.223762000	2.512780000
H	3.623350000	2.015384000	0.765676000
H	3.716931000	1.941863000	-1.924106000
C	-0.462335000	-1.481509000	-2.329822000
C	-1.596381000	-0.598281000	-2.403707000
C	-1.585772000	0.815045000	-2.582424000
C	-0.317255000	1.505899000	-2.645236000
C	0.749189000	0.884654000	-2.025700000
N	0.795777000	-1.145935000	-1.897128000
H	-2.480815000	1.296765000	-2.973033000
H	-0.604044000	-2.498190000	-2.708000000
H	-2.556770000	-1.101097000	-2.534571000
H	-0.218880000	2.451039000	-3.179348000

D

Ti	-0.886474000	1.409699000	-0.398508000
Ti	1.860735000	0.157770000	-0.588604000
Ti	-0.384746000	-1.294585000	-0.277555000
C	-1.834893000	2.328679000	1.546826000
C	-2.911347000	2.049417000	0.630911000
C	-2.746873000	2.846617000	-0.531754000
C	-1.570961000	3.642639000	-0.355412000
C	-1.020242000	3.329254000	0.917811000
H	0.786973000	1.514331000	0.390296000
H	-1.868348000	-0.163312000	-0.349754000
C	3.803234000	-0.059739000	0.799749000

C	4.060628000	-0.751792000	-0.435852000
C	4.050324000	0.182484000	-1.499959000
C	3.810280000	1.480624000	-0.941575000
C	3.675366000	1.332095000	0.462429000
H	-0.505990000	0.125362000	0.868528000
H	1.110353000	-0.974071000	0.680451000
C	-2.227304000	-2.632059000	0.395587000
C	-1.406859000	-2.389926000	1.549529000
C	-0.131895000	-2.985568000	1.341466000
C	-0.140256000	-3.603477000	0.055513000
C	-1.423299000	-3.391191000	-0.522087000
Si	-1.573308000	1.598818000	3.255868000
Si	3.727025000	-0.824226000	2.513243000
Si	-4.017020000	-2.122864000	0.153411000
H	0.702480000	-4.095629000	-0.419488000
H	-1.744902000	-3.744974000	-1.496712000
H	-4.924334000	-3.265556000	0.474718000
H	-4.358042000	-0.996445000	1.063477000
H	-4.268065000	-1.728074000	-1.261191000
H	-1.699619000	-1.816770000	2.422527000
H	0.706777000	-2.954642000	2.027632000
H	-1.166073000	4.354484000	-1.067979000
H	-0.111857000	3.753925000	1.332520000
H	-2.103419000	2.533111000	4.295569000
H	-2.320703000	0.317091000	3.379542000
H	-0.127216000	1.381025000	3.525841000
H	-3.703381000	1.326892000	0.794457000
H	-3.392695000	2.845417000	-1.403696000
H	4.160703000	-0.048686000	-2.554714000
H	4.162052000	-1.827691000	-0.544176000
H	5.052560000	-0.716959000	3.193814000
H	2.717475000	-0.128646000	3.357548000
H	3.383082000	-2.269830000	2.401252000
H	3.451564000	2.131469000	1.162294000
H	3.734242000	2.408355000	-1.498210000
C	-0.067826000	-1.364987000	-2.450279000
C	-1.153813000	-0.461873000	-2.681230000
C	-1.321738000	0.972634000	-2.660903000
C	-0.167290000	1.836070000	-2.534675000
C	0.790614000	1.352431000	-1.695726000
N	1.094986000	-1.163921000	-1.729329000

H	-2.235719000	1.358433000	-3.116562000
H	-0.228244000	-2.365776000	-2.869877000
H	-2.039549000	-0.993074000	-3.042864000
H	-0.113207000	2.786055000	-3.074805000

TS_{DE}

Ti	0.946590000	-1.413838000	-0.435933000
Ti	-1.804157000	-0.092801000	-0.619597000
Ti	0.378432000	1.324945000	-0.194058000
C	1.699240000	-2.421842000	1.537742000
C	2.863468000	-1.974556000	0.819868000
C	2.944261000	-2.670341000	-0.415754000
C	1.841563000	-3.579284000	-0.478440000
C	1.085775000	-3.430590000	0.717292000
H	-0.804570000	-1.859212000	-0.049555000
H	1.807978000	0.157856000	-0.086184000
C	-3.834886000	0.024728000	0.660363000
C	-3.988875000	0.840421000	-0.516199000
C	-3.924693000	0.013852000	-1.666002000
C	-3.745175000	-1.335295000	-1.223578000
C	-3.705572000	-1.327864000	0.196671000
H	0.062015000	-0.285135000	0.730803000
H	-1.192344000	1.123322000	0.660765000
C	2.214029000	2.653455000	0.524017000
C	1.378913000	2.390813000	1.664983000
C	0.110204000	2.992800000	1.454056000
C	0.134366000	3.631719000	0.176478000
C	1.425020000	3.433906000	-0.387085000
Si	1.123358000	-1.851945000	3.229576000
Si	-3.864040000	0.624637000	2.439601000
Si	4.011719000	2.161389000	0.310929000
H	-0.702143000	4.134943000	-0.298381000
H	1.760693000	3.812200000	-1.347474000
H	4.901590000	3.319702000	0.623284000
H	4.351518000	1.055177000	1.246655000
H	4.290310000	1.741448000	-1.091208000
H	1.659013000	1.794356000	2.527302000
H	-0.736425000	2.954460000	2.130162000
H	1.617104000	-4.259877000	-1.293506000
H	0.180607000	-3.976771000	0.962436000
H	1.644744000	-2.766431000	4.291198000

H	1.645662000	-0.486642000	3.511004000
H	-0.362428000	-1.857931000	3.308650000
H	3.552029000	-1.207395000	1.156519000
H	3.705033000	-2.532671000	-1.177257000
H	-3.956397000	0.349223000	-2.697252000
H	-4.065529000	1.923761000	-0.522934000
H	-5.222061000	0.438761000	3.033007000
H	-2.891008000	-0.136484000	3.270783000
H	-3.534645000	2.077178000	2.481719000
H	-3.545119000	-2.198070000	0.826732000
H	-3.649023000	-2.206753000	-1.861996000
C	0.225735000	1.401884000	-2.362366000
C	1.314054000	0.467788000	-2.564985000
C	1.421949000	-0.953928000	-2.649032000
C	0.266061000	-1.827894000	-2.502322000
C	-0.726780000	-1.272974000	-1.734582000
N	-0.995903000	1.174743000	-1.765489000
H	2.333139000	-1.356121000	-3.095304000
H	0.409540000	2.396961000	-2.782706000
H	2.242325000	0.991809000	-2.811227000
H	0.224226000	-2.785507000	-3.024935000

E

Ti	0.867462000	-1.388407000	-0.078487000
Ti	-1.934803000	-0.062801000	-0.620990000
Ti	0.242397000	1.293779000	0.105608000
C	2.662770000	-2.426045000	0.951290000
C	2.043932000	-3.392636000	0.078297000
C	0.732997000	-3.663630000	0.557099000
C	0.520776000	-2.878615000	1.731268000
C	1.703424000	-2.136414000	1.982783000
H	-1.553226000	-2.778701000	-1.121588000
H	1.386710000	0.064490000	0.856004000
C	-4.033774000	-0.090650000	0.584978000
C	-4.089615000	0.883929000	-0.472662000
C	-4.018474000	0.212100000	-1.725329000
C	-3.896485000	-1.181754000	-1.463208000
C	-3.915126000	-1.366883000	-0.050163000
H	-0.509189000	-0.410412000	0.673311000
H	-1.491191000	1.152013000	0.705260000
C	1.864110000	3.040651000	0.284029000

C	1.706497000	2.455120000	1.587122000
C	0.372305000	2.650604000	2.028597000
C	-0.315472000	3.384831000	1.010956000
C	0.596499000	3.625496000	-0.051002000
Si	4.390611000	-1.698890000	0.817792000
Si	-4.092756000	0.273547000	2.424035000
Si	3.449629000	3.162032000	-0.707956000
H	-1.360601000	3.674494000	1.032725000
H	0.362008000	4.140616000	-0.977602000
H	4.188964000	4.412913000	-0.365152000
H	4.337861000	2.003694000	-0.411157000
H	3.147091000	3.192884000	-2.167629000
H	2.476842000	1.914356000	2.127667000
H	-0.060754000	2.286885000	2.954638000
H	-0.397577000	-2.826823000	2.307612000
H	1.843547000	-1.427466000	2.791240000
H	5.410817000	-2.729421000	1.169601000
H	4.668086000	-1.238157000	-0.572684000
H	4.510633000	-0.554613000	1.761104000
H	2.500909000	-3.834789000	-0.801497000
H	0.011308000	-4.331546000	0.100448000
H	-4.005574000	0.683324000	-2.702986000
H	-4.146925000	1.959526000	-0.334114000
H	-5.460074000	0.020119000	2.970764000
H	-3.142139000	-0.599125000	3.172175000
H	-3.752078000	1.702956000	2.666042000
H	-3.817166000	-2.321243000	0.460626000
H	-3.794642000	-1.962709000	-2.209370000
C	0.411949000	1.257433000	-2.008492000
C	1.397731000	0.171144000	-1.838581000
C	1.251765000	-1.218488000	-2.275430000
C	0.130067000	-2.078432000	-2.137673000
C	-0.974744000	-1.858031000	-1.277686000
N	-0.872599000	0.957600000	-1.667923000
H	2.145166000	-1.670183000	-2.713174000
H	0.666423000	2.147998000	-2.591240000
H	2.433827000	0.513944000	-1.928648000
H	0.286340000	-3.076810000	-2.563138000

TS′$_{CD}$

Ti	-0.473562000	1.439527000	-0.566343000

Ti	1.902653000	-0.624966000	-0.716995000
Ti	-0.749989000	-1.241730000	-0.116204000
C	-0.838499000	2.805146000	1.297946000
C	-1.702637000	3.260289000	0.237383000
C	-0.907738000	3.761950000	-0.823938000
C	0.461422000	3.604319000	-0.454952000
C	0.503709000	3.028272000	0.849644000
H	1.408661000	1.057975000	-1.083629000
H	-1.424322000	0.321131000	0.564016000
C	3.523050000	-0.610784000	0.967621000
C	3.665268000	-1.840270000	0.232052000
C	4.026165000	-1.541708000	-1.108997000
C	4.115298000	-0.122405000	-1.237694000
C	3.811468000	0.450594000	0.035337000
H	0.566447000	0.075964000	0.445224000
H	0.969536000	-1.922131000	0.157286000
C	-2.871467000	-2.051492000	0.534193000
C	-2.057608000	-1.945346000	1.721392000
C	-0.975330000	-2.846822000	1.615370000
C	-1.095890000	-3.536358000	0.365644000
C	-2.263661000	-3.064078000	-0.284685000
Si	-1.387949000	2.079442000	2.939956000
Si	3.118403000	-0.434696000	2.791065000
Si	-4.504818000	-1.182902000	0.225990000
H	-0.395994000	-4.268450000	-0.024982000
H	-2.636477000	-3.403424000	-1.245630000
H	-5.592202000	-1.832653000	1.017308000
H	-4.445830000	0.252857000	0.613645000
H	-4.866403000	-1.295112000	-1.216731000
H	-2.230746000	-1.258196000	2.543236000
H	-0.164775000	-2.960027000	2.328031000
H	1.322219000	3.871486000	-1.061084000
H	1.403403000	2.778180000	1.402245000
H	-1.391964000	3.140586000	3.993204000
H	-2.775336000	1.553329000	2.814766000
H	-0.469759000	0.996868000	3.383415000
H	-2.787716000	3.202042000	0.242584000
H	-1.274053000	4.159631000	-1.765055000
H	4.157327000	-2.263533000	-1.910476000
H	3.487854000	-2.834542000	0.629941000
H	4.363646000	-0.536421000	3.610686000

H	2.508548000	0.898446000	3.049573000
H	2.190629000	-1.513505000	3.228379000
H	3.801026000	1.511984000	0.262438000
H	4.337661000	0.423144000	-2.148795000
C	-0.889877000	-1.250307000	-2.186491000
C	-1.760980000	-0.077830000	-2.040686000
C	-1.364540000	1.216134000	-2.574922000
C	0.035420000	1.448295000	-2.766126000
C	0.941277000	0.472593000	-2.262238000
N	0.477491000	-0.874913000	-2.027909000
H	-2.115868000	1.900676000	-2.965027000
H	-1.083811000	-2.102018000	-2.838849000
H	-2.834857000	-0.261742000	-1.992875000
H	0.402454000	2.402629000	-3.140834000

D′

Ti	-0.359698000	1.514432000	-0.423257000
Ti	1.851423000	-0.559560000	-0.735378000
Ti	-0.779389000	-1.179197000	-0.266053000
C	-0.730247000	2.701678000	1.550195000
C	-1.420089000	3.379987000	0.481927000
C	-0.454880000	3.887146000	-0.430511000
C	0.839866000	3.521640000	0.045340000
C	0.671876000	2.803284000	1.258577000
H	1.870863000	0.948193000	-2.731753000
H	-1.439038000	0.343786000	0.530190000
C	3.414925000	-0.914755000	0.955878000
C	3.733324000	-1.790238000	-0.146650000
C	4.125550000	-1.001288000	-1.262975000
C	4.057765000	0.369877000	-0.879041000
C	3.624628000	0.428060000	0.472886000
H	0.554054000	0.086959000	0.432291000
H	0.893525000	-1.949323000	-0.066229000
C	-2.919171000	-1.994880000	0.323794000
C	-2.087126000	-2.043456000	1.501880000
C	-1.040900000	-2.967482000	1.281642000
C	-1.196847000	-3.509480000	-0.032943000
C	-2.352670000	-2.928336000	-0.611156000
Si	-1.508154000	1.888153000	3.050576000
Si	2.919381000	-1.433725000	2.686350000
Si	-4.537446000	-1.061815000	0.152802000

H	-0.524189000	-4.213156000	-0.513074000
H	-2.743117000	-3.143291000	-1.600972000
H	-5.633942000	-1.805110000	0.842522000
H	-4.449915000	0.298584000	0.747398000
H	-4.906896000	-0.953226000	-1.288863000
H	-2.227354000	-1.444421000	2.395711000
H	-0.223637000	-3.182161000	1.962390000
H	1.784519000	3.729666000	-0.448944000
H	1.468347000	2.374714000	1.857576000
H	-1.538931000	2.847528000	4.197705000
H	-2.910933000	1.491837000	2.749980000
H	-0.719621000	0.699129000	3.473954000
H	-2.497245000	3.486422000	0.392204000
H	-0.667559000	4.438114000	-1.341515000
H	4.391142000	-1.376588000	-2.247875000
H	3.666896000	-2.873969000	-0.130468000
H	4.119461000	-1.531103000	3.572695000
H	1.989836000	-0.438587000	3.288221000
H	2.272788000	-2.775348000	2.651585000
H	3.473059000	1.337091000	1.045041000
H	4.269582000	1.222216000	-1.518843000
C	-0.834494000	-1.030569000	-2.349469000
C	-1.756788000	0.074476000	-2.037254000
C	-1.454456000	1.433115000	-2.436649000
C	-0.106585000	1.752842000	-2.638183000
C	0.891183000	0.776825000	-2.269526000
N	0.529776000	-0.674221000	-2.185218000
H	-2.251095000	2.143363000	-2.649639000
H	-1.059836000	-1.829355000	-3.056303000
H	-2.817613000	-0.172263000	-1.980923000
H	0.214898000	2.707734000	-3.047169000

TS′$_{DE}$

Ti	-0.741596000	1.524649000	-0.305383000
Ti	1.767844000	-0.062525000	-0.749742000
Ti	-0.646348000	-1.235470000	-0.411871000
C	-0.919675000	2.413083000	1.849451000
C	-2.127296000	2.750205000	1.144415000
C	-1.812296000	3.612642000	0.064775000
C	-0.398595000	3.824179000	0.079613000
C	0.150166000	3.097287000	1.175211000

H	1.724359000	1.833183000	-2.379821000
H	-1.558241000	0.044662000	0.550800000
C	3.575732000	-0.737649000	0.657698000
C	4.007704000	-0.709160000	-0.712406000
C	3.961971000	0.639670000	-1.180404000
C	3.491939000	1.458597000	-0.109055000
C	3.256179000	0.614764000	1.010976000
H	0.391852000	0.225180000	0.427949000
H	1.126983000	-1.590685000	-0.016259000
C	-2.554351000	-2.424652000	0.275864000
C	-1.534492000	-2.609759000	1.278896000
C	-0.463117000	-3.342081000	0.709451000
C	-0.780474000	-3.604862000	-0.655900000
C	-2.066016000	-3.058139000	-0.916111000
Si	-0.791282000	1.327282000	3.373764000
Si	3.458633000	-2.250998000	1.759685000
Si	-4.218481000	-1.606090000	0.560317000
H	-0.138172000	-4.100486000	-1.377369000
H	-2.582347000	-3.091743000	-1.871287000
H	-5.266589000	-2.642168000	0.806776000
H	-4.148064000	-0.717890000	1.751463000
H	-4.645880000	-0.819881000	-0.632668000
H	-1.578893000	-2.230714000	2.295063000
H	0.461672000	-3.611115000	1.207595000
H	0.162110000	4.424406000	-0.630171000
H	1.198284000	3.066102000	1.453975000
H	-0.787510000	2.171976000	4.607591000
H	-1.961592000	0.410722000	3.449497000
H	0.471626000	0.538971000	3.363475000
H	-3.115169000	2.360665000	1.374012000
H	-2.514086000	4.021350000	-0.654933000
H	4.227126000	0.977108000	-2.178801000
H	4.312962000	-1.572272000	-1.297649000
H	4.733370000	-2.447276000	2.514066000
H	2.357328000	-2.094319000	2.749235000
H	3.225461000	-3.467475000	0.931383000
H	2.857918000	0.934212000	1.969361000
H	3.318374000	2.529285000	-0.151536000
C	-0.649030000	-0.879850000	-2.510495000
C	-1.699032000	0.038846000	-1.946087000
C	-1.660100000	1.456788000	-2.292493000

C	-0.377267000	2.030676000	-2.507946000
C	0.784441000	1.409755000	-2.000570000
N	0.623033000	-0.527416000	-2.175415000
H	-2.556417000	1.962754000	-2.645341000
H	-0.829718000	-1.506600000	-3.388163000
H	-2.714192000	-0.369238000	-1.930715000
H	-0.294131000	3.002041000	-2.997739000

TS_{EF}

Ti	-0.094997000	1.482126000	-0.177935000
Ti	1.860289000	-0.502520000	-0.578704000
Ti	-0.826324000	-1.362076000	0.061349000
C	-1.530503000	3.145026000	0.615113000
C	-0.739212000	3.706579000	-0.445795000
C	0.627848000	3.703578000	-0.043192000
C	0.700501000	3.155547000	1.272592000
C	-0.617038000	2.810980000	1.675766000
H	2.088045000	1.816230000	-2.048904000
H	-1.164943000	0.290711000	0.727773000
C	3.875278000	-0.533378000	0.718841000
C	3.678514000	-1.824500000	0.129701000
C	3.748849000	-1.703025000	-1.288530000
C	4.004402000	-0.333608000	-1.599263000
C	4.080152000	0.380220000	-0.373549000
H	1.394842000	0.854730000	0.619398000
H	0.746360000	-1.067297000	0.885296000
C	-2.900220000	-2.269972000	0.714268000
C	-2.224228000	-1.814609000	1.900155000
C	-1.021803000	-2.556166000	2.062327000
C	-0.940817000	-3.496970000	0.988567000
C	-2.089946000	-3.328407000	0.169100000
Si	-3.398228000	2.961540000	0.666288000
Si	3.873513000	-0.109274000	2.546957000
Si	-4.575500000	-1.735346000	0.060875000
H	-0.119754000	-4.183656000	0.806008000
H	-2.321833000	-3.900442000	-0.723572000
H	-5.668150000	-2.443730000	0.792081000
H	-4.781568000	-0.271902000	0.223936000
H	-4.676814000	-2.099122000	-1.381505000
H	-2.556179000	-1.007120000	2.544564000
H	-0.282268000	-2.412920000	2.842308000

H	1.605859000	2.990569000	1.845863000
H	-0.885510000	2.328667000	2.610261000
H	-4.023930000	4.265956000	1.037639000
H	-3.932992000	2.561697000	-0.665486000
H	-3.774916000	1.951829000	1.692058000
H	-1.120915000	4.072401000	-1.393393000
H	1.470923000	4.026132000	-0.645331000
H	3.602339000	-2.503865000	-2.006496000
H	3.456428000	-2.738798000	0.673117000
H	5.224358000	-0.344489000	3.140465000
H	3.537089000	1.330075000	2.731797000
H	2.894850000	-0.955068000	3.285027000
H	4.222421000	1.452351000	-0.274817000
H	4.093544000	0.088584000	-2.594103000
C	-0.650231000	-1.750236000	-2.058323000
C	-1.545563000	-0.660383000	-2.211740000
C	-1.211562000	0.738926000	-2.222727000
C	0.045949000	1.426184000	-2.378385000
C	1.320477000	1.055789000	-1.843786000
N	0.526049000	-1.506451000	-1.405748000
H	-2.082744000	1.377462000	-2.395019000
H	-0.906432000	-2.757259000	-2.393217000
H	-2.583119000	-0.905972000	-2.450677000
H	-0.056719000	2.397432000	-2.872989000

F

Ti	0.351386000	-1.496791000	0.119451000
Ti	-1.938871000	-0.184723000	-0.401647000
Ti	0.646929000	1.189267000	-0.038458000
C	2.257908000	-2.765107000	0.716625000
C	1.525704000	-3.525531000	-0.251391000
C	0.251342000	-3.844417000	0.294464000
C	0.192127000	-3.325748000	1.626387000
C	1.410939000	-2.655649000	1.880965000
H	-1.529737000	-2.366433000	-2.080033000
H	1.441617000	-0.206131000	0.817689000
C	-4.038994000	0.408208000	0.503457000
C	-4.050469000	0.707812000	-0.908878000
C	-3.965207000	-0.504271000	-1.641046000
C	-3.913169000	-1.576455000	-0.698396000
C	-3.970512000	-1.023240000	0.603671000

H	-1.355685000	-1.555988000	0.745843000
H	-0.485524000	0.077290000	0.879676000
C	2.520063000	2.619082000	0.393037000
C	2.152090000	1.996392000	1.631531000
C	0.832509000	2.396701000	1.980443000
C	0.378052000	3.319212000	0.986856000
C	1.402524000	3.452852000	0.018295000
Si	4.017262000	-2.130752000	0.582219000
Si	-4.203914000	1.620983000	1.921219000
Si	4.185290000	2.506358000	-0.465342000
H	-0.602141000	3.783339000	0.950885000
H	1.355095000	4.078406000	-0.867730000
H	4.903817000	3.811255000	-0.378289000
H	5.007159000	1.455935000	0.192558000
H	4.031422000	2.176487000	-1.912818000
H	2.766394000	1.297864000	2.189422000
H	0.269997000	2.056947000	2.844052000
H	-0.653782000	-3.399358000	2.300855000
H	1.663285000	-2.121203000	2.791832000
H	4.990207000	-3.233794000	0.841614000
H	4.296068000	-1.595107000	-0.780446000
H	4.241160000	-1.064445000	1.596718000
H	1.876415000	-3.800600000	-1.240835000
H	-0.548980000	-4.370579000	-0.217047000
H	-3.924049000	-0.600513000	-2.720889000
H	-4.075025000	1.704843000	-1.337146000
H	-5.639030000	1.873132000	2.252714000
H	-3.541527000	1.076107000	3.141108000
H	-3.584728000	2.930172000	1.566479000
H	-3.915620000	-1.586029000	1.530753000
H	-3.799770000	-2.629428000	-0.936011000
C	-0.258494000	1.855781000	-2.008171000
C	0.898597000	1.123597000	-2.295717000
C	1.201395000	-0.225193000	-1.754122000
C	0.331501000	-1.400417000	-2.091970000
C	-1.020975000	-1.461003000	-1.721674000
N	-1.090649000	1.392796000	-1.035676000
H	2.259314000	-0.459491000	-1.908865000
H	-0.383118000	2.865151000	-2.422386000
H	1.685189000	1.617355000	-2.869831000
H	0.775842000	-2.123198000	-2.785288000

TS′$_{EF}$

Ti	0.776384000	−1.357034000	0.126061000
Ti	−1.918571000	−0.246038000	−0.702978000
Ti	0.224970000	1.253519000	0.073930000
C	2.887488000	−2.254376000	0.715117000
C	2.079391000	−3.305315000	0.153371000
C	0.960993000	−3.544257000	1.004408000
C	1.053766000	−2.637494000	2.100103000
C	2.231497000	−1.860830000	1.929849000
H	−1.555293000	−3.037260000	−0.658418000
H	1.158259000	0.123391000	1.096844000
C	−3.909556000	−0.170453000	0.585085000
C	−3.988866000	0.831779000	−0.448165000
C	−4.038061000	0.189435000	−1.715895000
C	−3.977678000	−1.216286000	−1.492283000
C	−3.919517000	−1.434858000	−0.087291000
H	−0.885056000	−0.037178000	0.811494000
H	−1.471067000	1.627375000	−0.722341000
C	1.713903000	3.081005000	0.340696000
C	1.415714000	2.566019000	1.648314000
C	0.029746000	2.726834000	1.912307000
C	−0.559985000	3.366817000	0.778861000
C	0.469181000	3.579478000	−0.183151000
Si	4.506251000	−1.577467000	0.056126000
Si	−3.840666000	0.134269000	2.435528000
Si	3.399849000	3.203135000	−0.466586000
H	−1.601099000	3.656268000	0.678969000
H	0.332865000	4.046340000	−1.154138000
H	3.930143000	4.596543000	−0.380984000
H	4.354172000	2.290931000	0.219994000
H	3.321863000	2.844629000	−1.913092000
H	2.131927000	2.095384000	2.314302000
H	−0.495436000	2.394834000	2.802240000
H	0.330197000	−2.528548000	2.902995000
H	2.566587000	−1.073470000	2.596324000
H	5.603075000	−2.578720000	0.216177000
H	4.409093000	−1.253842000	−1.397755000
H	4.866538000	−0.350588000	0.816280000
H	2.289592000	−3.834191000	−0.771213000
H	0.177660000	−4.276844000	0.844288000

H	-4.061296000	0.685687000	-2.680820000
H	-3.996584000	1.905117000	-0.281996000
H	-5.207426000	0.055950000	3.035581000
H	-2.990541000	-0.889766000	3.105234000
H	-3.297697000	1.494703000	2.704914000
H	-3.836783000	-2.404503000	0.394853000
H	-3.962639000	-1.985591000	-2.258454000
C	0.296868000	1.221501000	-2.046193000
C	1.239767000	0.106372000	-1.625248000
C	1.025781000	-1.281515000	-2.105435000
C	-0.064859000	-2.144563000	-1.872713000
C	-1.005877000	-2.099444000	-0.781793000
N	-1.033853000	1.072889000	-1.820472000
H	1.808670000	-1.687649000	-2.749528000
H	0.677049000	2.009922000	-2.704278000
H	2.271998000	0.413404000	-1.830105000
H	-0.007275000	-3.080606000	-2.438946000

F′

Ti	0.838683000	-1.468634000	0.111229000
Ti	-1.918723000	-0.388489000	-0.568541000
Ti	0.159560000	1.178192000	0.070469000
C	3.040648000	-2.021142000	0.772605000
C	2.490790000	-3.124034000	0.026503000
C	1.396734000	-3.681487000	0.751573000
C	1.250463000	-2.931197000	1.953203000
C	2.247852000	-1.921807000	1.967823000
H	-1.526615000	-3.194532000	-0.421517000
H	0.868588000	-0.040256000	1.187284000
C	-3.966149000	-0.056547000	0.586150000
C	-3.979757000	0.775387000	-0.585884000
C	-3.973634000	-0.056711000	-1.739777000
C	-3.961983000	-1.415060000	-1.300466000
C	-3.965580000	-1.412008000	0.119229000
H	-1.360393000	0.533752000	0.874305000
H	-1.600439000	1.734520000	-2.124059000
C	1.632692000	3.081840000	0.200160000
C	1.514278000	2.506554000	1.507782000
C	0.161209000	2.596965000	1.937992000
C	-0.582291000	3.250224000	0.906097000
C	0.315310000	3.537342000	-0.156500000

Si	4.546921000	-0.986466000	0.368387000
Si	-3.961726000	0.532495000	2.368283000
Si	3.204237000	3.307174000	-0.794579000
H	-1.647006000	3.459695000	0.922712000
H	0.050691000	4.024889000	-1.091516000
H	3.490262000	4.758685000	-0.995946000
H	4.346636000	2.689854000	-0.069015000
H	3.098444000	2.688352000	-2.150269000
H	2.323744000	2.045383000	2.064843000
H	-0.241503000	2.213572000	2.869578000
H	0.472089000	-3.067107000	2.699195000
H	2.376963000	-1.176537000	2.745353000
H	5.802317000	-1.769018000	0.582263000
H	4.543988000	-0.543431000	-1.056104000
H	4.577376000	0.201022000	1.264945000
H	2.857611000	-3.483563000	-0.930161000
H	0.776276000	-4.514257000	0.437780000
H	-3.967421000	0.276648000	-2.773627000
H	-3.971827000	1.861831000	-0.581869000
H	-5.344252000	0.481101000	2.935414000
H	-3.093344000	-0.333262000	3.213309000
H	-3.492084000	1.944053000	2.431794000
H	-3.923507000	-2.295831000	0.749948000
H	-3.937201000	-2.293725000	-1.937607000
C	0.300807000	1.082497000	-2.033964000
C	1.180394000	-0.046119000	-1.643727000
C	0.895969000	-1.432725000	-2.118008000
C	-0.172755000	-2.289517000	-1.777635000
C	-1.019333000	-2.241083000	-0.608998000
N	-1.059355000	0.942820000	-1.761123000
H	1.618086000	-1.849930000	-2.823443000
H	0.635208000	1.837483000	-2.748547000
H	2.221493000	0.219266000	-1.862999000
H	-0.174161000	-3.225009000	-2.347866000

TS′$_{FG}$

Ti	-0.003755000	1.308384000	0.138382000
Ti	2.145353000	-0.239660000	-0.520379000
Ti	-1.294404000	-1.100578000	0.199795000
C	-1.366969000	3.151174000	0.681749000
C	-0.349263000	3.598112000	-0.222040000

C	0.921965000	3.434071000	0.400392000
C	0.703235000	2.905020000	1.711425000
C	-0.691517000	2.725950000	1.880103000
H	1.764308000	1.913537000	-2.124185000
H	-1.754886000	0.651949000	0.222249000
C	4.238884000	-0.935515000	0.330485000
C	4.198422000	-1.141613000	-1.093210000
C	4.159035000	0.127064000	-1.747467000
C	4.190027000	1.132443000	-0.738531000
C	4.224692000	0.488475000	0.524045000
H	1.571718000	0.765815000	0.889424000
H	-0.364911000	-0.051006000	1.384915000
C	-3.424048000	-1.998044000	0.334108000
C	-3.233019000	-1.155919000	1.490175000
C	-2.189151000	-1.699608000	2.288161000
C	-1.721708000	-2.886887000	1.652291000
C	-2.478224000	-3.080923000	0.462887000
Si	-3.218033000	3.140187000	0.371987000
Si	4.349673000	-2.254966000	1.659600000
Si	-4.719037000	-1.803567000	-1.007559000
H	-0.890297000	-3.500019000	1.987918000
H	-2.362612000	-3.903501000	-0.235466000
H	-6.058887000	-2.246791000	-0.520097000
H	-4.833670000	-0.383798000	-1.444119000
H	-4.346064000	-2.651810000	-2.175737000
H	-3.774040000	-0.239636000	1.700422000
H	-1.784968000	-1.263146000	3.194879000
H	1.471904000	2.644457000	2.430013000
H	-1.171465000	2.285719000	2.748325000
H	-3.775876000	4.516039000	0.542076000
H	-3.523772000	2.692632000	-1.014739000
H	-3.889330000	2.247894000	1.356581000
H	-0.516572000	3.983318000	-1.222234000
H	1.886315000	3.660134000	-0.040775000
H	4.114130000	0.293797000	-2.818451000
H	4.185729000	-2.109467000	-1.589395000
H	5.772344000	-2.504458000	2.039417000
H	3.614002000	-1.824662000	2.881717000
H	3.774671000	-3.533025000	1.152304000
H	4.214143000	0.989101000	1.487465000
H	4.132720000	2.203427000	-0.905376000

C	−0.157804000	−2.071015000	−1.321827000
C	−1.107101000	−1.346527000	−2.085301000
C	−1.206140000	0.074458000	−2.088170000
C	−0.195072000	1.135764000	−2.082695000
C	1.204825000	1.042486000	−1.756500000
N	0.648537000	−1.353705000	−0.435103000
H	−2.184741000	0.439701000	−2.407425000
H	−0.083950000	−3.158848000	−1.354132000
H	−1.915926000	−1.904970000	−2.560649000
H	−0.550908000	2.027379000	−2.607627000

G′

Ti	0.038705000	−1.340455000	−0.138449000
Ti	2.174558000	0.261304000	0.448662000
Ti	−1.440440000	0.953250000	−0.340637000
C	−1.299895000	−3.238847000	−0.557509000
C	−0.294924000	−3.594926000	0.402259000
C	0.986892000	−3.474844000	−0.206722000
C	0.789410000	−3.058406000	−1.559761000
C	−0.604015000	−2.909210000	−1.771853000
H	1.888476000	−1.809803000	2.154351000
H	−1.787756000	−0.798682000	−0.048318000
C	4.220486000	1.126752000	−0.328730000
C	4.104378000	1.396190000	1.082281000
C	4.139355000	0.161252000	1.795483000
C	4.273971000	−0.886601000	0.838015000
C	4.315610000	−0.300312000	−0.454878000
H	1.688322000	−0.881502000	−0.892678000
H	−0.755119000	−0.310001000	−1.530257000
C	−3.476556000	2.104845000	−0.209280000
C	−3.653888000	1.029063000	−1.152046000
C	−2.784743000	1.234649000	−2.257187000
C	−2.052827000	2.436677000	−2.025250000
C	−2.478707000	2.977732000	−0.776081000
Si	−3.157592000	−3.244190000	−0.298337000
Si	4.282917000	2.380283000	−1.722735000
Si	−4.438685000	2.383258000	1.375259000
H	−1.274183000	2.840425000	−2.665786000
H	−2.108792000	3.891693000	−0.321277000
H	−5.802330000	2.914878000	1.081714000
H	−4.594493000	1.112139000	2.137837000

H	-3.721899000	3.381335000	2.219687000
H	-4.317878000	0.179782000	-1.025898000
H	-2.661328000	0.569144000	-3.104470000
H	1.569921000	-2.850675000	-2.282681000
H	-1.068997000	-2.561050000	-2.687926000
H	-3.695270000	-4.628083000	-0.475318000
H	-3.505799000	-2.796933000	1.078886000
H	-3.817092000	-2.368155000	-1.305030000
H	-0.481617000	-3.904489000	1.424456000
H	1.944121000	-3.654198000	0.269293000
H	4.061700000	0.041708000	2.870702000
H	4.009196000	2.381876000	1.531644000
H	5.663925000	2.924257000	-1.884553000
H	3.877413000	1.734232000	-3.001736000
H	3.373546000	3.526708000	-1.432516000
H	4.380801000	-0.843963000	-1.392304000
H	4.290620000	-1.950195000	1.053901000
C	0.069460000	1.824120000	0.955279000
C	-0.909350000	1.344546000	1.881421000
C	-1.084612000	-0.033630000	2.147397000
C	-0.101370000	-1.098782000	2.119657000
C	1.285339000	-0.987113000	1.748477000
N	0.469931000	0.912098000	-0.085024000
H	-2.039623000	-0.309153000	2.599086000
H	0.309622000	2.881675000	0.843215000
H	-1.568181000	2.057858000	2.379904000
H	-0.425979000	-1.975549000	2.686134000

TS′$_{GH}$

Ti	-0.823392000	-1.556585000	-0.061727000
Ti	1.953070000	-0.707579000	0.176935000
Ti	-0.657453000	1.162553000	-0.387205000
C	-3.004009000	-2.301236000	-0.529879000
C	-2.449500000	-3.113023000	0.515542000
C	-1.328267000	-3.826647000	-0.002595000
C	-1.172387000	-3.463695000	-1.374206000
C	-2.195978000	-2.530647000	-1.694885000
H	1.192613000	-3.063431000	1.325320000
H	-1.899296000	-0.083819000	0.185670000
C	4.093636000	0.122671000	-0.387764000

C	4.084597000	-0.135669000	1.027498000
C	3.962348000	-1.538031000	1.243205000
C	3.873963000	-2.166325000	-0.029568000
C	3.949137000	-1.153107000	-1.026353000
H	0.685564000	-1.812964000	-1.043897000
H	-1.029778000	-0.299023000	-1.477967000
C	-1.602914000	3.339764000	0.059201000
C	-2.560210000	2.608336000	-0.718408000
C	-1.987277000	2.291954000	-1.984119000
C	-0.671500000	2.840325000	-2.018654000
C	-0.429224000	3.469234000	-0.766913000
Si	-4.500586000	-1.177664000	-0.406178000
Si	4.228047000	1.784642000	-1.248426000
Si	-1.886540000	4.114994000	1.743558000
H	0.044295000	2.731784000	-2.826492000
H	0.498390000	3.954795000	-0.477111000
H	-2.440047000	5.493319000	1.601794000
H	-2.855994000	3.298322000	2.527293000
H	-0.598334000	4.207272000	2.488366000
H	-3.549673000	2.310500000	-0.385107000
H	-2.460653000	1.715170000	-2.771571000
H	-0.398591000	-3.816216000	-2.045907000
H	-2.321600000	-2.043662000	-2.655683000
H	-5.750054000	-1.952469000	-0.676357000
H	-4.612465000	-0.597003000	0.960913000
H	-4.412048000	-0.088981000	-1.417803000
H	-2.823710000	-3.170816000	1.532384000
H	-0.692806000	-4.509173000	0.550881000
H	3.899064000	-2.031159000	2.207364000
H	4.157705000	0.615069000	1.807951000
H	5.660364000	2.186478000	-1.389803000
H	3.629795000	1.702369000	-2.608438000
H	3.542401000	2.843097000	-0.453105000
H	3.867868000	-1.315753000	-2.097655000
H	3.725389000	-3.226946000	-0.210954000
C	1.154412000	0.850460000	1.393942000
C	-0.145444000	0.789550000	1.931022000
C	-0.833189000	-0.468835000	2.267591000
C	-0.259358000	-1.746605000	2.195028000
C	0.816682000	-2.032972000	1.312407000
N	0.932007000	0.492467000	-0.821465000

H	-1.779869000	-0.359792000	2.797837000
H	1.622405000	1.843265000	1.423869000
H	-0.591012000	1.678579000	2.395201000
H	-0.768957000	-2.541274000	2.749291000

H′

Ti	-1.677341000	-1.169592000	-0.263539000
Ti	1.613074000	-1.241436000	-0.066337000
Ti	-0.048242000	1.066044000	-0.562067000
C	-3.950121000	-0.533489000	-0.174819000
C	-3.813377000	-1.774146000	0.520770000
C	-3.368534000	-2.770765000	-0.393986000
C	-3.238773000	-2.166521000	-1.682444000
C	-3.589831000	-0.796166000	-1.544617000
H	0.165546000	-3.599100000	-0.040106000
H	-1.654792000	0.617062000	0.201049000
C	3.959336000	-0.923860000	-0.173267000
C	3.660293000	-1.257915000	1.193587000
C	3.197159000	-2.599954000	1.251122000
C	3.179609000	-3.112214000	-0.075065000
C	3.643902000	-2.089791000	-0.949294000
H	-0.986740000	-1.985170000	-1.567506000
H	-1.244325000	0.063708000	-1.591462000
C	0.259552000	3.329560000	0.179247000
C	-1.093555000	3.227100000	-0.285124000
C	-1.085459000	2.904692000	-1.673661000
C	0.271872000	2.825054000	-2.096965000
C	1.094758000	3.072402000	-0.965770000
Si	-4.541114000	1.099018000	0.533128000
Si	4.694097000	0.675099000	-0.817194000
Si	0.838905000	3.831425000	1.891343000
H	0.618048000	2.547667000	-3.087083000
H	2.179928000	3.045180000	-0.960552000
H	0.747239000	5.310514000	2.066394000
H	-0.003055000	3.193918000	2.944424000
H	2.258776000	3.422284000	2.077349000
H	-1.983136000	3.346451000	0.324706000
H	-1.959856000	2.726232000	-2.290722000
H	-2.944944000	-2.665165000	-2.597255000
H	-3.567031000	-0.059655000	-2.339989000
H	-6.028901000	1.093525000	0.668617000

H	-3.962787000	1.343198000	1.884266000
H	-4.168097000	2.209373000	-0.385460000
H	-3.999652000	-1.930259000	1.578134000
H	-3.142995000	-3.805287000	-0.155089000
H	2.855038000	-3.119314000	2.140687000
H	3.768249000	-0.591991000	2.043823000
H	6.185872000	0.629142000	-0.748429000
H	4.298612000	0.900146000	-2.234028000
H	4.244330000	1.824120000	0.020421000
H	3.722550000	-2.166454000	-2.029981000
H	2.839377000	-4.099803000	-0.373524000
C	0.879767000	0.149045000	1.341129000
C	-0.358717000	-0.053975000	1.995942000
C	-1.181867000	-1.234330000	2.044172000
C	-0.849741000	-2.500743000	1.466357000
C	0.024545000	-2.596794000	0.381399000
N	1.037545000	-0.130997000	-1.356630000
H	-2.019455000	-1.192181000	2.738443000
H	1.460763000	0.985781000	1.755570000
H	-0.749339000	0.757904000	2.623155000
H	-1.448187000	-3.355944000	1.799157000

TS′$_{H3m}$

Ti	-1.623783000	-1.208087000	-0.344940000
Ti	1.737105000	-1.103877000	0.017522000
Ti	0.037303000	1.091726000	-0.615491000
C	-3.906469000	-0.742737000	-0.201760000
C	-3.725723000	-2.107770000	0.191645000
C	-3.221032000	-2.849936000	-0.912139000
C	-3.097735000	-1.961997000	-2.018506000
C	-3.506105000	-0.669538000	-1.587777000
H	0.369798000	-3.482561000	-0.386791000
H	-1.644101000	0.586239000	-0.111138000
C	4.085520000	-0.806225000	-0.001528000
C	3.708309000	-0.886516000	1.382834000
C	3.231884000	-2.195650000	1.660693000
C	3.283390000	-2.944607000	0.453919000
C	3.802491000	-2.096348000	-0.566363000
H	-0.756126000	-1.721705000	-1.720560000
H	-0.835808000	-0.509940000	-1.786716000
C	-0.106012000	3.364220000	0.182640000

C	-1.249035000	3.140848000	-0.655541000
C	-0.805025000	2.852184000	-1.978879000
C	0.617930000	2.922090000	-1.990283000
C	1.045575000	3.217341000	-0.669077000
Si	-4.591096000	0.648609000	0.850071000
Si	4.814185000	0.666658000	-0.904113000
Si	-0.106839000	3.878170000	1.985598000
H	1.262518000	2.706749000	-2.835437000
H	2.080798000	3.287080000	-0.347298000
H	-0.286857000	5.353870000	2.115790000
H	-1.219532000	3.212570000	2.720517000
H	1.193561000	3.511363000	2.614728000
H	-2.283976000	3.154780000	-0.330122000
H	-1.439005000	2.601991000	-2.824228000
H	-2.725309000	-2.220589000	-3.003028000
H	-3.503086000	0.228789000	-2.195277000
H	-6.075091000	0.526603000	0.973392000
H	-4.020149000	0.617010000	2.226511000
H	-4.289967000	1.958956000	0.211755000
H	-3.909054000	-2.508626000	1.183920000
H	-2.940368000	-3.898716000	-0.904767000
H	2.832542000	-2.540159000	2.609549000
H	3.764212000	-0.073022000	2.099862000
H	6.302579000	0.679505000	-0.765451000
H	4.482820000	0.606576000	-2.352916000
H	4.304842000	1.938807000	-0.315862000
H	3.944808000	-2.369960000	-1.608450000
H	2.963444000	-3.974363000	0.327386000
C	0.735432000	0.187838000	1.336555000
C	-0.508899000	-0.185956000	1.937162000
C	-1.177141000	-1.446342000	1.920156000
C	-0.760001000	-2.623935000	1.207401000
C	0.138712000	-2.542548000	0.128423000
N	1.305022000	0.025948000	-1.315258000
H	-2.051467000	-1.526570000	2.565357000
H	1.206154000	1.053686000	1.826630000
H	-1.015248000	0.555479000	2.566562000
H	-1.305738000	-3.544890000	1.437644000

3m

Ti	0.879479000	-1.213649000	0.284181000

Ti	-1.698827000	0.140728000	0.103038000
Ti	0.810437000	1.419940000	-0.657145000
Si	3.940834000	-3.130183000	-0.849027000
Si	-4.648535000	-1.971622000	-0.735977000
Si	1.540478000	3.308173000	2.316081000
N	0.093171000	0.420963000	0.762772000
C	-1.105706000	-1.740506000	-0.509424000
H	-1.530329000	-2.704239000	-0.207595000
C	-0.157166000	-1.821624000	-1.595855000
H	0.195289000	-2.794166000	-1.958305000
C	0.346032000	-0.695896000	-2.332015000
H	1.164474000	-0.924054000	-3.018834000
C	-0.211590000	0.621557000	-2.473311000
H	0.105206000	1.152611000	-3.378812000
C	-1.180011000	1.211627000	-1.578704000
H	-1.670697000	2.110818000	-1.966635000
C	2.670036000	-2.820061000	0.498864000
C	1.438187000	-3.518844000	0.709459000
C	0.777334000	-2.969329000	1.843478000
C	1.604661000	-1.925403000	2.367964000
C	2.758313000	-1.832405000	1.541507000
C	-3.989320000	-0.503845000	0.226079000
C	-3.936356000	0.868213000	-0.206773000
C	-3.390343000	1.661345000	0.841348000
C	-3.091662000	0.800226000	1.935661000
C	-3.457303000	-0.520919000	1.561618000
C	1.631057000	3.258253000	0.441629000
C	0.645239000	3.746888000	-0.490663000
C	1.117198000	3.538060000	-1.815350000
C	2.389216000	2.911269000	-1.736709000
C	2.708165000	2.734397000	-0.359171000
H	2.039444000	0.007385000	-0.534550000
H	4.848017000	-4.251627000	-0.465724000
H	4.762467000	-1.905612000	-1.053704000
H	3.264440000	-3.500656000	-2.124706000
H	3.559543000	-1.108095000	1.653052000
H	1.373258000	-1.287828000	3.215079000
H	-0.184225000	-3.283163000	2.236124000
H	1.045417000	-4.311079000	0.078887000
H	2.992823000	2.580887000	-2.577318000
H	3.608330000	2.263371000	0.023277000

H	2.268425000	2.146602000	2.896760000
H	2.178949000	4.556827000	2.832465000
H	0.119899000	3.300140000	2.760979000
H	-0.310545000	4.188067000	-0.224744000
H	0.574241000	3.768692000	-2.726711000
H	-4.294825000	-1.869287000	-2.178034000
H	-4.098785000	-3.233215000	-0.163324000
H	-6.138710000	-2.039177000	-0.632239000
H	-3.319543000	-1.410072000	2.171219000
H	-3.189387000	2.727829000	0.797414000
H	-2.621206000	1.094250000	2.869255000
H	-4.252894000	1.236471000	-1.177200000

TS_{FG}

Ti	-0.881343000	-1.278536000	-0.278378000
Ti	1.896573000	-0.531060000	0.685904000
Ti	-0.203341000	1.284630000	0.114016000
C	-2.990805000	-2.262321000	-0.696458000
C	-2.049700000	-3.294424000	-0.341317000
C	-1.030697000	-3.360381000	-1.338489000
C	-1.326696000	-2.362522000	-2.318620000
C	-2.522035000	-1.700629000	-1.930404000
H	0.771635000	-2.878224000	1.897090000
H	-1.297975000	0.312656000	-0.979748000
C	3.938728000	-0.408724000	-0.486556000
C	4.207082000	-0.555941000	0.921587000
C	3.782170000	-1.849127000	1.339045000
C	3.273473000	-2.532357000	0.195741000
C	3.360639000	-1.656692000	-0.913626000
H	0.607228000	-2.175013000	0.143395000
H	0.708340000	-0.151648000	-0.733832000
C	-1.480653000	3.175240000	-0.476683000
C	-1.017008000	2.571531000	-1.698343000
C	0.401359000	2.639950000	-1.746971000
C	0.849425000	3.309015000	-0.572774000
C	-0.297395000	3.637927000	0.202689000
Si	-4.580580000	-1.824883000	0.195746000
Si	4.367695000	1.054968000	-1.575121000
Si	-3.261178000	3.428160000	0.040996000
H	1.882586000	3.483594000	-0.292292000
H	-0.284266000	4.152273000	1.158651000

H	−3.774057000	4.758904000	−0.406299000
H	−4.124181000	2.375520000	−0.563315000
H	−3.383947000	3.379558000	1.527279000
H	−1.649136000	2.111663000	−2.450177000
H	1.034233000	2.225740000	−2.524873000
H	−0.720453000	−2.121971000	−3.187794000
H	−2.989558000	−0.880178000	−2.465163000
H	−5.665264000	−2.788671000	−0.155472000
H	−4.383367000	−1.887392000	1.673348000
H	−5.017947000	−0.455866000	−0.191281000
H	−2.110523000	−3.928176000	0.538303000
H	−0.202639000	−4.060673000	−1.364886000
H	3.827694000	−2.243136000	2.350167000
H	4.633275000	0.208662000	1.564867000
H	5.776013000	0.956817000	−2.065204000
H	3.470756000	1.083285000	−2.764717000
H	4.240415000	2.331019000	−0.817009000
H	3.017865000	−1.874752000	−1.921138000
H	2.842753000	−3.528887000	0.190474000
C	0.396565000	1.556320000	2.328889000
C	−0.936968000	1.127374000	2.257385000
C	−1.382808000	−0.127122000	1.614378000
C	−0.819068000	−1.490878000	1.888483000
C	0.515335000	−1.868432000	1.549734000
N	1.334074000	0.965899000	1.541683000
H	−2.478807000	−0.146535000	1.638317000
H	0.652802000	2.458778000	2.895339000
H	−1.700922000	1.768501000	2.698868000
H	−1.471388000	−2.200146000	2.412572000

G

Ti	−0.529031000	1.565419000	−0.025394000
Ti	1.941143000	0.442410000	0.107654000
Ti	−0.693842000	−1.182783000	0.292574000
C	−2.623230000	2.640085000	0.131297000
C	−1.761342000	3.444351000	−0.693808000
C	−0.674002000	3.908653000	0.098536000
C	−0.858026000	3.422395000	1.428618000
C	−2.042710000	2.647545000	1.449221000
H	1.758609000	0.544082000	−1.872560000
H	−0.919199000	0.331991000	1.196714000

C	4.199651000	-0.250188000	0.067802000
C	4.125037000	0.959021000	-0.698758000
C	3.756191000	2.039111000	0.152685000
C	3.600842000	1.518865000	1.470429000
C	3.859045000	0.121537000	1.419377000
H	1.516779000	2.334043000	-2.030437000
H	0.940188000	1.581138000	1.081505000
C	-2.244410000	-2.872760000	0.584453000
C	-2.554940000	-1.833540000	1.529483000
C	-1.482611000	-1.727759000	2.467902000
C	-0.508514000	-2.705693000	2.134458000
C	-0.958016000	-3.403080000	0.980068000
Si	-4.256768000	1.866420000	-0.360444000
Si	4.625925000	-1.968280000	-0.555309000
Si	-3.307074000	-3.493381000	-0.826697000
H	0.447413000	-2.846492000	2.629768000
H	-0.430573000	-4.211488000	0.484132000
H	-4.409848000	-4.368974000	-0.327986000
H	-3.928359000	-2.361024000	-1.572278000
H	-2.465480000	-4.294868000	-1.760469000
H	-3.443055000	-1.209420000	1.518448000
H	-1.411854000	-1.005323000	3.273572000
H	-0.181434000	3.575670000	2.262491000
H	-2.434620000	2.115736000	2.310079000
H	-5.349040000	2.885917000	-0.333677000
H	-4.196418000	1.313848000	-1.743663000
H	-4.618120000	0.783241000	0.595764000
H	-1.920448000	3.671279000	-1.742634000
H	0.160523000	4.511019000	-0.248394000
H	3.594796000	3.068615000	-0.150070000
H	4.302122000	1.035760000	-1.768380000
H	6.103605000	-2.186337000	-0.521737000
H	3.986121000	-2.996033000	0.310542000
H	4.169853000	-2.126973000	-1.964022000
H	3.787685000	-0.565304000	2.259080000
H	3.297117000	2.081923000	2.346867000
C	0.592519000	-1.989356000	-1.285268000
C	-0.553658000	-1.441188000	-1.914353000
C	-1.041301000	-0.048793000	-1.691752000
C	-0.320801000	1.201569000	-2.082258000
C	1.056509000	1.417031000	-1.647098000

N	1.252338000	-1.201616000	-0.387232000
H	-2.092187000	0.011469000	-1.998604000
H	0.817831000	-3.056497000	-1.369865000
H	-1.174163000	-2.105802000	-2.517785000
H	-0.742730000	1.795649000	-2.894028000

TS$_{GH}$

Ti	0.348733000	-1.589182000	0.129918000
Ti	-2.034764000	-0.387152000	-0.408001000
Ti	0.761291000	1.238146000	0.319981000
C	2.346914000	-2.804447000	0.512771000
C	1.417498000	-3.651710000	-0.186479000
C	0.288436000	-3.878231000	0.645260000
C	0.503055000	-3.190050000	1.879161000
C	1.760636000	-2.543103000	1.799765000
H	-1.525388000	-0.814641000	-2.285076000
H	0.699761000	-0.254705000	1.331300000
C	-4.107031000	0.549256000	0.282522000
C	-4.183572000	0.225640000	-1.116733000
C	-4.134939000	-1.194908000	-1.277488000
C	-3.976819000	-1.768264000	0.006898000
C	-3.934414000	-0.701850000	0.958431000
H	-1.249375000	-2.593633000	-2.137546000
H	-1.373365000	-1.606235000	0.784144000
C	2.481593000	2.778437000	0.175065000
C	2.868686000	1.795372000	1.155624000
C	1.968733000	1.863995000	2.262429000
C	1.024667000	2.892122000	2.000179000
C	1.320763000	3.447668000	0.724418000
Si	4.038991000	-2.263592000	-0.080725000
Si	-4.091823000	2.266458000	1.041142000
Si	3.372132000	3.232324000	-1.404433000
H	0.175553000	3.149917000	2.625740000
H	0.760194000	4.238545000	0.234399000
H	4.547536000	4.114410000	-1.132272000
H	3.877095000	2.016895000	-2.106433000
H	2.444788000	3.973620000	-2.306514000
H	3.697169000	1.100094000	1.061332000
H	1.981786000	1.218434000	3.134194000
H	-0.193331000	-3.136008000	2.709397000
H	2.194294000	-1.913175000	2.569420000

H	5.011961000	-3.395118000	-0.010177000
H	3.993887000	-1.802794000	-1.498651000
H	4.547517000	-1.163204000	0.784412000
H	1.557738000	-4.056553000	-1.183624000
H	-0.598333000	-4.446691000	0.380428000
H	-4.170242000	-1.731110000	-2.221495000
H	-4.282273000	0.945257000	-1.925868000
H	-5.481961000	2.674820000	1.409819000
H	-3.265921000	2.279149000	2.277892000
H	-3.576964000	3.253345000	0.052175000
H	-3.784387000	-0.827158000	2.027015000
H	-3.848657000	-2.822761000	0.228400000
C	-0.733733000	1.708765000	-1.218952000
C	0.445539000	1.198686000	-1.851041000
C	1.093631000	-0.097004000	-1.530716000
C	0.561042000	-1.411094000	-1.966784000
C	-0.861735000	-1.621625000	-1.808396000
N	-1.193649000	1.211345000	0.036007000
H	2.171960000	-0.033049000	-1.727541000
H	-1.068213000	2.711658000	-1.503646000
H	0.905532000	1.842844000	-2.603094000
H	1.187533000	-2.040038000	-2.601330000

H

Ti	-0.047078000	1.534485000	0.051274000
Ti	2.130117000	0.021197000	-0.568021000
Ti	-1.239441000	-0.960114000	0.332597000
C	-1.553453000	3.287007000	0.475489000
C	-0.498186000	3.811903000	-0.344528000
C	0.719354000	3.741299000	0.379643000
C	0.432746000	3.212075000	1.681577000
C	-0.949773000	2.945085000	1.742625000
H	1.512477000	0.381160000	-2.460684000
H	-1.318867000	0.672713000	1.057563000
C	3.824779000	-1.170600000	0.547936000
C	3.805868000	-1.599742000	-0.827326000
C	4.186424000	-0.503985000	-1.656786000
C	4.434399000	0.619087000	-0.815131000
C	4.206372000	0.212844000	0.531487000
H	1.662599000	2.187530000	-2.309900000
H	1.748331000	1.375665000	0.560111000

C	-2.918570000	-2.603686000	0.094712000
C	-3.566718000	-1.427352000	0.610769000
C	-3.040445000	-1.132548000	1.899115000
C	-2.060005000	-2.115990000	2.213283000
C	-1.979642000	-3.015199000	1.109370000
Si	-3.368439000	3.134380000	0.034133000
Si	3.377639000	-2.198452000	2.056202000
Si	-3.279045000	-3.471766000	-1.521295000
H	-1.450615000	-2.147465000	3.111370000
H	-1.307167000	-3.866090000	1.036332000
H	-4.394514000	-4.455545000	-1.373693000
H	-3.678796000	-2.483818000	-2.563886000
H	-2.073501000	-4.219278000	-1.981813000
H	-4.315161000	-0.837494000	0.088846000
H	-3.312613000	-0.283828000	2.517607000
H	1.160210000	3.011938000	2.461011000
H	-1.471919000	2.507861000	2.586890000
H	-4.064368000	4.446276000	0.203923000
H	-3.537883000	2.720524000	-1.388510000
H	-4.025898000	2.143164000	0.929558000
H	-0.610153000	4.189141000	-1.355225000
H	1.701242000	4.024951000	0.014620000
H	4.254622000	-0.519336000	-2.741041000
H	3.548298000	-2.594933000	-1.176395000
H	4.589342000	-2.900027000	2.578323000
H	2.847510000	-1.320385000	3.133597000
H	2.373493000	-3.233456000	1.685967000
H	4.285858000	0.854092000	1.403630000
H	4.710817000	1.617278000	-1.142464000
C	0.548409000	-1.470051000	-0.891944000
C	-0.652689000	-1.144459000	-1.686845000
C	-1.153060000	0.242378000	-1.681196000
C	-0.354675000	1.426066000	-2.046164000
C	1.093658000	1.319910000	-1.957670000
N	0.638136000	-0.687546000	0.396691000
H	-2.203896000	0.331701000	-1.975835000
H	0.825666000	-2.527073000	-0.813742000
H	-1.016794000	-1.872646000	-2.411863000
H	-0.835350000	2.205208000	-2.636885000

TS_{HI}

Ti	-0.190133000	1.469473000	0.155373000
Ti	2.086128000	0.152493000	-0.498577000
Ti	-0.965765000	-1.176161000	0.177102000
C	-1.874471000	2.993543000	0.748459000
C	-0.915804000	3.702841000	-0.047901000
C	0.323330000	3.719695000	0.642954000
C	0.141929000	3.059374000	1.903119000
C	-1.195969000	2.621659000	1.968895000
H	1.545902000	0.780501000	-2.442776000
H	-1.477317000	0.382086000	0.902858000
C	4.028696000	-0.868350000	0.348296000
C	4.038446000	-1.039455000	-1.081754000
C	4.191222000	0.234824000	-1.697682000
C	4.254523000	1.215737000	-0.668737000
C	4.155453000	0.541273000	0.582568000
H	1.452627000	2.531686000	-2.001546000
H	1.560885000	1.433287000	0.697251000
C	-3.000971000	-2.497909000	0.092726000
C	-2.869140000	-1.935098000	1.408232000
C	-1.695944000	-2.454438000	2.022859000
C	-1.079236000	-3.349448000	1.097836000
C	-1.871660000	-3.369558000	-0.083667000
Si	-3.670463000	2.648470000	0.333818000
Si	3.847550000	-2.212698000	1.650067000
Si	-4.400443000	-2.204899000	-1.118270000
H	-0.147595000	-3.884856000	1.253672000
H	-1.655915000	-3.949933000	-0.976424000
H	-5.094892000	-0.928878000	-0.790566000
H	-3.878706000	-2.137982000	-2.515027000
H	-5.399743000	-3.312885000	-1.061372000
H	-3.541930000	-1.206696000	1.850666000
H	-1.312613000	-2.182594000	3.001262000
H	0.910415000	2.892364000	2.650062000
H	-1.635867000	2.049241000	2.778993000
H	-4.498774000	3.876424000	0.533572000
H	-3.823159000	2.231503000	-1.088932000
H	-4.193311000	1.583462000	1.232714000
H	-1.100719000	4.140027000	-1.023244000
H	1.254727000	4.143691000	0.280810000
H	4.216317000	0.425650000	-2.766833000
H	3.944377000	-1.985288000	-1.606404000

H	5.188284000	-2.773057000	1.997102000
H	3.242112000	-1.645616000	2.884485000
H	3.009089000	-3.324168000	1.121504000
H	4.145757000	1.021227000	1.556310000
H	4.329714000	2.289669000	-0.810543000
C	0.863618000	-1.371815000	-1.349906000
C	-0.425906000	-1.126204000	-1.920033000
C	-1.091282000	0.172583000	-1.778601000
C	-0.456172000	1.488204000	-1.939245000
C	1.005087000	1.548668000	-1.818164000
N	0.769251000	-0.754257000	0.516379000
H	-2.151659000	0.169407000	-2.047253000
H	1.194922000	-2.416809000	-1.350810000
H	-0.947001000	-1.932847000	-2.441284000
H	-1.013229000	2.254499000	-2.478802000

I

Ti	-0.994629000	-1.429288000	-0.141591000
Ti	1.611424000	-0.980177000	0.217536000
Ti	-0.169596000	1.255710000	-0.290676000
C	-3.277258000	-1.987110000	-0.542542000
C	-2.690643000	-3.087512000	0.163462000
C	-1.651631000	-3.642401000	-0.627175000
C	-1.591414000	-2.910573000	-1.858932000
C	-2.589391000	-1.909613000	-1.806788000
H	0.870194000	-1.472646000	2.464989000
H	-1.724863000	0.304654000	-0.411203000
C	3.916362000	-0.494355000	-0.093993000
C	3.772932000	-0.955524000	1.261628000
C	3.349216000	-2.311637000	1.243204000
C	3.213841000	-2.714067000	-0.120262000
C	3.563256000	-1.604274000	-0.937034000
H	-0.061659000	-2.921793000	1.932361000
H	0.571901000	-2.387544000	-0.391098000
C	-0.992426000	3.521261000	-0.261285000
C	-1.430810000	2.863953000	-1.466437000
C	-0.292497000	2.543867000	-2.258043000
C	0.866445000	2.979126000	-1.552920000
C	0.441752000	3.573653000	-0.333458000
Si	-4.667347000	-0.868549000	0.037468000
Si	4.582202000	1.160880000	-0.667712000

Si	-2.067552000	4.257359000	1.086913000
H	1.894335000	2.836087000	-1.870142000
H	1.097158000	3.981999000	0.430084000
H	-2.688013000	5.535827000	0.629432000
H	-3.169331000	3.330160000	1.473424000
H	-1.225377000	4.546356000	2.282350000
H	-2.461758000	2.643724000	-1.726532000
H	-0.299575000	2.005876000	-3.200454000
H	-0.878067000	-3.067604000	-2.660728000
H	-2.759637000	-1.149668000	-2.563896000
H	-5.986628000	-1.557441000	-0.089966000
H	-4.486298000	-0.501468000	1.470841000
H	-4.697673000	0.361322000	-0.800704000
H	-2.965067000	-3.417574000	1.161016000
H	-1.000086000	-4.463810000	-0.346419000
H	3.130074000	-2.925260000	2.112078000
H	3.949721000	-0.361660000	2.152639000
H	6.075016000	1.136163000	-0.707336000
H	4.093013000	1.457058000	-2.044032000
H	4.171099000	2.254985000	0.256519000
H	3.531085000	-1.586162000	-2.022521000
H	2.880079000	-3.685359000	-0.469675000
C	1.248592000	0.805853000	1.354314000
C	0.022821000	1.164067000	1.998683000
C	-1.150507000	0.356162000	2.020615000
C	-1.221406000	-1.086036000	1.976922000
C	0.042673000	-1.834623000	1.835320000
N	0.476946000	-0.257556000	-1.061552000
H	-2.082941000	0.865149000	2.274460000
H	2.089726000	1.469965000	1.589185000
H	-0.101060000	2.168487000	2.427007000
H	-2.104020000	-1.531293000	2.438207000

TS_{I3m}

Ti	-1.324861000	-1.511433000	-0.235091000
Ti	1.664447000	-1.035466000	0.297697000
Ti	-0.130818000	1.147491000	-0.231792000
C	-3.682704000	-1.239522000	-0.407294000
C	-3.450813000	-2.184285000	0.637799000
C	-2.821324000	-3.340266000	0.090781000
C	-2.680269000	-3.136224000	-1.314988000

C	-3.178845000	-1.842880000	-1.617198000
H	0.334442000	-3.250073000	1.698197000
H	-1.770021000	0.376147000	-0.239854000
C	3.918537000	-0.792490000	-0.347867000
C	3.930515000	-1.100650000	1.058390000
C	3.424901000	-2.415591000	1.241674000
C	3.100473000	-2.945154000	-0.041590000
C	3.401653000	-1.958134000	-1.015337000
H	-0.070769000	-2.749870000	-0.060395000
H	-0.440147000	-2.711865000	-1.149585000
C	-0.403574000	3.539873000	-0.099558000
C	-1.356763000	3.029450000	-1.045957000
C	-0.658234000	2.448145000	-2.140605000
C	0.735713000	2.578969000	-1.890255000
C	0.897201000	3.239359000	-0.638430000
Si	-4.543566000	0.420487000	-0.269751000
Si	4.574558000	0.764240000	-1.160825000
Si	-0.773135000	4.505057000	1.464319000
H	1.533788000	2.192874000	-2.516549000
H	1.845894000	3.482114000	-0.171113000
H	-0.997454000	5.948916000	1.157675000
H	-1.998892000	3.979706000	2.130520000
H	0.382203000	4.405335000	2.401107000
H	-2.436235000	3.064259000	-0.938463000
H	-1.108909000	1.936277000	-2.985308000
H	-2.250970000	-3.834577000	-2.023819000
H	-3.168788000	-1.375659000	-2.598190000
H	-6.025938000	0.227187000	-0.288464000
H	-4.191663000	1.111874000	1.000986000
H	-4.182898000	1.273553000	-1.435203000
H	-3.713631000	-2.040990000	1.680468000
H	-2.502194000	-4.219375000	0.642046000
H	3.273839000	-2.915084000	2.193486000
H	4.254547000	-0.431676000	1.848813000
H	6.042051000	0.646173000	-1.410424000
H	3.903036000	0.983025000	-2.473184000
H	4.354468000	1.949146000	-0.284105000
H	3.232630000	-2.051094000	-2.084270000
H	2.650969000	-3.915128000	-0.235170000
C	1.430682000	0.736622000	1.291373000
C	0.185439000	0.934182000	1.997847000

C	-0.850057000	-0.030575000	2.128994000
C	-0.842493000	-1.457103000	1.928378000
C	0.201700000	-2.249832000	1.270865000
N	0.313551000	-0.509648000	-0.897452000
H	-1.769056000	0.344327000	2.586906000
H	2.193294000	1.495571000	1.494541000
H	-0.005921000	1.876873000	2.524025000
H	-1.575822000	-1.983782000	2.545030000

Supplementary References

1. Shima, T. *et al.* Dinitrogen cleavage and hydrogenation by a trinuclear titanium polyhydride complex. *Science* **340,** 1549−1552 (2013).

2. Yandulov, D. V. & Schrock, R. R. Catalytic reduction of dinitrogen to ammonia at a single molybdenum center. *Science* **301,** 76−78 (2003).

3. Weatherburn, M. W. Phenol-hypochlorite reaction for determination of ammonia. *Anal. Chem.* **39**, 971–974 (1967).

4. APEX2 v2013.2-0; Bruker AXS Inc., Madison, WI, 2007.

5. G. M. Sheldrick, A short history of SHELX. *Acta Cryst. A* **64,** 112–122 (2008).

6. L. J. J. Farrugia, WinGX suite for small-molecule single-crystal crystallography. *J. Appl. Cryst.* **32,** 837–838 (1999).

7. Tao, J., Perdew, J., Staroverov, V., Scuseria, G. Climbing the density functional ladder: nonempirical meta-generalized gradient approximation designed for molecules and solids. *Phys. Rev. Lett.* **91,** 146401 (2003).

8. Kang, X. *et al.* Mechanistic insights into ring cleavage and contraction of benzene over a titanium hydride cluster. *J. Am. Chem. Soc.* **138,** 11550−11559 (2016).

9. Dolg, M., Wedig, U., Stoll, H. & Preuss, H. Energy-adjusted *ab initio* pseudopotentials for the first row transition elements. *J. Chem. Phys.* **86,** 866−872 (1987).

10. Bergner, A., Dolg, M., Küchle, W., Stoll, H. & Preuss, H. *Ab initio* energy-adjusted pseudopotentials for elements of groups 13–17. *Mol. Phys.* **80,** 1431−1441 (1993).

11. Höllwarth, A. *et al.* A set of d-polarization functions for pseudo-potential basis sets of the main group elements Al−Bi and f-type polarization functions for Zn, Cd, Hg. *Chem. Phys. Lett.* **208,** 237−240 (1993).

12. Barone, V. & Cossi, M. Quantum calculation of molecular energies and energy gradients in solution by a conductor solvent model. *J. Phys. Chem. A* **102,** 1995−2001 (1998).

13. Cossi, M., Rega, N., Scalmani, G. & Barone, V. Energies, structures, and electronic properties of molecules in solution with the C-PCM solvation model. *J. Comput. Chem.* **24,** 669−681 (2003).

14. Grimme, S., Ehrlich, S. & Goerigk, L. Effect of the damping function in dispersion corrected density functional theory. *J. Comput. Chem*. **32,** 1456−1465 (2011).

15. Bühl, M. & Kabrede, H. Geometries of transition-metal complexes from density-functional

theory. *J. Chem. Theory Comput.* **2,** 1282−1290 (2006).

16. Zhao, Y. & Truhlar, D. G. Comparative assessment of density functional methods for 3d transition-metal chemistry. *J. Chem. Phys*. **124,** 224105 (2006).
17. Furche, F. & Perdew, J. P. The Performance of semilocal and hybrid density functionals in 3d transition-metal chemistry. *J. Chem. Phys*. **124,** 044103 (2006).
18. Frisch, M. J. *et al. Gaussian 09, Revision D.01*; Gaussian, Inc., Wallingford, CT, 2013.
